# Global Diversity and Review of Siphonophorae (Cnidaria: Hydrozoa)

**DOI:** 10.1371/journal.pone.0087737

**Published:** 2014-02-06

**Authors:** Gillian M. Mapstone

**Affiliations:** Department of Life Sciences, The Natural History Museum, London, United Kingdom; Sars International Centre for Marine Molecular Biology, Norway

## Abstract

In this review the history of discovery of siphonophores, from the first formal description by Carl Linnaeus in 1785 to the present, is summarized, and species richness together with a summary of world-wide distribution of this pelagic group within the clade Hydrozoa discussed. Siphonophores exhibit three basic body plans which are briefly explained and figured, whilst other atypical body plans are also noted. Currently, 175 valid siphonophore species are recognized in the latest WoRMS world list, including 16 families and 65 genera. Much new information since the last review in 1987 is revealed from the first molecular analysis of the group, enabling identification of some new morphological characters diagnostic for physonect siphonophores. Ten types of nematocysts (stinging cells) are identified in siphonophores, more than in any other cnidarian; these are incorporated into batteries in the side branches of the tentacles in most species (here termed tentilla), and tentilla are reviewed in the last section of this paper. Their discharge mechanisms are explained and also how the tentilla of several physonect siphonophores are modified into lures. Of particular interest is the recent discovery of a previously unknown red fluorescent lure in the tentilla of the deep sea physonect *Erenna,* the first described example of emission of red light by an invertebrate to attract prey.

## Introduction

Siphonophores are a small group of complex fragile polymorphic and mostly elongate colonial hydrozoans currently comprising 175 valid species [1] (the present author is the main editor of the Siphonophora section of the WoRMS world list). Most siphonophore species are pelagic and restricted to oceanic waters, and generally live well below the surface to avoid turbulence. Small active species inhabit the epipelagic zone (0-c. 300 m), where they lie in wait for copepods and other zooplankton, and rapidly spread their tentacles to entrap prey. Larger, though mostly more fragile, species live in the deeper and tranquil mesopelagic zone (300–1000 m), where they passively extend an enormous feeding net of tentacles to ensnare prey [2], [3]. A few genera are neritic with most of their species restricted to coastal waters (*Muggiaea*, *Sphaeronectes*). One family, the Rhodaliidae, is epibenthic with a short corm-like stem and tentacles that extend out in all directions for anchorage to the substrate [4]. Siphonophores make a significant contribution to complex trophic links in the deep sea ‘jelly web’, of which gelatinous zooplankton can contribute up to 25% of the total pelagic biomass [3]. The geographical distribution of most siphonophores is cosmopolitan with species inhabiting all oceans [5]. However, some are restricted to particular latitudinal ranges or oceanic areas, and a few are so far known only from a single location. Siphonophores are extremely difficult to capture, with the best specimens collected and observed from submersibles or with blue-water SCUBA equipment.

Siphonophores are holoplanktonic, except for rhodaliids which can transiently attach their tentacles to the substrate, and thus lack the true benthic stage that is characteristic of the life cycle of many hydromedusae and other colonial cnidarians. Larvae are sometimes collected, and a few species have been successfully reared in the laboratory [40], but larvae of most species are still unknown. The yolky planula soon develops a stem and in most species begins budding zooids from two growth zones [6]. As the stem extends, more zooids form, the colony matures and various morphological axes can be identified [6], [7]. Upon maturity, this asexual life stage may release egg or sperm masses directly into the water [8], or release gametes either from sexual gonophores that remain attached to the stem, or form eudoxids (sexual life stages) from the end of the stem which are later released. These life stages are explained in a recently published glossary of siphonophore terminology [6].

For many decades, siphonophore systematics was based primarily on the classification of Totton [9], including the last review of the group [2]. Totton divided siphonophores into three suborders: Cystonectae, Physonectae and Calycophorae. More recently, however, the first molecular analysis of siphonophores [10] revealed a major new phylogeny in which cystonects, without nectosomal swimming bells, are sister to all other siphonophores with bells. This latter clade is known as the bell-bearers, or Codonophora, and these taxa differ from the Cystonecta in one important respect: the feeding and sexual zooids (gastrozooids and gonodendra, or gonophores) of each iterative group (cormidium) on the stem form from a single probud, except for a few secondary zooids which arise from primary zooids in some species [11]. In cystonects gastrozooids and gonodendra develop separately and directly on the stem, not from a probud [8]. This important difference is reflected in the new phylogeny, as summarized by Mapstone (p. 75 [6]).

Previously unknown red fluorescent lures have also been discovered in a new deep sea physonect species of the genus *Erenna* [12]. In addition, new families and sub-families of siphonophores have been identified and others reviewed [13], [14], [15], [16], [17], [18], [19], several new species have been described [1], and body axes standardized for all siphonophores [6], [7]. All these new findings are discussed below, together with a summary of the history of discovery of siphonophores and, for the first time, an assessment of siphonophore nematocysts, tentilla and lures.

## Results and Discussion

### History of Discovery

**Figure 1:**
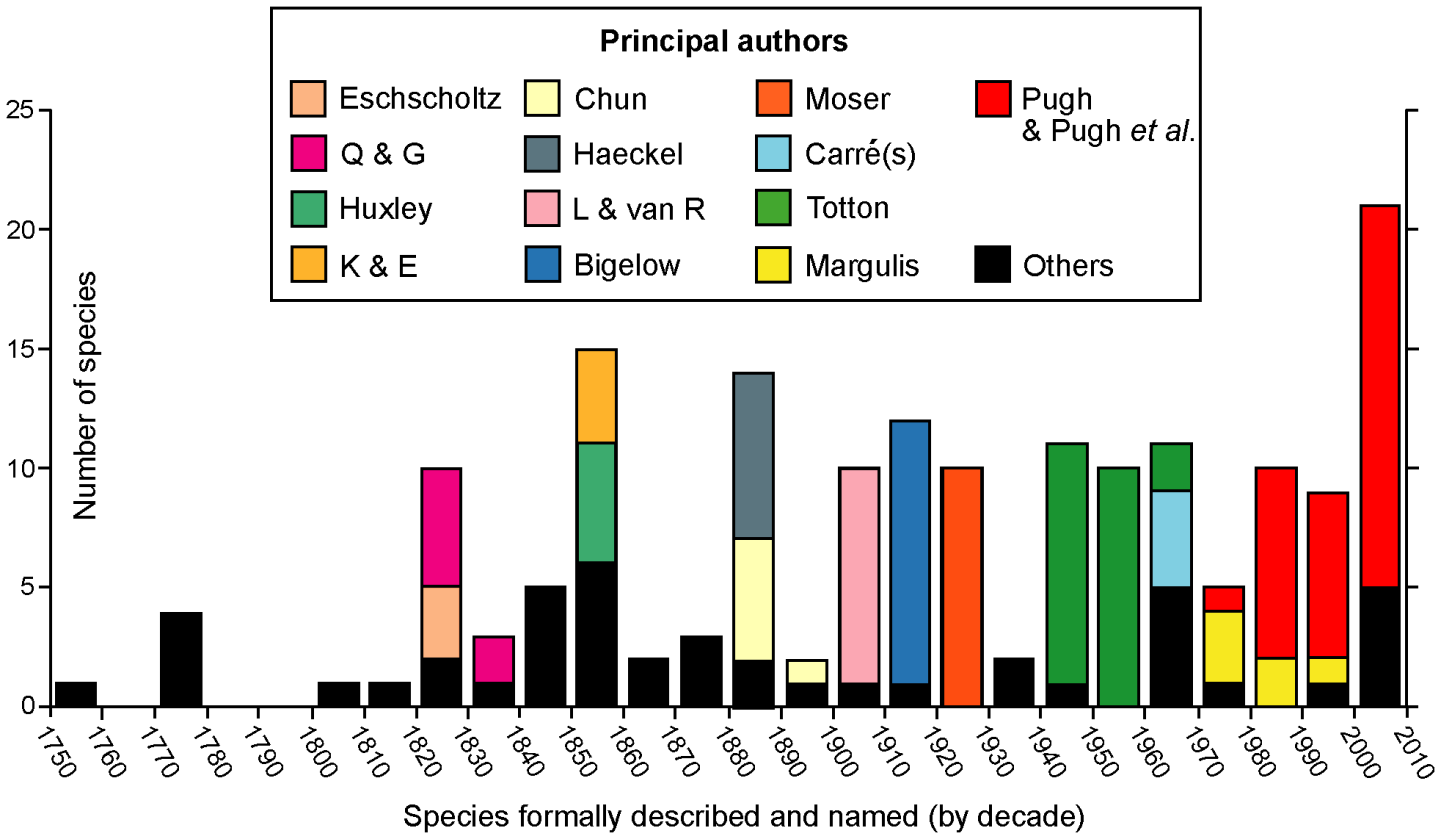
History of siphonophore research. Principle researchers and others from mid-18^th^ century to the present. Authors identified only by initials are Q & G: Quoy and Gaimard, K & E: Keferstein and Ehlers, and L & van R: Lens and van Riemsdijk.

Most non-specialist biologists know only one species of siphonophore, the Portuguese Man O’War (*Physalia physalis*), because it has a large and colourful float on the sea surface propelled by the wind. Not surprisingly, this was the first siphonophore to be formally described and introduced, as long ago as 1758, by Carl Linnaeus. Only four additional valid species were described during the rest of that century. In the nineteenth century, however, 56 new species were introduced (Figure 1).

The first half of the 19^th^ century saw a flowering of voyages of discovery. Collection of fauna and flora provided ships with a free passport to otherwise hostile anchorages controlled by various European maritime powers; distant lands were discovered and charts made of their coastal waters. Marine fauna collected often included the almost exclusively holoplanktonic group Siphonophora. Specimens were found in surface waters in these early days, some of which arrived there via upwelling events. Eschscholtz (Figure 1) circumnavigated the world twice in the Russian brigs Rurik (1816–1818) and Enterprise (1823–1826) and brought back the first specimens of *Agalma okeni* and *Chelophyes appendiculata* from the tropical north Pacific Ocean. He included formal descriptions of these species, and another 12 valid species he had introduced earlier in his 1829 volume ‘*System der Acalephen*’ [20]. All were placed in a new order Siphonophora, which at that time also included the “chondrophores” (*Porpita* and *Velella*, see below). Eschscholtz’s 1829 work was published just after the first observations on siphonophores by Quoy and Gaimard in 1827. The latter authors sailed to the Pacific Ocean in the *Astrolabe* (1826–1829); they found five new species in the Strait of Gibraltar, shortly after the ship left Toulon [21], whilst the full zoological report of the ‘zoophytes’ discovered during the voyage (cnidarians and echinoderms) was published six years later [22]. The latter included three further new siphonophore species, from the Cape Verde Islands and from near Kangaroo Island off South Australia (*Praya dubia*), Bass Strait (*Bassia bassensis*) and off the northern coast of New Guinea (*Halistemma foliacea*).

During the latter half of the nineteenth century 36 more siphonophores were introduced (Figure 1). The decade between 1850 and 1860 saw 15 new species described, notably five by Huxley [23] in his important work the “*Oceanic Hydrozoa*”, and four by Keferstein and Ehlers [24], [25] from the Mediterranean. Huxley travelled to Port Jackson, the new British colony on the eastern coast of Australia (later Sydney), as assistant naturalist on board *HMS Rattlesnake* (1846–1850). He collected specimens of *Physalia* on the way out, and was the first to note that the body wall comprised two layers of cells, including nematocysts (the signature cells of cnidarians), and an intervening layer of mesogloea. Huxley was the consummate naturalist and a careful observer and illustrator of Siphonophora. He introduced two abylids (*Ceratocymba leuckarti* and *Abylopsis eschscholtzi*), the eudoxid bracts of the tropical diphyid *Eudoxoides mitra*, and anterior nectophores of *Diphyes chamissonis* (which lacks a posterior nectophore) from samples taken during these cruises. He also founded a new family the Sphaeronectidae based on three specimens of the small species *S. koellikeri* collected from the Indian Ocean, Torres Strait and east coast of Australia.

Two Germans, Carl Chun and Ernst Haeckel dominated the decade 1880–1890, adding five and seven new species of Siphonophora respectively (Figure 1). Haeckel wrote up the Siphonophora collected during the British *HMS Challenger* Expedition (1873–1876), with other specimens in a 380 page major work [26]. He founded a new family the Rhodaliidae (as an order, later abandoned) for three species with a large spherical pneumatophore, prominent gas gland and siphosome reduced to a corm, concluding that they were pelagic. Much later, in 1983, these siphonophores were shown by Pugh [27] to be benthic. Although Haeckel included 46 “new species” in his *Challenger* report, eight were chondrophores (now athecate hydroids, see below), and only four, in addition to the three rhodaliids, are now regarded as valid; these include two long-stemmed physonects and two prayid calycophorans (*Forskalia tholoides*, *Cordagalma ordinatum*, *Amphicaryon peltifera* and *Desmophyes annectens*). Overall, Haeckel’s treatment of the group was muddled. Indeed Totton (p. 6–13 [9]) wrote a critique of Haeckel’s classification and ill-founded Medusome Theory, whilst Mary Winsor (p. 322 [28] commented: “*Haeckel’s own description would lead us to expect that his* Challenger Report *on siphonophores was both a significant contribution to knowledge and a fine example of an evolutionist at work. Upon examination the picture is totally altered. The excitement of great ideas was well over by 1888, and the famous defender of Darwin seemed lacking in imaginative power. Instead of a case study of the clear impact of the Origin of Species upon a zoological problem, the siphonophores provide an example of the surprising success in interpreting animal relationships achieved by pre-Darwin-biologists.*” Despite this, many of Haeckel’s species descriptions and figures are still useful, provided account is taken of his short-comings. On the other hand, Carl Chun was more conservative and introduced one valid species from the Mediterranean (*Lensia subtilis*) in 1886 [29] and four more from the Canary Islands in 1888 [30]. He also added a further species in 1897 ([31] and see Figure 1), the diphyid calycophoran *Dimophyes arctica*.

In the twentieth century an average of ten new siphonophore species were introduced per decade, except during the pre-Second World War years (Figure 1). Specimens were collected either during expeditions or on routine (steam and sail) research cruises by British, American and other vessels. The Dutch *Siboga* Expedition (1899–1900) sampled the deep basins of the Indonesian Archipelago, and the 3,400 good siphonophore specimens collected were written up by Lens and van Riemsdijk [32]. These authors introduced nine new species including two new unusual calycophorans of unknown affinities, *Chuniphyes multidentata* and *Clausophyes galeata*. These were later placed by Totton [9] in a new mesopelagic diphyomorph family the Clausophyidae. The German Südpolar-Expedition to Antarctica (1901–1903) travelled in the Research Vessel *Gauss* to the ice edge in the Indian sector of the Southern Ocean and collected a large number of siphonophores. A sizeable report was produced by Fanny Moser, in which nine new species were introduced (together with two others described earlier). Her work was completed in 1914, but not published until after the First World War, in 1925 [33]. Her most notable new species was, perhaps, the richly colourful cold-water southern physonect *Pyrostephos vanhoeffeni* (p. 437–8 [33]). It is an abundant species in the Southern Ocean, and Moser placed it in a new family Pyrostephidae. The American *Albatross* Expeditions of the early 1900’s focussed on investigation of fish stocks and fish food under the leadership of Alexander Agassiz. The 1904–5 cruise investigated the relatively unknown area of the Pacific Ocean between South America and Easter Island, which proved to be very rich in pelagic life. Collection of the gelatinous zooplankton was supervised by Henry Bigelow, who produced a most comprehensive and well-illustrated report on siphonophores from the voyage [34]. Earlier the same year he published another paper on siphonophores from the Bay of Biscay [35], and together these two works included 11 siphonophore species new to science. The most notable are two benthic rhodaliids (*Dromalia alexandri* and *Stephalia dilata*), and several conspicuous pelagic calycophorans, including the large prayid *Praya reticulata*, and three species of a new and angular type of prayid referred by Bigelow to a new subfamily Nectopyramidinae. This group has been reviewed more recently by Phil Pugh ([13] and see below).

The most productive researcher on Siphonophora during the mid-twentieth century was A.K.Totton of the British Museum of Natural History (BMNH), London, England, who introduced 23 new species (Figure 1). He started work at the museum in 1914, aged 22, but almost immediately joined the army and fought in the First World War, where he was severely wounded and awarded the Military Cross [36]. By 1918 he was back in the museum in London, as Assistant Keeper and in charge of coelenterates. Over his lifetime he amassed an enormous collection of Siphonophora specimens which he used to write several important works. Much of his material came from the cruises of *RRS Discovery* ships run by the British Government from 1925 onwards, initially to Antarctica to study the biology of whales, but also, from 1929 onwards, to adjacent regions including the Indian and Pacific Oceans and Southern Atlantic Ocean. He also made annual spring visits from 1949 onwards to Station Zoologique, Villefranche, in the Mediterranean, where he was able to study live siphonophores in upwelled water for the first time, rear larvae and work out some of their life cycles. Totton also wrote important works on the Siphonophora of the Great Barrier Reef Expedition [37], of the Indian Ocean [38], and his most comprehensive systematic monograph, ‘Synopsis of the Siphonophora’ [9]. This last monograph covered all species he considered valid, but did not touch on their histology, physiology or distribution. In addition, Totton spent three months working on *Physalia physalis* in the Canaries with George Mackie in 1955, and produced the most detailed paper ever written on *Physalia* morphology [39]. The 23 new species he introduced over his lifetime (Figure 1) include 11 species of *Lensia*, a genus he erected in 1932. He also introduced two physonect genera (*Bargmannia*, *Marrus*), one new diphyid subfamily, the Sulculeolariinae, and one new diphymorph family, the Clausophyidae. As noted in his obituary [36], Totton had “*a sardonic humour, innate romanticism, warm personality and great* esprit”.

Significant contributions to new species introductions during the 20^th^ century were also made by Claude and Danielle Carré at the Station Zoologique, Villefranche-sur-Mer on the Mediterranean, S.D. Stepanjants from St Petersburg and R.Ya. Margulis from Moscow University. Claude Carré introduced four new species, including two prayine prayids and two valid species of the small-belled family Sphaeronectidae, all collected in the Bay of Villefranche. Between them the Carré’s wrote 29 papers on Siphonophora, as sole or joint authors, and some others with collaborators. They also reared live siphonophores, including *Muggiaea kochi* through several generations and at different temperatures [40]. In addition, Claude Carré wrote an important review of the diphyid subfamily Sulculeolariinae [41], showing that, for three species commonly found in the Mediterranean, both anterior and posterior nectophores were regenerated two or occasionally three times. Stepanjants introduced two new valid species from the NW Pacific *Apolemia vitiazi* and *Lensia asymmetrica* [1], while Margulis worked on the vast Russian collections of Siphonophora taken from all major oceans of the world over a period of three decades. She introduced five new species herein considered valid, mostly from subarctic or arctic seas, and one additional species she attributed to a new name, now reinterpreted as *Clausophyes moserae* [42]. In all Margulis wrote 29 papers on Siphonophora, many on their worldwide vertical and horizontal distribution.

The most prolific researcher of new siphonophore species since A.K. Totton has been Phil R. Pugh of the National Oceanography Centre, Southampton, UK. So far he has described 32 new species (Figure 1), many in collaboration with other researchers worldwide, and a number as sole author; more are “in preparation”. He took over study of the British National Collection from Totton in 1972, coincident with the launch of two *Johnson Sea-Link* manned submersibles from Harbor Branch Oceanographic Institution, Florida, USA in 1971 and 1975. Since then his research has gone from strength to strength. The American Johnson Sea Links (JSL I and II) provided him with much new and beautifully preserved material. Observers collect specimens using remotely controlled suction-operated canisters and other devices (reviewed in [43]). Fifteen new species taken by JSL I and II have been introduced by Pugh in papers published between 1987 and 2009, and another five species re-described. New species include physonects *Halistemma transliratum, Bargmannia amoena*, *Physophora gilmeri*, three species of *Forskalia* [16], and three more physonects with distinctive tentilla and muscle-free proximal surface to the nectosac for which Pugh has erected a new family Erennidae [15]; also five prayine prayid calycophorans [19], [44]. The American submersible *Alvin* collected a new benthic rhodaliid *Thermopalia taraxaca* (the Galapagos Dandelion) from the Galapagos Rift in 1979, one of 10 species re-assessed in an important work by Pugh [27] on the family Rhodaliidae. Then another rhodaliid, *Archangelopsis jagoa*, was collected by the German *JAGO* in the Gulf of Aqaba, and described by Hissmann, Schauer and Pugh [45]. Pugh also introduced five species from specimens collected by *Discovery* (1962), including a third rhodaliid, the physonect species *Bargmannia gigas* and three calycophorans (*Nectadamas richardi* and two species of *Clausophyes*). Two further species were collected from the Sargasso Sea using SCUBA; the prayine calycophoran *Rosacea flaccida* [46], and the physonect *Forskalia saccula* [16].

The most recent new siphonophores introduced by Pugh, some in collaboration with Casey Dunn (Brown, Rhode Island, USA) and Steve Haddock (MBARI, USA), were sampled by Remotely Operated Vehicles (ROV) in the northeast Pacific Ocean, off Southern California. They were mostly collected by the Monterey Bay Research Institute (MBARI) using the ROVs ‘*Tiburon*’ and ‘*Ventana*’. These new species include the physonect *Marrus claudanielis* (named for the Carrés), three physonects in a new genus *Resomia*, and five calycophorans. The resomiids have remarkable tentilla of two different types on the same tentacle, for which Pugh [17] created a new physonect family Resomiidae. Three of the calycophorans are new species in the family Sphaeronectidae [18]. Remarkable optical properties were discovered in the two new prayid species collected by ROV [7], see below. Pugh also collaborated with Francesc Pagès on Antarctic material collected by the German *RV Polarstern*, and together they discovered a remarkable new life stage in the clausophyid *Crystallophyes amygdalina*, the fuseudoxid [47].

Two distinctive pleustonic genera *Porpita* and *Velella* live on the ocean surface with the aid of a chitinous float. They were first introduced by Linnaeus [48], at the same time as *Physalia physalis*, and Eschscholtz [20] placed them in a family Velellidae, together with all other siphonophores then known. For a number of decades they were even thought of as ‘typical’ siphonophores, but studies on their larvae, beginning with Leloup [49] and Garstang [50] showed these to be more similar to actinulae of some Anthomedusae than to siphonula larvae of physonect siphonophores. This prompted Totton [38] to place them in a separate order Chondrophora. Behavioural and other studies by Mackie [51] on *Porpita* further demonstrated the anthomedusan affinities of chondrophores. These affinities were reiterated by Kirkpatrick and Pugh [52] who placed chondrophores in the Family Velellidae of the suborder Capitata, Order Athecata, in their ‘Synopsis of the British Fauna Series’. Later, Pagès et al. [53] referred them to the Family Porpitidae Goldfuss, 1818, and more recently Collins [54] sequenced the 18S gene (in 64 medusozoans) showing that chondrophores form a monophyletic clade within the Capitata, and are sister to the capitates *Millepora* and *Solanderia*. This has since been confirmed using 16S and 18S genes by Dunn et al. [10] and the 28S gene by Cartwright et al. [55]. Most recently, the Porpitidae are included, together with nine other families, in a clade Zancleida of the Suborder Capitata, Order Anthoathecata (fig. 5 [56]).

### Species Richness

**Figure 2:**
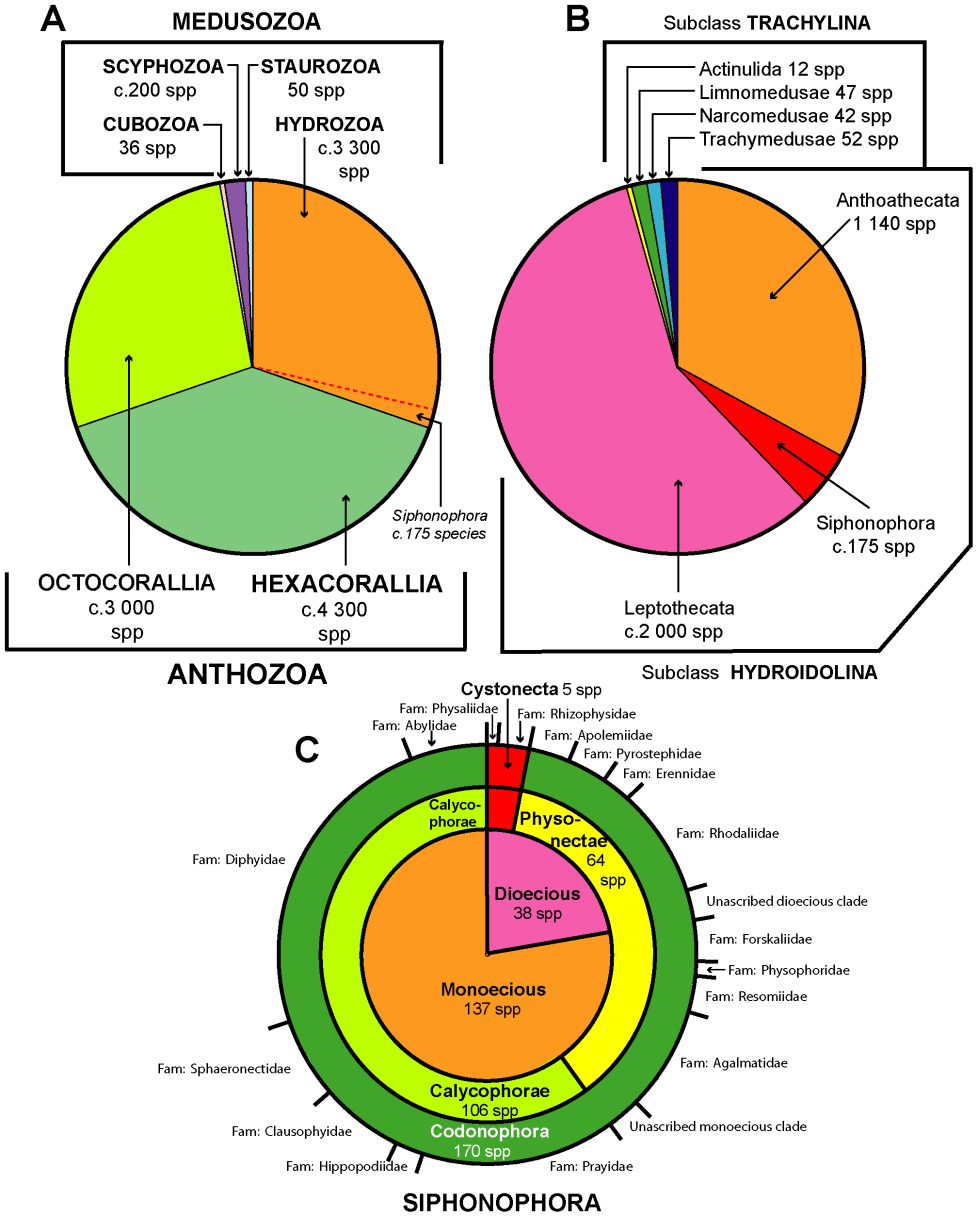
Cnidaria and Siphonophora Species Richness. A: the c. 11,000 Cnidaria species (excluding Myxozoa) subdivided into clades following Kayal et al. [58]; B: the c. 3,300 Hydrozoa species, subdivided into ranks from Daly et al. [59] and the present work; C: the 175 valid Siphonophora species subdivided into ranks based on Tables 3 and 4 of the present work.

Siphonophores are a small group within the large clade Hydrozoa of the phylum Cnidaria (Figure 2A), an ancient lineage currently thought to date back to the Pre-Cambrian late Cryogenian period, circa 640 million years ago [57]. A recent mitogenomic analysis of cnidarian mitochondrial genomes indicates that the oldest cnidarian clade may be the Anthozoa [58]. The clade Medusozoa is monophyletic [58], less speciose than the Anthozoa and comprises three relatively small clades Staurozoa (stalked jellyfish), Scyphozoa (true jellyfish) and Cubozoa (box jellyfish), and one much larger clade Hydrozoa [59].

Cnidae, or stinging cells (most of which are nematocysts), are a synapomorphy of Cnidaria. Nematocysts are discussed in relation to Siphonophora below. Anthozoans are exclusively polypoid and the recent mitogenomic analysis lends further support to the ‘polyp first’ hypothesis for cnidarian evolution [58]. Species of Medusozoa are defined by the presence of a medusa and a polyp stage in their life cycle, although in some the medusa stage has been secondarily lost, while in others the polyp stage has been lost. Medusozoa also have the unique apomorphic character of a linear mitochondrial genome [54]. Genes for the formation of cnidae are exclusive to cnidarians and found in no other metazoan for which the whole genome has been sequenced [60]. The parasitic clade Myxozoa may also be cnidarians, but further supporting evidence is needed and meanwhile they are excluded from Figure 2A.

Subdivisions of the Hydrozoa are illustrated in Figure 2B and comprise two monophyletic clades, Trachylina and Hydroidolina. The latter is the largest clade and includes Siphonophora and all the thecate and athecate hydroids, most of which have free-living planktonic medusa stages in their life cycles (Figure 2B). Trachylina is a small clade of four lineages, of which three contribute to the planktonic animal assemblage known as “hydromedusae”, the Limnomedusae, Narcomedusae and Trachymedusae.

Hydroidolina have lost the ecto-endodermal statocysts characteristic of other cnidarian taxa [61], yet exact relationships within the group remain uncertain [62]. It is clear from Figure 2B that the clades Anthoathecata and Leptothecata are more species-rich than Siphonophora. This can be related to the different life styles adopted by these groups, as well summarized by Gibbons et al. [63]. Species of Anthoathecata and Leptothecata are meroplanktonic (or meroplanktic), with a benthic ‘hydroid’ stage in addition to the pelagic medusa stage. Siphonophora species, however, are holoplanktonic (or holoplanktic), except for one family. They are not in any way tied to shallow continental shelf waters like anthoathecates and leptothecates. Instead, the distribution of Siphonophora extends throughout the ‘World Ocean’. Gibbons et al. [64], who studied patterns of hydrozoan species richness around South Africa, found a relatively large number of Siphonophora species compared to the number of other hydroidoline taxa, despite the relatively small ocean area sampled. In this respect, the relatively low species richness of Siphonophora is akin to that of the two trachyline groups Narcomedusae and Trachymedusae, which are also holoplanktonic [63]. Thus, Siphonophora are relatively species-poor compared to Anthoathecata and Leptothecata, with temperature and depth the main factors limiting their distribution. Siphonophora have had a long time to evolve into the variety of species and body forms seen in today’s seas, yet there is no fossil record. Angel [65] in his review of biodiversity in the pelagic ocean, quotes the controversial theory that such taxa may have become trapped in particular oceanic gyral centres (large rotating current systems) during evolution, some of which are believed to have persisted for 200 million years.

Species richness within the Siphonophora is shown in Figure 2C. The clade Cystonecta, which lack swimming bells, as noted above, contains only five species, while the sister clade Codonophora, or bell-bearers, includes all remaining 170 species. This latter clade comprises the two traditional groups Physonectae and Calycophorae, with physonects being a paraphyletic clade and calycophorans a monophyletic clade [10]. Currently, 175 species of Siphonophora are recognized as valid [1] and the majority are assigned to one of 16 families. However, 10 species of physonects remain currently unassigned, and are placed in one of two groups dependent on their sexual state: species either have separate sexes (dioecious) or bear both male and female sexual zooids on the same individual (monoecious), with zooids maturing at different times (Fig. 2C [1]). Sex has recently been shown to be an important character in the evolution of the Siphonophora, and is discussed further below. It is apparent from Fig. 2C that the most species-rich families of Siphonophora include the Rhodaliidae, the Agalmatidae, the Prayidae and the Diphyidae. The calycophoran families Sphaeronectidae, Clausophyidae and Abylidae also contain a relatively large number of species compared to other physonect families, confirming the success of the Calycophorae; this latter group includes the most abundant of all siphonophore species, *Chelophyes appendiculata* [2]. Species within each of the 16 codonophoran families are noted in the WoRMS Siphonophora World List [1].

#### Biogeography

Almost all siphonophores are deep sea pelagic organisms and the majority exhibit a cosmopolitan distribution; that is species present in all three great oceans and the Mediterranean. Siphonophore distribution was well covered in the last review [2], so is only summarized here for 44 selected siphonophore species (Tables 1 and 2).

**Table 1:** Distribution and abundance of selected cystonect, physonect, and prayomorph species.

Species	Habitat	Vertical range	Depth (m)	Latitudinal range	Abundance	Geographic range
*Physalia physalis*	pleustonic	surface	0	51°N-38°S	common	cosmopolitan
*Apolemia uvaria*	deep sea	epipelagic	0–100	60°N-38°N	rare	North Atlantic
*Bargmannia lata*	deep sea	deepermesopelagic	680–2500	48°N-33°S	rare	more atlower latitudes
*Pyrostephos vanhoeffeni*	deep sea	epi- andmesopelagic	75–1000+	41°S -71°S	common to rare	southern higherlatitudes
*Dromalia alexandri*	epibenthic	attached tosubstrate	300–600	36°N, 122°Wto 26°N, 113°W	locally common	off California only
*Rhodalia miranda*	epibenthic	attached tosubstrate	455–1098	37°S, 54°Wto 53°S, 59°W	locally uncommon	SW Atlantic only
*Marrus orthocanna*	deep sea	meso– andbathypelagic	50–3000	85°N-35°N	common	Arctic and sub-arctic
*Marrus antarcticus*	deep sea	meso- andbathypelagic	300–2100	43°S–67°S	uncommon	Antarctic and sub-antarctic
*Forskalia contorta*	deep sea	epipelagic	30–181	43°N-39°S	rare	cosmopolitan
*Resomia convoluta*	deep sea	meso- andbathypelagic	400–2800	60°S–68°S	very rare	Antarctic only
*Agalma elegans*	deep sea	epi- andmesopelagic	0–400	60°N-38°S	uncommon	cosmopolitan
*Nanomia bijuga*	neritic todeep sea	epi- andmesopelagic	0–800	55°N-59°S	very common	cosmopolitan
*Nanomia cara*	deep sea	epi- andmesopelagic	0–280	64°N-40°N	locally common	N Atlanticand Arctic only
*Physophora hydrostatica*	deep sea	epi- andmesopelagic	0–500	73°N-64°S	rare	cosmopolitan
*Amphicaryon acaule*	deep sea	epi- andmesopelagic	0–375	60°N-38°S	uncommon	cosmopolitan
*Praya dubia*	deep sea	epi- andmesopelagic	73–1000	59°N-40°S	rare	all seas exceptMediterranean
*Rosacea plicata*	deep sea	mainlymesopelagic	200–1610	62°N-59°S	uncommon	cosmopolitan
*Nectadamas* *diomedeae*	deep sea	meso- andbathypelagic	260–2500	81°N-59°S	rare	cosmopolitan
*Nectopyramis thetis*	deep sea	mainlymesopelagic	200–1500	63°N-34°S	rare	cosmopolitan
*Hippopodius* *hippopus*	deep sea	epipelagic	0–300	50°N-38°S	common	cosmopolitan
*Vogtia serrata*	deep sea	mesopelagic	200–1000+	62°N-71°S	uncommon	cosmopolitan

Key: epipelagic, 0- ca. 300 m; mesopelagic, 300–1000 m; bathypelagic, 1000 m and below. Abundance scale: very common, common, uncommon, rare, very rare. Cosmopolitan refers to species present in all three great oceans and the Mediterranean. For primary literature used to construct this table, see [1].

**Table 2:** Distribution and abundance of selected diphyomorph species.

Species	Habitat	Vertical range	Depth (m)	Latitudinal range	Abundance	Geographic range
*Chuniphyes* *multidentata*	deep sea	meso– andbathypelagic	300–2500	63°N-59°S	uncommon	cosmopolitan
*Clausophyes* *moserae*	deep sea	meso- andbathypelagic	500–2114	60°N-67°S	uncommon	cosmopolitan
*Kephyes ovata*	deep sea	epi– andmesopelagic	78–1000	60°N-70°S	uncommon	cosmopolitan
*Crystallophyes* *amygdalina*	deep sea	epi- andbathypelagic	380–2000+	51–81°N& 33–74°S	uncommon	bipolar
*Heteropyramis* *crystallina*	deep sea	meso- andbathypelagic	300–2600	62°N-71°S	rare	cosmopolitan
*Sulculeolaria* *biloba*	deep sea	epipelagic	0–200	62°N-38°S	uncommon	cosmopolitan
*Sulculeolaria* *quadrivalvis*	deep sea	epipelagic	0–300	52°N-38°S	uncommon	cosmopolitan
*Chelophyes* *appendiculata*	deep sea	epipelagic	0–500	46°N-38°S	very common	cosmopolitan
*Dimophyes arctica*	deep sea	epi- andmesopelagic	0–600	81°N-71°S	common	cosmopolitan
*Diphyes dispar*	deep sea	epipelagic	0–300+	43°N-42°S	common	cosmopolitan
*Eudoxoides mitra*	deep sea	epipelagic	0–200+	36°N-38°S	common	all seas exceptMediterranean
*Lensia conoidea*	deep sea	epi- andmesopelagic	0–600+	63°’N-46°S	common	cosmopolitan
*Lensia fowleri*	deep sea	epipelagic	0–200+	61°N-33°S	rare	cosmopolitan
*Lensia* *multicristata*	deep sea	epi- andmesopelagic	200–500+	54°N -54°S	common	cosmopolitan
*Muggiaea* *atlantica*	neritic	epipelagic	0–100+	59°N-53°’S	common	almost alloceans
*Muggiaea kochi*	neritic	epipelagic	0–100+	59°N-38°S	locally common	Atlantic andMediterranean
*Muggiaea* *bargmannae*	neritic to deep sea	meso- andbathypelagic	400–2000+	36–87°N& 43–71°S	uncommon	bipolar
*Gilia reticulata*	deep sea	meso– andbathypelagic	500–2000	73°N-71°S	rare	cosmopolitan
*Abyla trigona*	deep sea	epipelagic	0–200	37°N-33°S	rare	all seas exceptMediterranean
*Ceratocymba* *sagittata*	deep sea	epipelagic	0–200	59°N-44°S	common	all seas exceptMediterranean
*Abylopsis* *tetragona*	deep sea	epipelagic	0–200+	57°N-47°S	common	cosmopolitan
*Bassia* *bassensis*	deep sea	epipelagic	0–200	60°N-41°S	common	cosmopolitan
*Enneagonum* *hyalinum*	deep sea	epipelagic	0–200+	58°N-40°S	uncommon	cosmopolitan

Key: epipelagic, 0- ca. 300 m; mesopelagic, 300–1000 m; bathypelagic, 1000 m and below. Abundance scale: very common, common, uncommon, rare, very rare. Cosmopolitan refers to species present in all three great oceans and the Mediterranean. For primary literature used to construct this table, see [1].

The majority of siphonophores are deemed cosmopolitan in this paper if their geographical ranges encircle the globe within their preferred latitudinal bands. Such bands are dependent on both water temperature and ocean currents. Warm water siphonophores such as *Forskalia contorta* and *Hippopodius hippopus* (in Table 1), as well as *Sulculeolaria biloba*, *S. quadrivalvis*, *Diphyes dispar*, *Eudoxoides mitra* and the abylids *Abyla trigona*, *Ceratocymba sagittata*, *Abylopsis tetragona*, *Bassia bassensis* and *Enneagonum hyalinum* (in Table 2) mostly inhabit shallow epipelagic layers at tropical latitudes. Other species such as *Agalma elegans*, *Physophora hydrostatica*, *Vogtia serrata* (in Table 1), the clausophyids *Chuniphyes multidentata*, *Clausophyes moserae*, *Kephyes ovata*, *Heteropyramis crystallina* and the diphyids *Lensia conoidea*, *L. multicristata* and *Gilia reticulata* (in Table 2) occupy a broader latitudinal range in either epipelagic layers at higher latitudes or deeper mesopelagic layers at lower latitudes. A few species are restricted to deep horizons throughout their ranges (eg *Bargmannia lata*, *Resomia convoluta*, *Nectadamas diomedeae* and *Nectopyramis thetis*), others are bipolar (*Crystallophyes amygdalina*, *Muggiaea bargmannae*) or restricted to just one polar region (*Marrus orthocanna*, *M. antarcticus*). A number of oceanic species do not occur in the Mediterranean (Tables 1, 2). A few species are neritic (for example *Muggiaea* species, Table 2), and species of the physonect family Rhodaliidae (*Dromalia alexandri* and *Rhodalia miranda* Table 1) are epibenthic, found only in specific areas of the continental shelf surrounding the major continents [27]. One species, *Dimophyes arctica* (Table 2) inhabits all latitudes.

Species from the neritic calycophoran family Sphaeronectidae are omitted because a recent review [18] indicates that most species of *Sphaeronectes* have been incorrectly identified in the past. Other records of certain species noted by particular authors are also omitted due to suspect identifications. This problem and others associated with estimating the worldwide distribution of siphonophores was reviewed recently by Mapstone [6, section 5.2], to which the reader is referred for further information. Primary data used to construct Tables 1 and 2 is available from the WoRMS Siphonophora List [1], and updated as new reliable records become available.

### Body Plans and General Morphology

**Figure 3:**
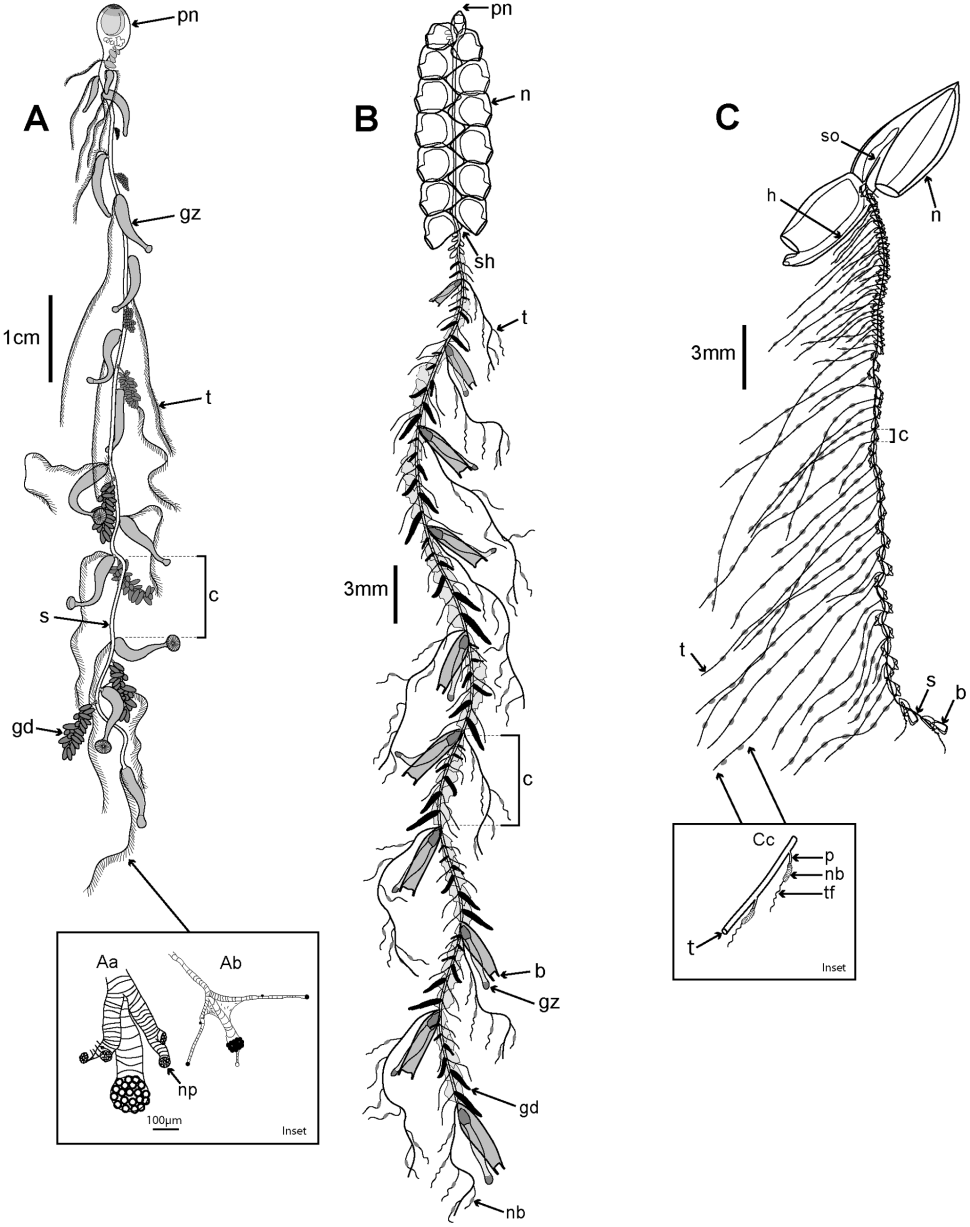
Three typical siphonophore body plans. A. Long-stemmed cystonect *Rhizophysa eysenhardti* (derived from [66] pl. 14 fig. 1): inset shows nematocyst pads on two interpretations of tricornuate tentacular side branches from *Rhizophysa filiformis*, (Aa: derived from [67] fig. 5 and Ab: derived from [9] pl. 4, fig. 2): B. Long-stemmed physonect *Nanomia bijuga* (derived from [68], pl. 7, fig. 1); C. Typical calycophoran *Lensia conoidea* (derived from photo image by Rob Sherlock - shown in Fig. 5C): inset Cc shows two tentilla attached to one tentacle (derived from [69] pl. 11, fig. 2). Labels: b - bract; c – cormidium; gd - gonodendron; gz - gastrozooid; h – hydroecium; n – nectophore (swimming bell); nb – nematocyst battery (a coiled cnidoband); np – nematocyst pad; p - pedicel; pn – pneumatophore (float); s – stem; sh – siphosomal horn; so – somatocyst; t – tentacle; tf – terminal filament.

Siphonophores vary greatly in size and shape, and are polymorphic individuals composed of a number of polypoid and medusoid zooids which together function as an integrated organism. Most siphonophores conform to one of three body plans, representing the three main types Cystonecta, Physonectae and Calycophorae (Figure 3A–C). A typical long-stemmed cystonect (Figure 3A *Rhizophysa eysenhardti*) has a pneumatophore (float) at the anterior end, followed by an elongate stem bearing groups of iterative (repeated) zooids specialized for different functions. The stem and zooid groups are collectively termed the siphosome, and each zooid group (in the cystonect species shown in Fig. 3A) comprises a gastrozooid with tentacle (for capture, ingestion and digestion of prey items) and a gonodendron bearing several gonophores for reproduction (of one sex only in each individual). Long-stemmed cystonects lack prominent swimming bells and instead, in a calm sea, may drift peacefully at the surface and writhe by contracting the stem muscles [9]. A typical long-stemmed physonect (Figure 3B *Nanomia bijuga*), in contrast, has a pneumatophore and an extra portion of stem interpolated between the pneumatophore and siphosome, termed the nectosome, which bears many nectophores (swimming bells). The nectophores contract in a co-ordinated pumping manner and direct water backwards, moving the animal forward by “jet propulsion”. In *Nanomia bijuga* iterative units are spread out along the siphosomal stem, as in a long-stemmed cystonects, and are termed cormidia because each originates from a single probud (as noted above). A cormidium of *N. bijuga* comprises a gastrozooid with tentacle (branched in most physonects), several smaller palpons, each with a palpacle, gelatinous bracts of two sizes (for extra buoyancy), and gonodendra (with gonophores of both sexes in each individual). A typical calycophoran (Figure 3C, *Lensia conoidea*) has two nectophores but no pneumatophore, and an elongate siphosomal stem with many closely spaced and reduced cormidia, each comprising a gastrozooid with a prominent elongate tentacle, one bract and gonophores; the latter start to develop while the cormidium is still attached to the stem, and at maturity the cormidium detaches from the end of the stem to become a free-living eudoxid.

**Figure 4:**
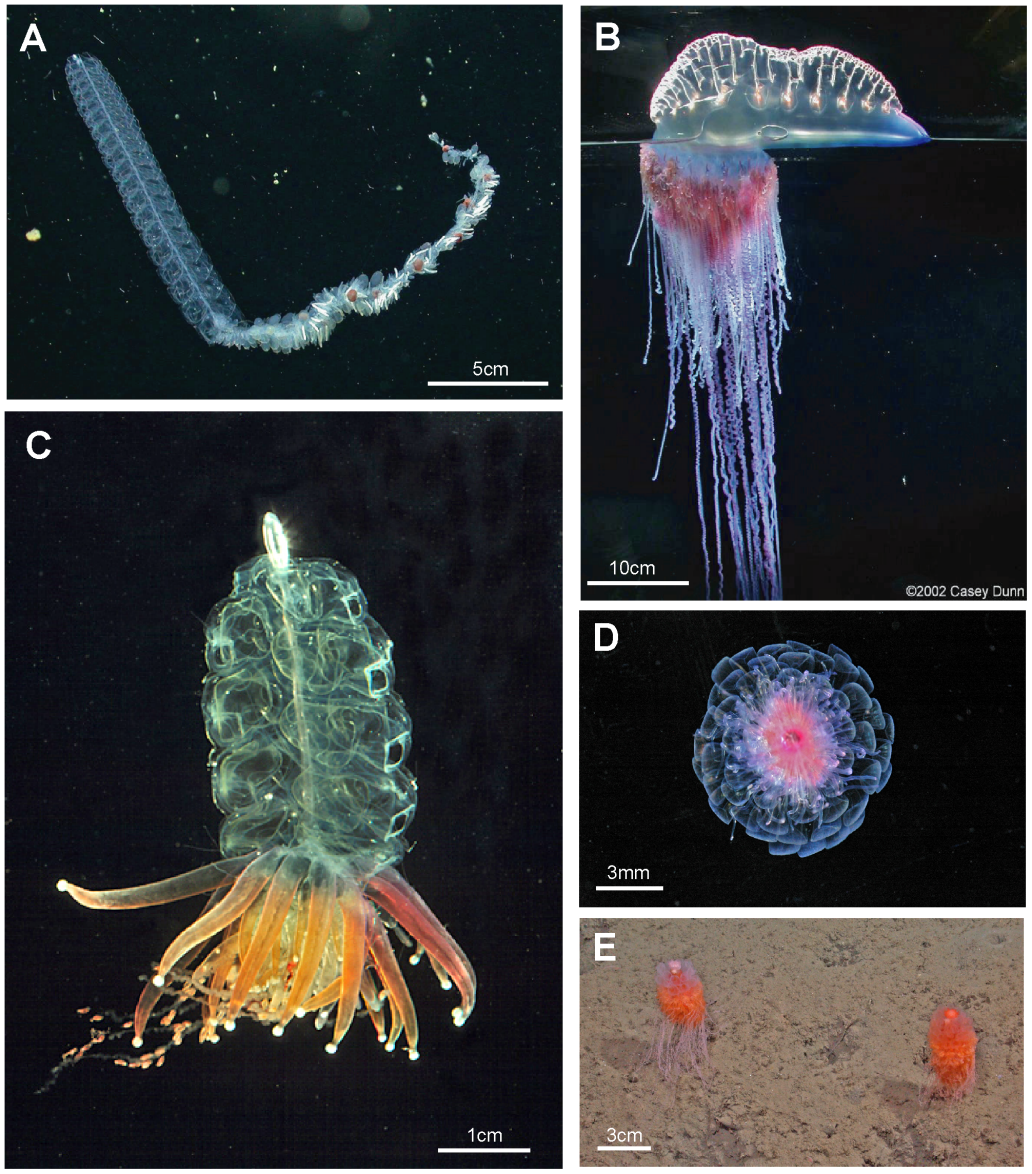
Cystonects and physonects. A. Typical long-stemmed physonect *Bargmannia* sp., with small anterior pneumatophore, many nectophores on an elongate nectosome and iterative cormidia on an elongate siphosome (MBARI); B. Atypical cystonect *Physalia physalis*, pleustonic (lives at surface), with much enlarged pneumatophore, no stem, cormidia arising directly from underside of pneumatophore (Casey Dunn © 2002); C. Atypical physonect *Physophora hydrostatica,* with pneumatophore, nectophores on an elongate nectosome and cormidia on a short-stemmed corm-like siphosome (Larry Madin © WHOI); D. Atypical physonect *Athorybia rosacea*, with rose-pink pneumatophore surrounded by rings of large bracts from cormidia on short-stemmed corm-like siphosome; no nectosome (Larry Madin © WHOI); E. Atypical physonect *Dromalia alexandri,* with enlarged penumatophore, ring of nectophores on short nectosome and whorls of iterative cormidia spiralling around corm from growth zone to corm base on short-stemmed siphosome (MBARI). Scale bars approximate.

A range of typical and atypical cystonect and physonect body plans are shown in Figure 4. *Bargmannia* is a typical long-stemmed physonect (Figure 4A), and is larger than the *Nanomia bijuga* colony shown in Figure 3B; the specimen photographed has possibly lost some of its siphosome. The cystonect *Physalia physalis* (Figure 4B) is unusual and differs from the more usual cystonects colony shown in Figure 3A because the former has a much larger pneumatophore, which lies on the sea surface, and no stem. Cormidial siphosomal zooids in *P. physalis* hang down directly from the underside of the pneumatophore at the ‘oral’, or posterior, end. The physonect *Physophora hydrostatica* (Figure 4C) is also somewhat atypical with an intermediate-sized pneumatophore and typical nectophores on an elongate nectosome, but the siphosome is reduced to a swollen corm and surrounded by a ring of prominent enlarged palpons. The physonect *Athorybia rosacea* has an even more reduced stem (Figure 4D), comprising only a swollen red-tipped pneumatophore and adjacent siphosomal protuberance where enlarged bracts form; these encircle the pneumatophore in rings, and are capable of limited ‘paddling’ locomotion [38]. Rhodaliids are another unusual family of shortened siphonophores, which, unlike most other families are epibenthic, so live attached to the bottom by their long tentacles. In the rhodaliid *Dromalia alexandri* (Figure 4E), the pneumatophore is relatively large compared to that of a typical long-stemmed physonect and gives sufficient lift to maintain the animal just above the sea bed [4]; it can also use the ring of small weak nectophores to swim short distances.

**Figure 5:**
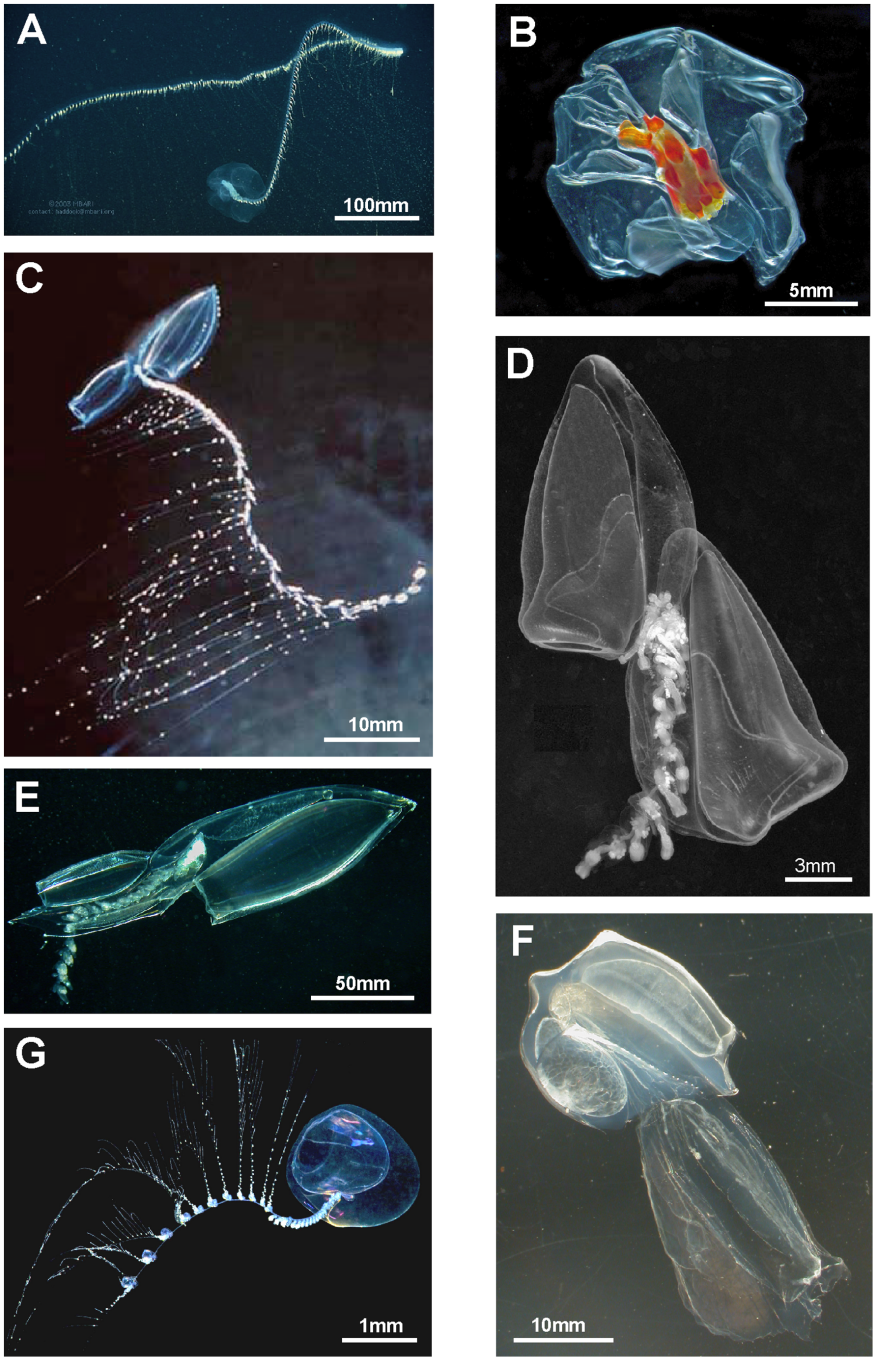
Calycophorans. A. Typical prayomorph *Praya* sp., with two rounded bells and a very long siphosome bearing over 100 cormidia; tentacles are extended for feeding, each bearing 80–90 nematocyst batteries, giving <9000+ batteries in all (Steven Haddock © MBARI); B. Atypical prayomorph *Hippopodius hippopus* with several facetted nectophores enclosing central chamber; latter contains short stem with cormidia which lack bracts to facilitate complete stem withdrawal (Russ Hopcroft, UAF); C. Typical diphyid diphyomorph *Lensia conoidea* with two angular linearly aligned bells; stem extended for feeding and with many closely spaced cormidia; each has an elongate tentacle with 15+ tentilla (better shown in Figure 3C) for feeding (Rob Sherlock, MBARI); D. Typical clausophyid diphyomorph *Kephyes ovata* with two staggered bells and a partly contracted stem bearing cormidia with bracts (MBA); E. Another typical diphyid diphyomorph *Chelophyes appendiculata,* with stem partly withdrawn into hydroecium of posterior (smaller) nectophore (P. Schuchert, MHNG); F. Typical abylid diphyomorph *Abyla trigona,* with two linearly aligned facetted bells and stem withdrawn into hydroecium of posterior bell (P.R. Pugh, with permission) G. Typical sphaeronectid diphyomorph *Sphaeronectes pagesi*, with a single bell (representing larval nectophore retained) and stem with tentacles (with tentilla) extended for feeding (D. Lindsay, R. Minemizu, JAMSTEC).

A range of calycophoran body plans are shown in Figure 5 and two main types are distinguished: prayomorphs, with a pair of rounded and opposed swimming bells and an extended siphosome (Figure 5A) and diphyomorphs with a pair of more streamlined bells attached in a linear arrangement one behind the other (Figure 5C). The siphosomal stem of diphyomorphs can be completely withdrawn into the hydroecium for greater protection (Figure 5E). Unusual calycophoran body plans include hippopodiids with several typically facetted swimming bells arising on pedicels one from another, which enclose a cavity into which the stem can be completely withdrawn (Figure 5B); and sphaeronectids in which a single rounded larval swimming bell is retained throughout life (Figure 5G). Swimming bells of tropical abylid diphyomorphs are also arranged linearly (Figure 5F) and their surfaces are also facetted, whereas clausophyid diphyomorphs typically have two staggered bells (Figure 5D) in an arrangement intermediate between the apposed bells of prayomorphs and the linearly aligned bells of diphyomorphs.

The pneumatophore (float) is unique to siphonophores, and a ‘neoformation’ (p. 103 [2]; p. 125 [70]), not a modified medusoid zooid as originally concluded by certain nineteenth century authors [26]. Embryological work by Danielle Carré [71], [72], [73] demonstrated pneumatophore formation in three physonect species. Each pneumatophore comprises a gas gland (pneumadenia) and a central chitin-lined gas chamber (pneumatosaccus), with a second cavity (the pericystic cavity) typically subdivided by septa which surrounds the gas cavity and is confluent with the gastrovascular cavity of the main stem. Carbon monoxide is secreted into the gas cavity by the gas gland and the pneumatophore acts as a hydrostatic organ (reviewed by Mackie et al. (p. 194–196 [2]). For example, as the physonect *Nanomia bijuga* rises in the water column, bubbles of expanding gas are released via an apical pore surrounded by a sphincter muscle [74]. The importance of the pneumatophore for buoyancy varies in different species. In cystonects it is the only structure providing lift for the heavy stem and attached zooids. In physonects the pneumatophore is small, whilst bracts are present that increase buoyancy by the replacement of 44% of the heavy sulphate ions in the mesogloea by lighter chloride ions. Calycophorans lack a pneumatophore, and up to 75% of the sulphate ions in each bract are replaced to provide buoyancy [75].

Nectophores are asexual medusoid structures that contain a muscular nectosac opening via an ostium. Strong contraction of this nectosac forces water out of the bell and propels the siphonophore forwards, or in some cases the ostia are directed forwards to achieve backward swimming [76]. During swimming the stem of physonects shortens to improve streamlining. In many calycophorans streamlining is taken a stage further by contraction of the stem into an additional external hollowed out chamber known as the hydroecium (as noted above). In addition, many calycophoran nectophores contain an extra structure in the mesogloea adjacent to the nectosac termed the somatocyst; this often contains oil globules which can both act as a food store and give extra lift.

The siphosomal stem of a siphonophore can extend from a few centimetres in small diphyid calycophorans (Figures 5C, 5G) to several metres in larger physonects and prayid calycophorans. (Figure 5A) Cormidia are replicated many times along the stem, as noted above, and each typically contains the following zooids: a gastrozooid with single tentacle for feeding, one or more gonophores (borne on tree-like gonodendra in cystonects and most physonects) for reproduction, and one or several bracts for buoyancy and protection (bracts are absent in cystonects). Tentacles have side branches in most siphonophores, bearing either ‘pads’ of nematocysts (cystonects, Figure 3A inset) or complex nematocyst batteries (physonects and calycophorans, Figure 3C inset) here termed ‘tentilla’. Physonect cormidia also contain one or more reduced gastrozooids called palpons, which have a chemosensory or excretory function (Figure 3B); each palpon bears a reduced tentacle, the palpacle.

Cormidia can be pedunculate (attached at one point on the siphosome), as in calycophorans (Figure 3C) or dispersed along the length of the siphosome, as in long-stemmed cystonects and physonects (Figure 3A–B). In many calycophorans, mature cormidia detach as they reach the end of the stem to become free-living eudoxids, (the sexual stage in the life cycle) in the plankton. In other calycophorans cormidia are retained on the stem throughout life. Free-living eudoxids comprise a single bract (conical buoyant zooid) covering a gastrozooid with tentacle and a gonophore (see below). More gonophores form after the first is released and production may continue for several months.

**Figure 6:**
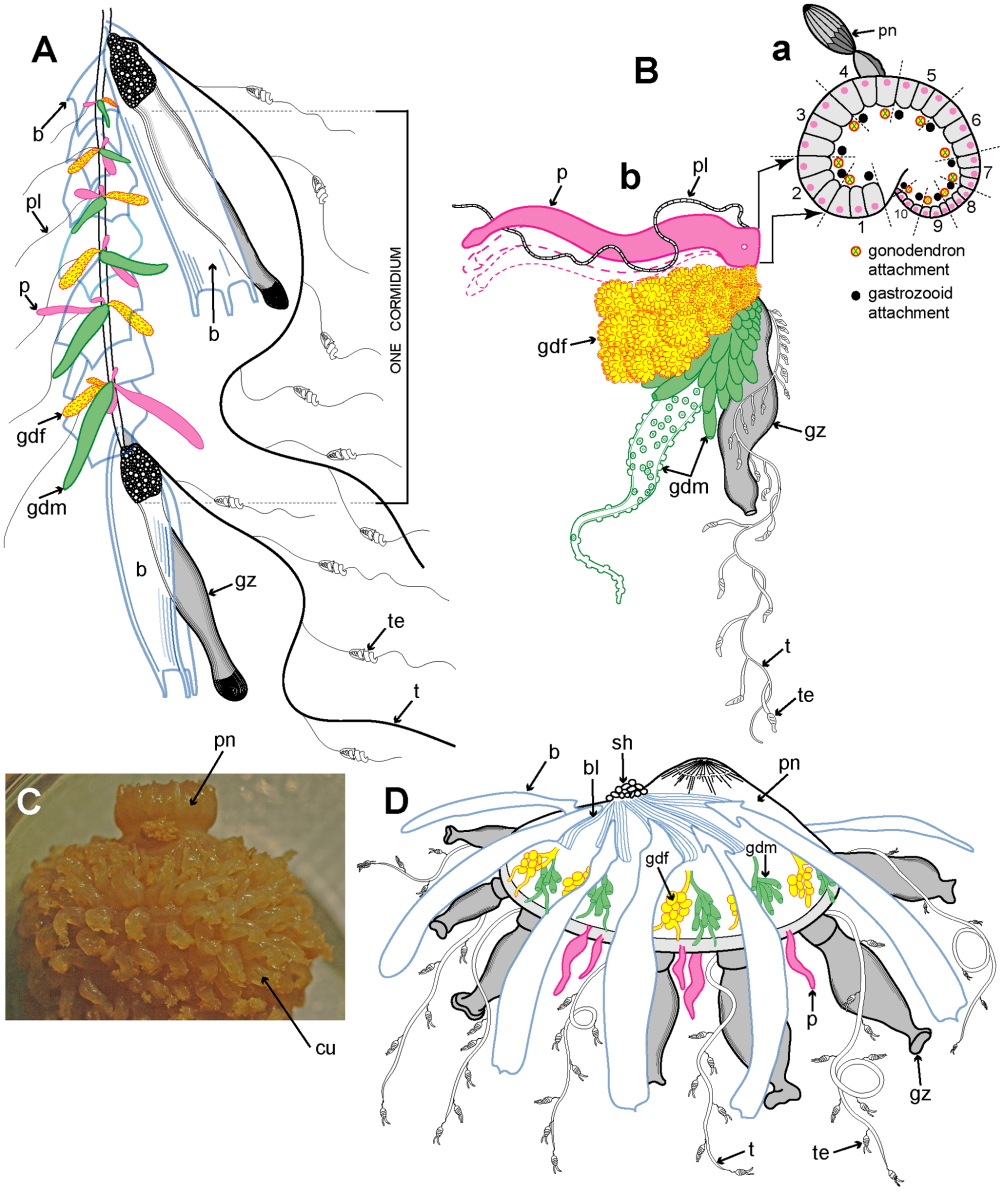
Physonect cormidia. A: *Nanomia bijuga* cormidium (derived from [68] pl. 7, fig. 10); B: *Physophora hydrostatica* a. diagram of posterior view of corm surface bearing 10 cormidia (derived from [77] figs. 12a, 16a); b. one cormidium exploded (derived from [26] pl. 20, fig. 18 with two additional palpons added); C: *Dromalia alexandri* dorsal view of corm with many spirally arranged cormidial units, dorsal view (GMM); D: *Athorybia rosacea* lateral view of float with siphosomal horn and attached cormidia (derived from [50] txt fig. 45). Labels: b – bract; bl – bracteal lamella; cu – cormidial unit; gdf – female gonodendron; gdm – male gonodendron; gz – gastrozooid; p – palpon; pl – palpacle; pn – pneumatophore (float); sh – siphosomal horn; t – tentacle with tentilla; te - tentillum.

Example cormidia from a range of physonects are shown in Figure 6, covering typical long-stemmed as well as short-stemmed types. A cormidium from the typical long-stemmed physonect *Nanomia bijuga* comprises several palpon-gonodendra-bract complexes and large posterior gastrozooid with an associated elongate bract (Figure 6A). The palpon complexes become progressively older and larger posteriorly, and all elements of each cormidium originally arose from a pro-bud (as noted earlier) on the siphosomal horn at the anterior end of the siphosome [8] (Figure 3B). One of 10 cormidia from the physonect *Physophora hydrostatica* occupies a compact segment of the siphosomal corm, and includes three enlarged lateral palpons, an associated hermaphrodite gonodendron of male and female gonophores, with a gastrozooid and tentacle on the posterior surface, but no bracts (Figure 6B a–b). In the rhodaliid *Dromalia alexandri* (Figure 6C) cormidia are borne on branched cormidial units away from the corm surface, and these units spiral around the inflated corm to the posterior under-surface [4]. Cormidial units originate continuously on a siphosomal horn between the nectophores (swimming bells), on the ventral surface just below the pneumatophore, and each mature unit typically carries three cormidia. A single cormidium includes a gastrozooid, several palpons and many gonophores in a gonodendron [4]. Dendritic growth of the cormidial units enables a large number of cormidia to be carried on a single rotund *D. alexandri* individual. Cormidia on the enlarged float of *Athorybia rosacea* (Figure 6D) originate from a siphosomal horn adjacent to the float apex, and each includes a group of typically four large larval bracts, an associated branched hermaphrodite gonodendron with small palpons below, and a larger gastrozooid on the posterior corm surface.

**Figure 7:**
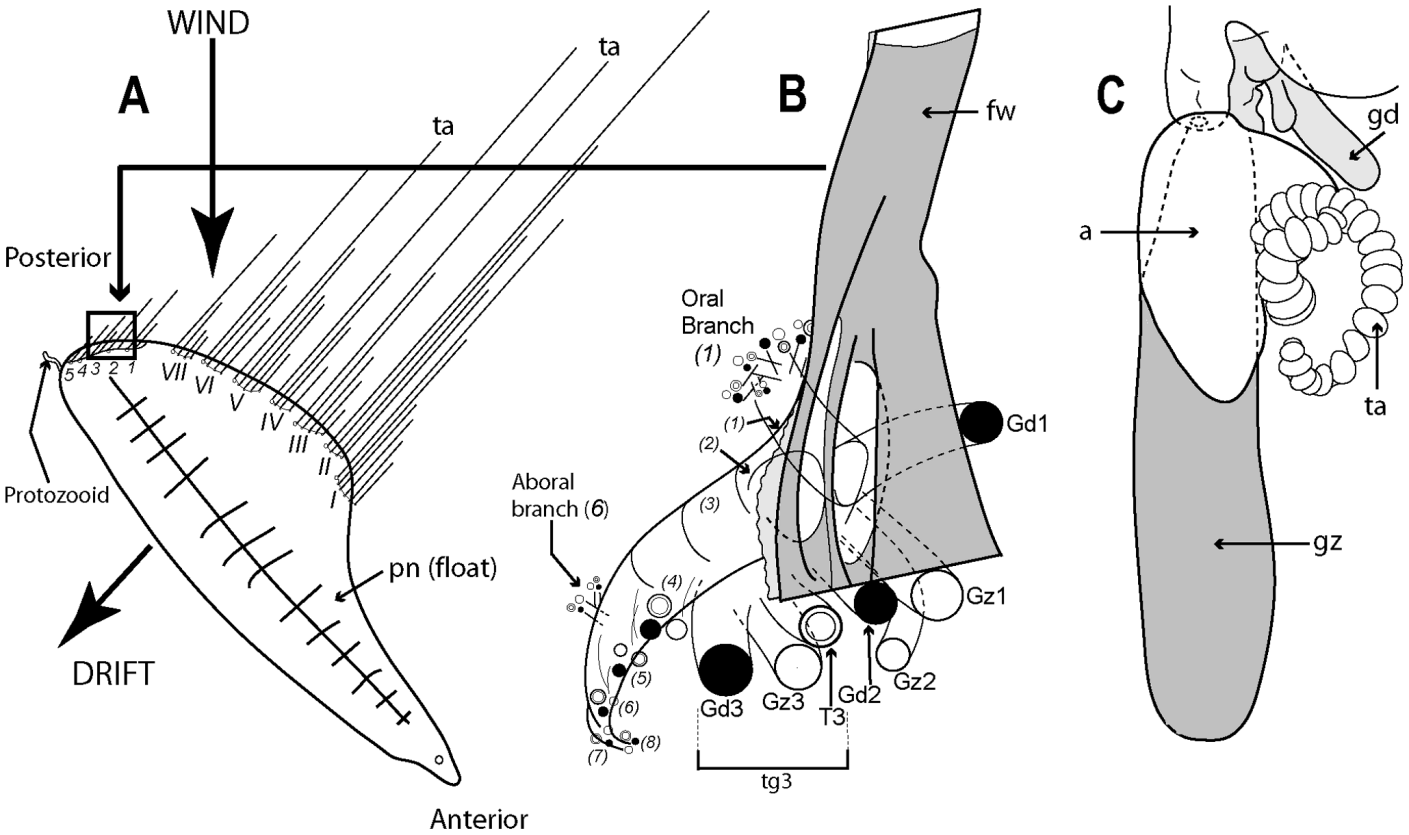
Cystonect cormidia as exhibited by *Physalia physalis*. A: Left-handed drifting specimen viewed from above (derived with minor modification from [39] fig. 5) – added numbers 1–5 identify oral cormidial groups while numbers I–VI identify main cormidial groups – note how *Physalia’*s surface float drifts to starboard with the wind on a broad reach; B: Oral cormidial complex number 2 viewed from inside the float – note groups 3 to 8 are tripartite, with more tripartite groups on oral and aboral side branches (adapted from [39] txt fig. 12D) – numbers in brackets added to identify tripartite groups; C: A developing tripartite group from main cormidial complex number VI (derived from [39] txt fig. 14B, in part only). Labels: a – ampulla (basigaster); fw – float wall; gd – gonodendron; gz – gastrozooid; pn – pneumatophore (float); ta – tentacle with ampulla (basigaster); T – tentacle; tg – tripartite group.

Figure 7A illustrates the complexity of a mature Portuguese Man O’War *Physalia physalis* viewed from above and ‘sailing’ with the wind, with many long tentacles extending from the cormidia and streaming out from the windward side. The cormidia of *P. physalis* are shown diagrammatically in Figure 7A, and numbered 1–5 and I –VII; they originate directly from the underside of the float (pneumatophore) in this species and develop in a particularly complex pattern, as described and illustrated in a seminal paper by A.K. Totton [39]. Cormidia bud one from another in a series, and each such series is termed a cormidial complex. There are twelve cormidial complexes in a mature *P. physalia*, which are attached in two groups separated by a small gap; the oldest complex in each group, (which forms first) lies closest to the anterior (or aboral) end of the animal (Figure 7A). The smaller oral group of complexes (1–5) lies just posterior of the first gastrozooid to form in the larva, the protozooid, and one cormidial complex from this region is shown in Figure 7B. It bears c. 13 cormidia, on two branches: a smaller oral branch above which is directed towards the oral end of the float, and a larger aboral branch below which is directed towards the aboral, or posterior, end of the animal. Almost all the cormidia of *P. physalis* comprise three zooids: a gastrozooid, gonodendron and a separate tentacle with ampulla (where the nematocysts are formed), which together form a tripartite group (Figure 7C). As growth proceeds more tripartite groups develop on lateral branches from the cormidial complex, filling every available space (Figure 7B). Indeed, no other siphonophore buds so prolifically as *P. physalis* [39]. As sexual maturity is reached, the gonodendra of each cormidial complex sub-branch many times, and detach. The largest such gonodendral ‘sphere’ found by Totton (from a female) measured <5 cm across, and bore 2400 gonophores on seven main branches, plus 224 very small medusoid bells, an extra zooid present in the cormidial complexes of mature *P. physalis*.

**Figure 8:**
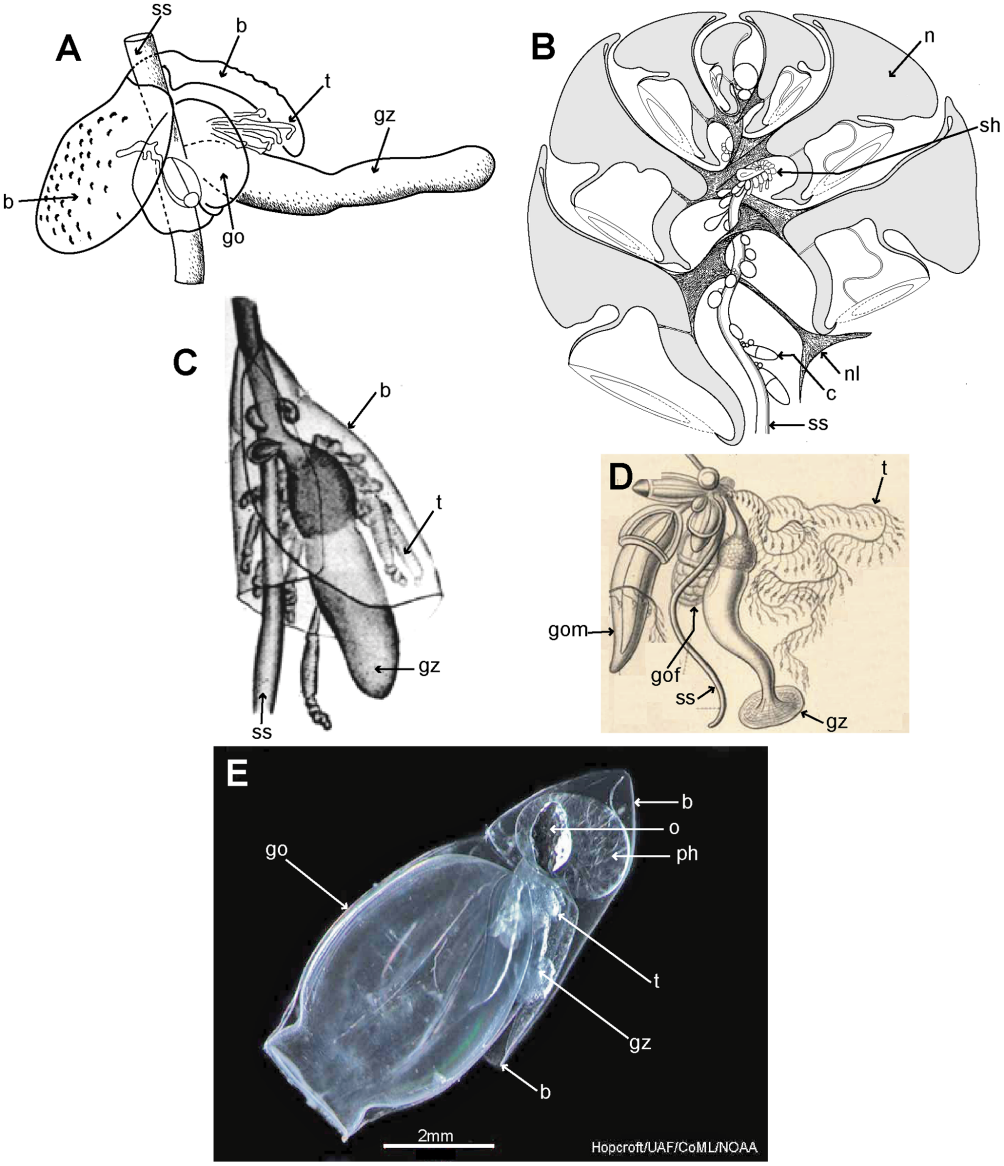
Calycophoran cormidia. A: *Rosacea cymbiformis* cormidium (after [6] fig. 2D); B. *Hippopodius hippopus* section through colony (adapted from [31] fig. 11, [78] txt fig. 13 and [27] fig. 44b); C: *Chelophyes appendiculata* cormidium (from [34] pl. 11, fig. 1); D. *Hippopodius hippopus* cormidium; note, no bracts (from [26] pl. 29, fig. 1 in part); E. *Dimophyes arctica* eudoxid (Russ Hopcroft, UAF). Labels: b – bract, c – cormidium; go – gonophore; gof – female gonophore; gom – male gonophore; gz – gastrozooid; n – nectophore; nl – nectophoral lamella; o – oil globule (in phyllocyst); ph – phyllocyst; sh – siphosomal horn; ss – siphosomal stem; t – tentacle with tentilla.

Cormidia are discrete in calycophorans, and, with one exception, lack the palpons present in physonect cormidia. In many calycophoran cormidia, the bract wraps around the stem in a cloak-like manner and gives protection to the underlying gastrozooid and gonophores (Figure 8A, C). As already noted above, when the cormidium of most diphyomorphs reaches maturity, it detaches and becomes a free-living eudoxid (Figure 8E). In some calycophorans, however, cormidia remain attached to the stem throughout life (prayine prayids and sulculeolariine diphyids). A few groups lack bracts, including members of the prayomorph family Hippopodiidae (see above), and *Clausophyes* species of the diphyomorph family Clausophyidae, both of which also probably retain their cormidia on the stem. In hippopodiids, a number of bells remain joined together when mature, forming a hollow cylinder from which the siphosomal stem emerges at the posterior end (Figure 8B). This stem originates between the two youngest nectophores but only the bottom two bells are functional in hippopodiids; their mesogloea, together with that of the other smaller bells, give buoyancy to compensate for the absence of bracts in the cormidia (Figure 8D). Cormidia arise from a siphosomal horn and are small, allowing the stem to be completely withdrawn into the cylindrical chamber when not feeding, as already noted above (Figure 8B).

### Old and New Phylogenies

**Figure 9:**
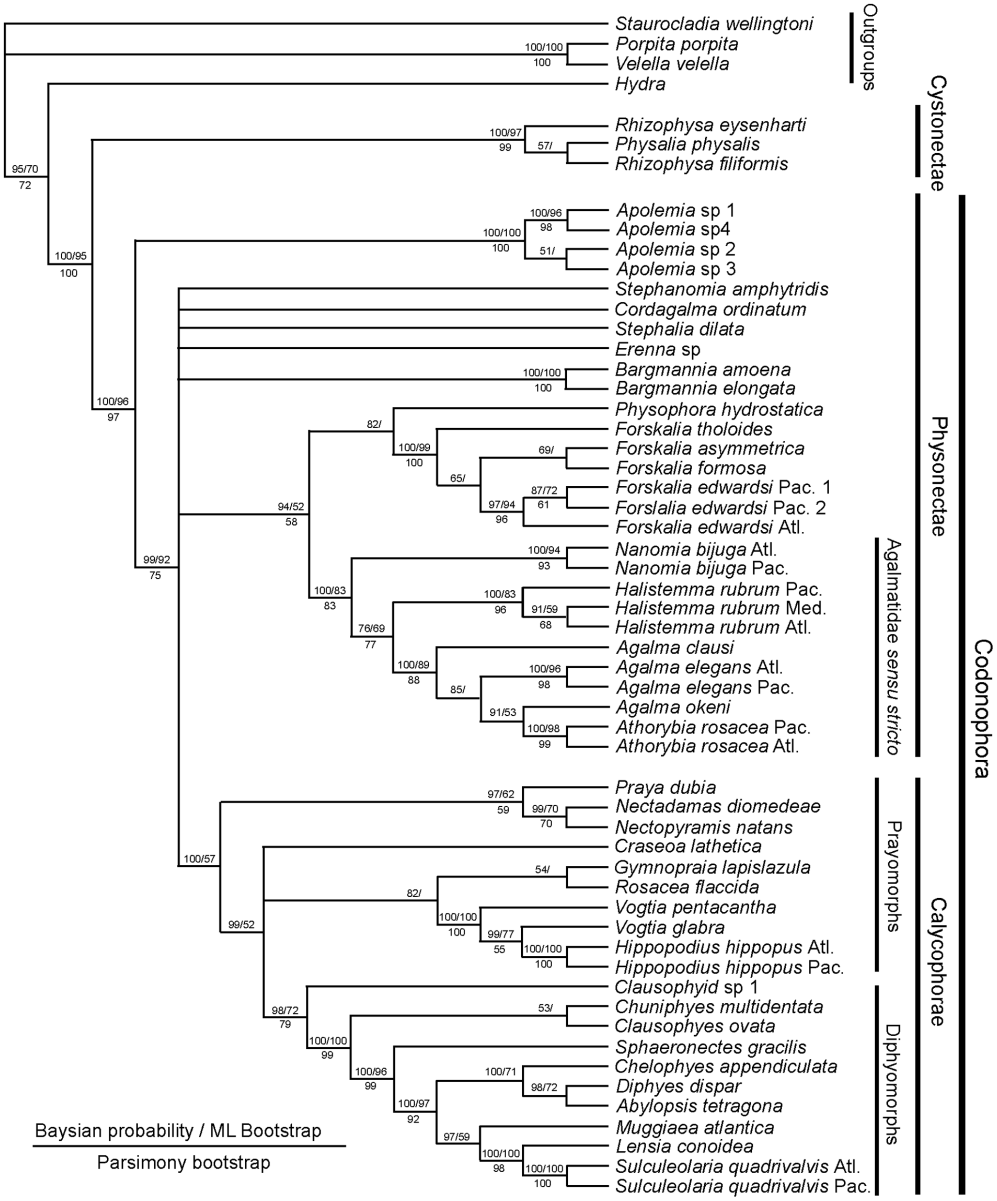
Molecular phylogeny of siphonophores from Dunn et(fig. 6[10]). Consensus tree of all trees for the Bayesian analysis of the combined data set (from an initial 20 million trees). The left score above the branch is the Bayesian posterior probability (%), the right score above the branch is the ML bootstrap support value (%), and the score below the branch is the MP bootstrap support value (%). The bars to the right of the species names indicate clades and grade taxa. Abbreviations: Atl – Atlantic; Med – Mediterranean; Pac – Pacific. For full details of analyses and consensus tree computations refer to Dunn et al. [10].

**Figure 10:**
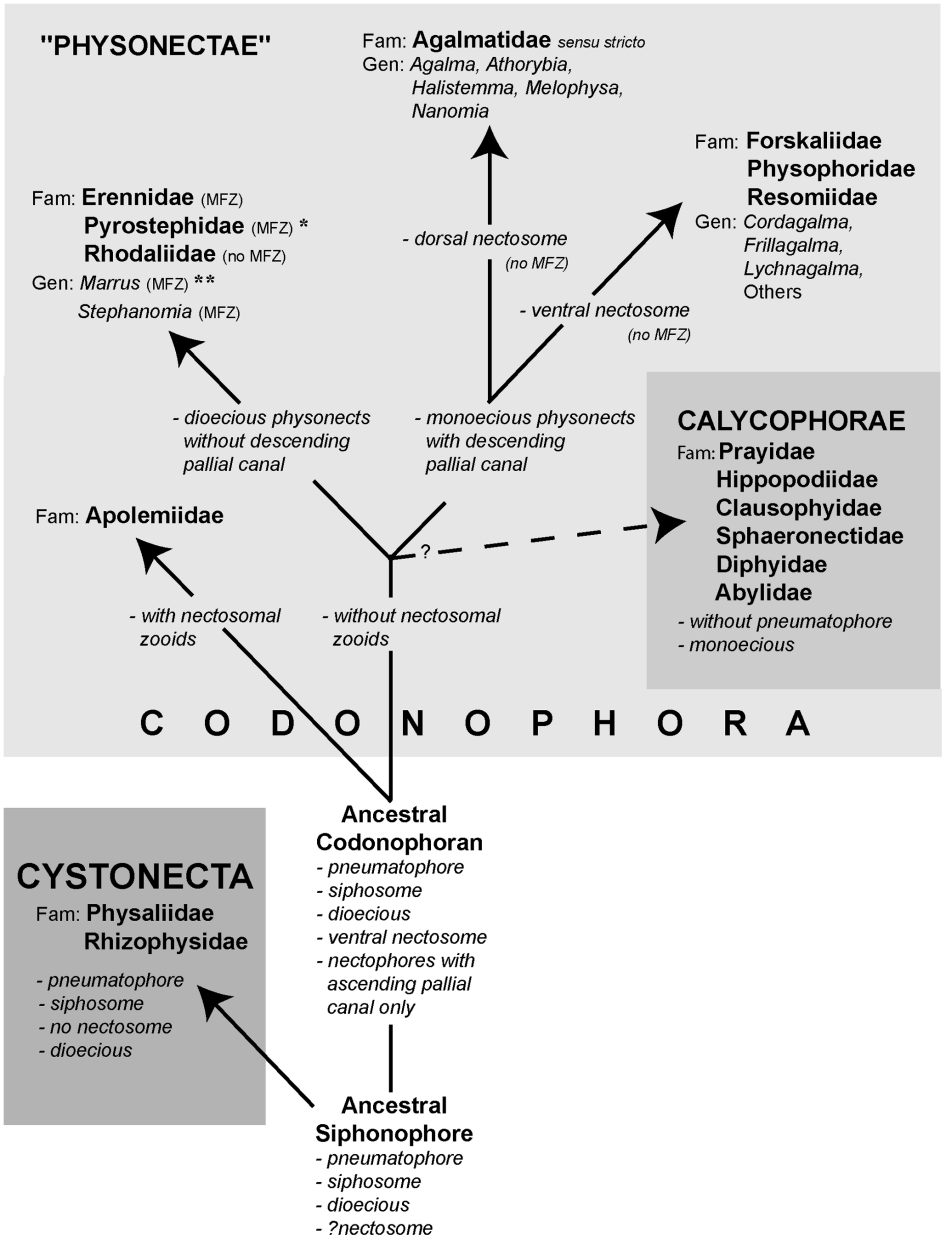
Possible phylogeny of the Siphonophora (derived from [17], fig. 21, and [11]). MFZ – muscle-free zone on nectophore; * - dorsal nectosome; ** - one species monoecious.

The first detailed molecular study of a large range of Siphonophora [10] identified important morphological characters associated with their evolution not previously considered significant; it is reproduced here as Figure 9. A more recent study [79] used the barcoding gene mtCOI to generate a phylogeny for 95 medusozoan species (including 61 siphonophores), though this gene is more appropriate for phylogenetic characters at family level or below. Analysis of a third gene 28S is unresolved for the clade Codonophora [55], and further siphonophore taxa analyses and application of whole genome sequencing to the group are awaited for more clarification of this clade. The study of Dunn et al. [10] led to further changes in physonect systematics by Pugh [17] as discussed below (Figure 10). The old and new phylogenies are compared in Table 3, from 15 families recognized in 1987 and 16 different families and 67 genera recognized today.

**Table 3:** Old and new classification of the Siphonophora.

A. OLD TAXONOMY	B. NEW PHYLOGENY		
High Rank	High Rank	Family & Sub-family	Genera
**Sub-order Cystonectae**			
**Families: Physaliidae, Rhizophysidae**			
	**I - CYSTONECTA**	01. Physaliidae	*Physalia*
		02. Rhizophysidae	*Bathyphysa, Rhizophysa*
	**II - CODONOPHORA**		
**Sub-order Physonectae**	**Physonectae**		
**Families: Apolemiidae, Agalmatidae, Pyrostephidae,Physophoridae, Athorybiidae, Rhodaliidae, Forskaliidae**			
	Dioecious families	03. Apolemiidae	*Apolemia*
		04. Erennidae	*Erenna, Parerenna*
		05. Pyrostephidae	*Bargmannia, Pyrostephos*
		06. Rhodaliidae	*Angelopsis, Aranciala, Dromalia,*
			*Archangelopsis, Steleophysema,*
			*Stephalia, Thermopalia*
		07. Unascribed dioecious genera	*Marrus, Stephanomia*
	Monoecious families	09. Forskaliidae	*Forskalia*
		10. Physophoridae	*Physophora*
		11. Resomiidae	*Resomia*
		08. Agalmatidae *sensu stricto*	*Agalma, Athorybia, Melophysa,*
			*Halistemma, Nanomia*
		12. Unascribed monoecious genera	*Cordagalma, Frillagalma,*
			*Lychnagalma, Rudjakovia*
**Sub-order Calycophorae**			
**Families: Prayidae, Diphyidae, Hippopodiidae, Clausophyidae, Sphaeronectidae, Abylidae**			
	**Calycophorae**		
	Prayomorphs	13. Prayidae	
		S-f Amphyicaryoninae	*Amphicaryon, Maresearsia*
		S-f Prayinae	*Craseoa, Desmophyes, Rosacea,*
			*Gymnopraia, Lilyopsis,*
			*Mistoprayina, Praya, Prayola,*
			*Stephanophyes*
		S-f Nectopyramidinae	*Nectadamas, Nectopyramis*
		14. Hippopodiidae	*Hippopodius, Vogtia*
	Diphyomorphs	15. Clausophyidae	*Chuniphyes, Clausophyes,*
			*Crystallophyes, Kephyes,*
			*Heteropyramis*
		16. Sphaeronectidae	*Sphaeronectes*
		17. Diphyidae	
		S-f Sulculeolariinae	*Sulculeolaria*
		S-f Diphyinae	*Chelophyes, Dimophyes,*
			*Diphyes, Eudoxoides, Lensia,*
			*Muggiaea*
		S-f Giliinae	*Gilia*
		18. Abylidae	
		S-f Abylinae	*Abyla, Ceratocymba*
		S-f Abylopsinae	*Abylopsis, Bassia, Enneagonum*

The consensus tree from the molecular study of Dunn et al. (see fig. 6 [10]) is based on data from two genes: the nuclear gene 18S and mitochondrial gene 16S, and is figured here as Figure 9. It concludes that cystonects are sister to all other siphonophores, with the remainder ranked together in a new clade Codonophora, meaning ‘bell bearers’. Within the Codonophora clade, the traditional grouping ‘Physonectae’ are paraphyletic, with the ‘physonect’ family Apolemiidae sister to all other taxa. Clades for the physonect families Forskaliidae and Agalmatidae *sensu stricto* are well supported, although resolution for taxa representing rhodaliids, erennids, pyrostephids and physophorids is poor. The traditional group Calycophorae are nested within the non-apolemiid Codonophora and form a monophyletic clade. Within the Calycophorae, prayomorphs are paraphyletic, based on taxa and genes sampled in 2005. Hippopodiid prayomorphs form a distinct clade, and diphyomorphs, together with *Sphaeronectes* (ignoring one undescribed clausophyid species) form another distinct clade. Intraspecific variation is also demonstrated in multiple individuals of *Hippopodius hippopus* and *Sulculeolaria quadrivalvis* collected in the Atlantic and Pacific Oceans. The final important finding of Dunn et al. [10] places abylids within a clade containing the five diphyids tested. Five cryptic species pairs are also identified amongst the Atlantic and Pacific ‘physonects’ analysed (Figure 9).

The new phylogeny shows that character evolution within the Siphonophora is related to reproductive state (figs. 7–8 [10]). Separately sexed individuals are dioecious, whereas monoecious siphonophores bear differentially maturing male and female gonophores on the same individual. Zooid types scored by Dunn et al. [10] include nectosomal nectophores, siphosomal bracts, gastrozooids and palpons, as well as the number of types of each zooid present in each taxon. They found that cystonects, apolemiids, pyrostephids, erennids and rhodaliids, are all dioecious, and, surprisingly, all lack a descending ‘pallial canal’ (‘descending surface diverticulum’ of Mapstone [6]) on the proximal surface of the nectophore. In contrast, all remaining codonophorans are monoecious, and in taxa tested from the families Agalmatidae *sensu stricto*, Forskaliidae and Physophoridae (except *Athorybia rosacea* which lacks nectophores) this condition is coincident with the presence of a descending ‘pallial canal’ on the proximal surface of the nectophore.

Nectosomal nectophores are an apomorphy of the Codonophora and may have been derived from retained reproductive medusae [10]. Their presence together with the presence or absence of a descending pallial canal, suggests these two characters might have pleiotropic links [10]. Many of one type of nectophore (homomorphic) were found in all the ‘physonects’ tested except *Athorybia rosacea*, which lacks nectophores. Amongst the Calycophorae, nectophores are reduced to two of one type in most prayomorphs tested, except for the two nectopyramidines which had only one of one type, and hippopodiids which, as Dunn et al. [10] conclude, have developed several nectophores of one type from an ancestor which probably had only two of one type (see their fig. 8a). Most diphyomorph calycophorans, in contrast, have two nectophores of two types (an anterior and a posterior: heteromorphic), with one nectophore lost in *Muggiaea atlantica*, and only a single larval nectophore retained in *Sphaeronectes gracilis* (fig. 8a [10]).

Palpons are another character found in almost all ‘physonects’, but absent from all the calycophorans tested by Dunn et al. (fig. 8b [10]). Parsimony indicates that palpons were probably present in the ancestral siphonophore and have been lost one or two times, while bracts appeared first in the Codonophora, and might have developed into two or more types several times and at several different specific locations during siphonophore evolution [10]. Bracts, however, which are also characteristic of the Codonophora, are all of one type in apolemiids and also in all calycophorans which possess them, as well as in some Agalmatidae *sensu stricto* (*Agalma* and *Athorybia*). In erennids and other Agalmatidae *sensu stricto* (*Nanomia* and *Halistemm*a species) two types of bracts develop, and four types are found in *Forskalia* species (see fig. 8b in [10]). Thus, as Dunn et al. [10] conclude, there has been both gain and loss of zooids during siphonophore evolution.

The importance of these characters in shaping siphonophore evolution is reflected in the higher rankings given in Table 3B. A new hypothesis for character evolution given by Pugh [17], which is shown here in Figure 10, proposes a dioecious ancestral siphonophore with pneumatophore and siphosome, but maybe not a nectosome. Such an ancestor may have given rise to two clades: the dioecious cystonects with a pneumatophore and siphosome but no nectosome, and a dioecious ancestral codonophoran with a pneumatophore, nectosome and siphosome. Nectophores of the latter have only an ascending ‘pallial canal’ on their proximal surfaces. The first nectosome to evolve is thought to have had all nectophores attached on the same side of the stem as the siphosomal zooids, which is, by convention, the ventral surface of the stem (p. 931 [10]). A similar condition is found in most families and genera of physonects today.

Apolemiids are also dioecious, with nectosomal palpons between the nectophores [11], [80], and were the first group to split from the other Codonophora, with both lineages evolving simultaneously and independently thereafter. The ancestral sister group to the Apolemiidae could have been another clade that lacked nectosomal palpons (Figure 10) and from which, perhaps later in time, a monoecious ancestor emerged. Dioecy could have persisted in a group of physonects which lacked a descending ‘pallial canal’ on the proximal surface of their nectophores, including three extant families and two unascribed dioecious genera (see Table 3B). In one of these families, the Pyrostephidae, a twist may have occurred at the junction between the nectosome and siphosome resulting in nectophores arising on the dorsal surface (‘dorsal nectosome’) and siphosomal cormidia on the ventral surface. The first monoecious siphonophores could have been physonects with a descending ‘pallial canal’ on the proximal surface of their nectophores, a new diagnostic character. From this clade Pugh [17] proposes a split into the Family Agalmatidae *sensu stricto* with a dorsal nectosome, and a non-agalmatid clade including the families Forskaliidae, Physophoridae and Resomiidae together with the unascribed monoecious genera *Cordagalma*, *Frillagalma* and *Lychnagalma* (Table 3B) which all exhibit a ventral nectosome (Figure 10). Pugh [17] also suggests that a further monoecious group of siphonophores, the Calycophorae, appeared at some point during the evolution of these various physonect families, (Figure 10). In calycophorans the pneumatophore is lost and the nectosome typically reduced to just two nectophores.

### Systematics 1987 to Present

This section summarizes the changes in siphonophore systematics since the last review in 1987 and is based on the new phylogenies as outlined above [10], [17], together with details of families that have been revised or newly introduced, and new genera and species added, moved or now considered invalid. Most of this information for cystonects and physonects is given in Table 4, and for calycophorans in Table 5. Ongoing debates about the validity of certain species, and other systematic information too extensive for inclusion in the tables, is briefly summarized below.

**Table 4:** New systematics for cystonect and physonect siphonophore families.

	Family	Comments
01.	Physaliidae	Monotypic for *Physalia physalis* (*P. utriculus* considered a junior synonym [81]
02.	Rhizophysidae	Long-stemmed; *Bathyphysa japonica* a junior synonym of *B. conifera;* SEM studies of budding sequences described for *B. sibogae, Rhizophysa filiformis* and *R. eysenhardti* [8]
03.	Apolemiidae	Long stemmed; monophyletic and sister to all other Codonophora, with unique nectophoral palpons on the nectosome. Nectophores distinctive and ridge-less, cormidia dispersed or discrete; gastrozooids with simple tentacles (no tentilla) resembling palpacles of palpons. Monogeneric for *Apolemia*. Two new species include *A. lanosa* and *A. rubriversa* [11] and three older species *A. contorta, A. uvaria* and *A. vitiazi* (*Tottonia contorta* sensu Mapstone 2003 now referable to *A. lanosa*). A number of other species are known to exist [2], [10], [11], [52], [82], [83], [84], [85], and await full description.
04.	Erennidae	Long-stemmed family erected for 4 species with large prominent straight tentilla, no involucrum and a rigid terminal process lacking nematocysts [15]. Two genera: *Erenna* (3 species) and *Parerenna* (1 species). *E. richardi* Bedot, 1904, and a new species *E. laciniata* have large flattened nectophores and large tentilla held close to body and vibrate to attract prey; two further new species *E. cornuta* and *Parerenna emilyae* have different and also unique tentilla and gastrozooids [15].
05.	Pyrostephidae	Long-stemmed family reviewed and revised [14], with 3 new species of *Bargmannia*: *B. amoena, B. gigas, B. lata* [14], [86]; also *Mica micula*, the putative post-larva of a pyrostephid [87], [88]. Nectophores with unique lower-lateral wings and much enlarged triangular thrust block; in *B. elongata* two growth zones on stem and composition of the cormidia studied using SEM [80]; pyrostephid cormidia either have oleocysts (modified tentacle-less palpons) (in *Pyrostephos*) or none (in *Bargmannia*) [14].
06.	Rhodaliidae	Short-stemmed family of 8 genera, with 4 new species including *Archangelopsis jagoa*, *Arancialia captonia* [45], [89], and two others herein referred to *Steleophysema* Moser, 1924, including *S. sulawensis* and *S. rotunda*. *Sagamalia hinomaru* reduced to a junior synonym of *Steleophysema aurophora* [1], [89]. First *in situ* feeding observations on four species [89]. *Dromalia alexandri* re-described [4].
07.	Unascribed dioecious genera	Long-stemmed genera *Marrus* Totton, 1954 [90] and *Stephanomia* Lesueur & Petit, 1807 [10] both with muscle-free zones on nectosac and other characters (Fig. 10). A new species *M. claudanielis* described [90] and new specimens of an old species *S. amphytridis* [10] await re-description.
08.	Forskaliidae	Long stemmed and delicate monotypic family, probably sister to the Physophoridae [10]. Recently revised [16] with two new species added (*Forskalia asymmetrica, F. saccula*) and one reduced to a Species Inquirenda [1].
09.	Physophoridae	Family with long nectosome but short corm-like siphosome; previously monotypic for *Physophora hydrostatica* bract present only in larva; now a new smaller and less colourful second species *P. gilmeri*, is added, with bracts retained on adult colony [77]; unique tentilla in both species.
10.	Resomiidae	Long-stemmed family newly introduced for two species previously referred to the Agalmatidae (*Moseria convoluta*, *M. similis*) and now transferred to a new monotypic genus *Resomia* [17]; two tentilla types uniquely present on each tentacle. Three new species *R. dunni*, *R. ornicephala*, *R. persica* described in 2010 [91].
11.	Agalmatidae *sensu stricto*	Mostly long-stemmed and recently restricted to genera with dorsal nectosome (see above) and involucrate tricornuate tentilla with tightly coiled cnidoband (see below). Now includes two short-stemmed genera (*Athorybia*, *Melophysa*) [17]. New species added (*Halistemma transliratum*) [92] and another re-described (*H. foliacea*, as *H. amphytridis*) [17], [93].
12.	Unascribed monoecious genera	Long-stemmed monotypic genera *Cordagalma, Frillagalma* and *Lychnagalma* with ventral nectosomes have been removed from the Agalmatidae [17] and a new species *C. tottoni* described [94]. *Rudjakovia plicata* considered a valid species [1] and may be transferred to Agalmatidae when more characters are elucidated [17].

For fundamental characters of the physonect families listed above (sex, proximal surface canals etc), see Figure 10.

**Table 5:** New systematics for calycophoran siphonophore families.

Family	Comments
13. Prayidae	Probably paraphyletic, and includes nested family Hippopodiidae [10] (see below); *Praya dubia* (Subfamily Prayinae) and sub-family Nectopyramidinae maybe one lineage, with prayines *Craseoa*, *Gymnopraia* and *Rosacea* another [10], but broader taxa sampling is needed [6]. Prayine name *Lilyopsis medusa* has precedence over *Lilyopsis rosea* [1]; new prayine species *Desmophyes haematogaster*, *Gymnopraia lapislazula*, *Lilyopsis fluoracantha, Rosacea repanda*, *R. limbata*, *R. arabiana* introduced (see [1]); subfamily Nectopyramidinae revised [13] with *Nectopyramis thetis* and *N. natans* re-described and new genus *Nectadamas* introduced (for *N. diomedeae* and a new species *N. richardi* [13]). Prayine species *R. cymbiformis* also re-described [99] and nomenclature problems concerning *R. plicata* sensu Bigelow and *Desmophyes annectens* resolved [100], [101]. Eudoxids are released in amphicaryonines and nectopyramidines, but not in prayines [6]. *Rosacea villafrancae* transferred to genus *Desmophyes* [102], and *Prayoides intermedia* found to be a junior synonym of *Praya* species [1], [103]. Unique bio-optical properties identified in *G. lapislazula* and *L. fluoracantha*, though their function is still unknown [7].
14. Hippopodiidae	Found nested within prayines in first siphonophore phylogeny, and *Hippopodius* nested within *Vogtia* [10]; hippopodiid distribution correlated with feeding on various species of ostracods, unlike other calycophorans [104]. Family characters recently summarized and the new axes applied, together with re-descriptions given and synonomies listed for *V. serrata*, *V. spinosa* and *V. pentacantha* [6]; *V. microsticella* considered a junior synonym of *V. glabra*, and *V. kuruae* a junior synonym of *V. serrata* [1], [6].
15. Clausophyidae	The 3 diphyomorph families below may have arisen from the Clausophyidae [10]. New species include *Clausophyes laetmata* [42] and *Cl. tropica* [105] and 2 others re-described include *Cl. galeata* and *Cl. moserae* [105]; a unique fuseudoxid life stage found in *Crystallophyes amygdalina* [47] and a new genus *Kephyes* introduced for Moser’s *Cl. ovata*, which, unlike *Clausophyes* species, has bracts with a pair of hydroecial canals [106]. 4 clausophyids re-described from NE Pacific and the new axes applied [6].
16. Sphaeronectidae	Ten species now considered valid in this family with single retained larval nectophore. Family reviewed and history summarized [18]; 5 new species introduced: *Sphaeronectes christiansonae*, *S. haddocki*, *S. tiburonae* [18], *S. pagesi* [107] and *S. pughi* [108]. An old species *S. brevitruncata* reinstated [18] and *S. bougisi* concluded to likely be a calyconula of *Lilyopsis medusa* [1]. *S. gracilis* relegated to a junior synonym of *S. koellikeri* and probably restricted to the tropics [1], [18]; specimens reported from Jervis Inlet, British Columbia [6] probably another species.
17. Diphyidae	Probably paraphyletic [10], vindicating earlier conclusions [9], but based on only 5 of 43 likely valid species [1]. Two main clades identified in the molecular study of Dunn et al. [10], within one of which is nested the Family Abylidae. New axes applied to all life stages of diphyids, muscular lamellae, median gastrovascular canals and pedicular canal arrangements also schematically shown for two basic types of diphyids [6]. A new small species added to genus *Lensia* (*L. quadriculata* [109]), another re-described in detail (*L. asymmetrica* [110]) and a third (*L. reticulata*) transferred to a new genus *Gilia* within a new subfamily Giliinae, for the two clausophyid-like canals in the bract (*G. reticulata* [111]). An enigmatic species *Eudoxia macra* shown, using the mitochondrial 16S gene, to be sexual stage of a larger species *L. cossack* [112]. A number of previously described *Lensia* species, several *Sulculeolaria* species and one *Muggiaea* species all reduced to junior synonyms of various better known species [1].
18. Abylidae	Family nested with *Diphyes dispar* in one of two Diphyidae clades, based on 16S and 18S [10], but only *Abylopsis tetragona* tested and more taxa sampling needed. 10 valid species [1], all present in the S Atlantic and summarized in a recent report [113]; several species also re-described from around South Africa [82], [114]. Junior synonyms (including those in a confusing abylid review by Sears [115]) given in the Worms World List [1].

#### Apolemiidae

Unique nectosomal palpons (previously nectosomal tentacles or polyps) are probably a synapomorphy of the Codonophora, being greatly reduced or absent in other codonophorans [10]. These zooids arise on the nectosome from the posterior ends of the nectophoral muscular lamellae, either singly or in bunches [85], and are identified as small buds on the nectosome of some other long-stemmed physonects [8]. Other important specific characters include the presence or absence of diverticula penetrating into the mesogloea from the lateral radial canals of the nectophores, the relative size of the siphosomal horn, the type of siphosomal cormidia present (pedunculate or dispersed), and the number of palpon types on the siphosome (one or two) [11]. In older cormidia, secondary gastrozooids may form independent of the growth zone, directly on the siphosome [11], as also shown in the agalmatid *Nanomia bijuga* [8] (see above). Pedunculate cormidia may be either ancestral or derived for the Codonophora [11], but further work and denser sampling of siphonophore phylogeny is needed to resolve this question [11].

Currently, the family is monotypic for *Apolemia,* and includes *A. uvaria* (Lesueur, 1815), *A. vitiazi* (Stepanjants, 1967) and *A. contorta* (Margulis, 1976) [1], together with two newly described species *A. lanosa* and *A. rubriversa* [11] and a third species not yet described (*A. trinegra* [84]). Two types of siphosomal palpons are exhibited by *A. uvaria* (shorter red/brown type and longer opaque type [85], [95]), but may also be characteristic of other species, together with pigment distribution in the siphosomal palpons [84]. Apolemiids can reach more than 30 m in length, and the recent paper by Siebert et al. forms the foundation for descriptions of up to 15 further new species [11]. Apolemiids frequently undergo autotomy [6], [95], releasing many lengths of siphosome which float freely in the water without nectophores, while the latter swim off or drifted away in a different direction.

#### Erennidae

Collection of several excellent quality specimens by submersible from the Dry Tortugas and Bahamas in the tropical Atlantic has enabled introduction of this new family, with three new species and an older re-described species (Table 4 and [15]). These deep-sea physonects have much enlarged tentilla that are held close to the body and in most species vibrate to attract prey (deep-sea fish); these lures are described in a later section.

#### Pyrostephidae

This family has been properly diagnosed for the first time and three new species introduced [14]. A likely pyrostephid post-larva has also been described (Table 4), and a comprehensive study of the organisation of siphosomal zooids in *Bargmannia elongata* shows that new cormidia are formed on a protrusion from the stem termed the “horn” [80]. Here a series of “probuds” form, which each subdivides a number of times to form eight zooids and together these form a single cormidium (see above).

#### Rhodaliidae

Four new species have been added to this epibenthic family in recent years (Table 4 and [45], [89]). *Dromalia alexandri* has been re-described including the first figures of a rhodaliid siphosomal horn, mature cormidial units and a mature bract, together with a more comprehensive distribution map including both range and density [4]. Herein the doubtful species *Steleophysema aurophora* Moser, 1924 [27], is re-validated from observations made by Dhugal Lindsay (pers. comm.) of new specimens collected off Japan, and as a result *Sagamalia hinomaru* is reduced to a junior synonym [1]. The genus *Tridensa* Hissmann, 2005, is also reduced to a junior synonym of *Steleophysema*, based on the shape and attachment point of its bracts (at base of each cormidial unit), attachment of the gonophores (with egg pouch) directly to the thin polygastric cormidia just distal of each cormidial gastrozooid, and attachment of the gonopalpons just distal of the gonophores. The two species *T. sulawensis* and *T. rotunda* become junior synonyms of *S. sulawensis* and *S. rotunda* [1]. A full re-description of *S. aurophora* is underway (D. Lindsay, pers. comm.).

#### Unascribed dioecious physonects

The genera *Marrus* and *Stephanomia* perhaps diverged early from other codonophorans (Figure 10). *Marrus orthocannoides* may not belong to the genus *Marrus*, because it has a fully muscular nectosac, whereas those of other *Marrus* species have a proximal muscle-free zone [90]. The genus name *Stephanomia* has been applied to many species in the past (p. 102 [6]), but is herein restricted to the large species *Stephanomia amphytridis* of Lesueur and Petit, 1807 [1] as applied by Dunn et al. [10] and mentioned on p. 103 of Mapstone [6]. This species has been collected recently in both the Atlantic and Pacific [17], sequenced for 16S and 18S genes [10] and a morphological description is underway.

#### Forskaliidae

The fragile and often snake-like colonies of this monoecious family have a spiral stem with diffusely attached zooids. New material, much obtained by SCUBA diving, has allowed a reassessment [16] that retains four older species (*F. edwardsi*, *F. contorta*, *F. formosa* and *F. tholoides*), adds two new species (Table 4) and reduces *F. leuckarti* to a junior synonym of *F. contorta* [1]. The recent molecular analysis supports monophyly of this family (Figure 9), which uniquely possesses (for most species) four bract types [10], with one type on the stem (stem bracts) and three types on the elongate pedicels of the gastrozooids (bolster and two types of knee-shaped bracts) [16]. A single gonodendron also occurs on the stem, between two gastrozooids, and carries bunches of both male and female gonophores which can be attached in a species-specific pattern [16].

#### Resomiidae

Live colonies of this new monoecious family are mostly transparent with a short rigid siphosome that never relaxes, as also in species of *Erenna* and the agalmatid *Agalma okeni*. Three of the five species referred to the family are new (Table 4), and all are characterized by tentilla on the same tentacle which undergo transformation from a spirally coiled cnidoband to a zig-zagged cnidoband [17], a process superbly illustrated in colour for the three new species by Pugh and Haddock [91]. In the new species *Resomia ornicephala*, the involucrum floats above the cnidoband and fluoresces under incident blue light, attracting krill prey, as described further below.

#### Agalmatidae

A *sensu stricto* clade of this family has been identified from the molecular phylogeny of Dunn et al. [10] and includes three long-stemmed and two short-stemmed genera (Figures 9, 10, Table 4); all have tricornuate tentilla and tightly coiled cnidobands. A new species *Halistemma transliratum* from the Bahamas has nectophores with a single vertical-lateral ridge and three types of bract [92], whilst nectophores from another giant *Halistemma* species (*H. foliacea*) have been described for the first time [93] from Indonesian waters (Table 4); the latter species has nectophores with two vertical-lateral ridges and three types of thick foliaceous bracts. Both species have unicornuate tentilla with a vestigial involucrum and a long terminal filament terminating in a small cupulate process or sinker (see below). Cormidial development has been elucidated for *Agalma elegans* and *Nanomia bijuga*, using a SEM, and zooids found to develop differently from pro-buds in each species [8]. Tissue samples from very young nectophores and gastrozooids of *N. bijuga* have also been analysed (using next generation sequencing [96]) for gene expression in wild specimens, and a gene expressed only in the basigaster of the gastrozooid that encodes for a protein used in the formation of the nematocyst wall further characterized [96].

#### Unascribed monoecious physonects

The three monoecious genera with a ventral nectosome noted in Figure 10 and Table 4 have unique tentilla, and two of them (*Frillagalma* and *Lychnagalma*) are monotypic [1]. Two new species await description in the genus *Cordagalma* [17], and a re-description of *F. vityazi* from new submersible material shows that frilling of the ridges in the nectophores and bracts of the original net-caught specimens is a preservation artefact [97]. Sequencing of the 16S gene of *L. utricularia* shows its closest relations to be members of the family Physophoridae [17]; *L. utricularia* was also found to be the only non-bioluminescent physonect in the Alboran Sea [98]. Fresh specimens of a fourth unassigned monoecious physonect, *Rudjakovia plicata*, taken recently off California indicate that their much pleated nectosacs are also preservation artefacts. The nectophores of this species attach to the dorsal side of the nectosome, indicating that it may be referable to the Agalmatidae *sensu stricto*, but further material is needed to confirm this hypothesis.

#### Prayidae

Absolute axes applied to the colony, stem and zooids of two new prayine species in this family facilitate consistent future species descriptions [7], and are extrapolated to a further nine prayid species in another publication [6]. The prayid somatocyst is also redefined [6] to bring the terms applied to prayid proximal nectophore canals into line with those used for the homologous canals and diverticula in both physonects (which lack a somatocyst) and diphyomorph calycophorans (which have a somatocyst that penetrates into the mesogloea and develops from only one diverticulum of the pedicular canal). Bracts, larval nectophores and young definitive nectophores of *Praya dubia* and *P. reticulata* have been reliably distinguished for the first time since 1987 [103] and their mature nectophores also fully described from new specimens collected in the NE Pacific [6]. The recent siphonophore molecular phylogeny of Dunn et al. [10] suggests that Prayidae are paraphyletic, with *Praya dubia* and two nectopyramidines forming one clade and three other prayines forming a second (Figure 9).

#### Clausophyidae

New information on this diphyomorph family is given in Table 5, and its position intermediate between the Prayidae and Diphyidae is well shown in a figure by Mapstone (fig. 4 [6]). A useful time line is also given by Pugh [106] for descriptions of three widespread clausophyid species (*Clausophyes galeata, C. moserae, Kephyes ovata*). New deep-water records from various locations worldwide contribute further to our understanding of the ecology of this deep-water family [6], [42], [47], [87], [105], [106], [109], [116], [117], [118], [119], [120], and two further new clausophyid species await description [10], [106].

#### Sphaeronectidae

A recent and thorough review of this diphyomorph family is given by Pugh [18], together with an updated systematic treatment of all valid species [1]. Beautiful images are available for six of the ten small species now comprising this family [18], [107], and new siphonophore axes are extrapolated for sphaeronectids by Mapstone [6]. These axes are incorporated into descriptions of the two most recently introduced species [107], [108]. For a useful schematic summary of the sphaeronectid life cycle see fig. 15 in [18].

#### Diphyidae

The first new *Lensia* species introduced for 36 years is *L. quadriculata* (Table 5 and [109]), and another, *L. asymmetrica*, is re-described with its posterior nectophore, bract and gonophore identified for the first time [110]. New bracts of a third small species *L. reticulata* indicate a close affinity to the family Clausophyidae for which it is transferred to a new subfamily (Table 5 and [111]), and a previously unassigned eudoxid referred to the large diphyid *Lensia cossack* (Table 5 and [112]). Seven diphyid species are recorded for the first time in Japanese waters [116].

### Nematocysts and Lures

Nematocysts and tentilla were only briefly covered in the 1987 review of siphonophore biology [2], and are therefore described here in more detail.

#### Nematocysts

**Figure 11:**
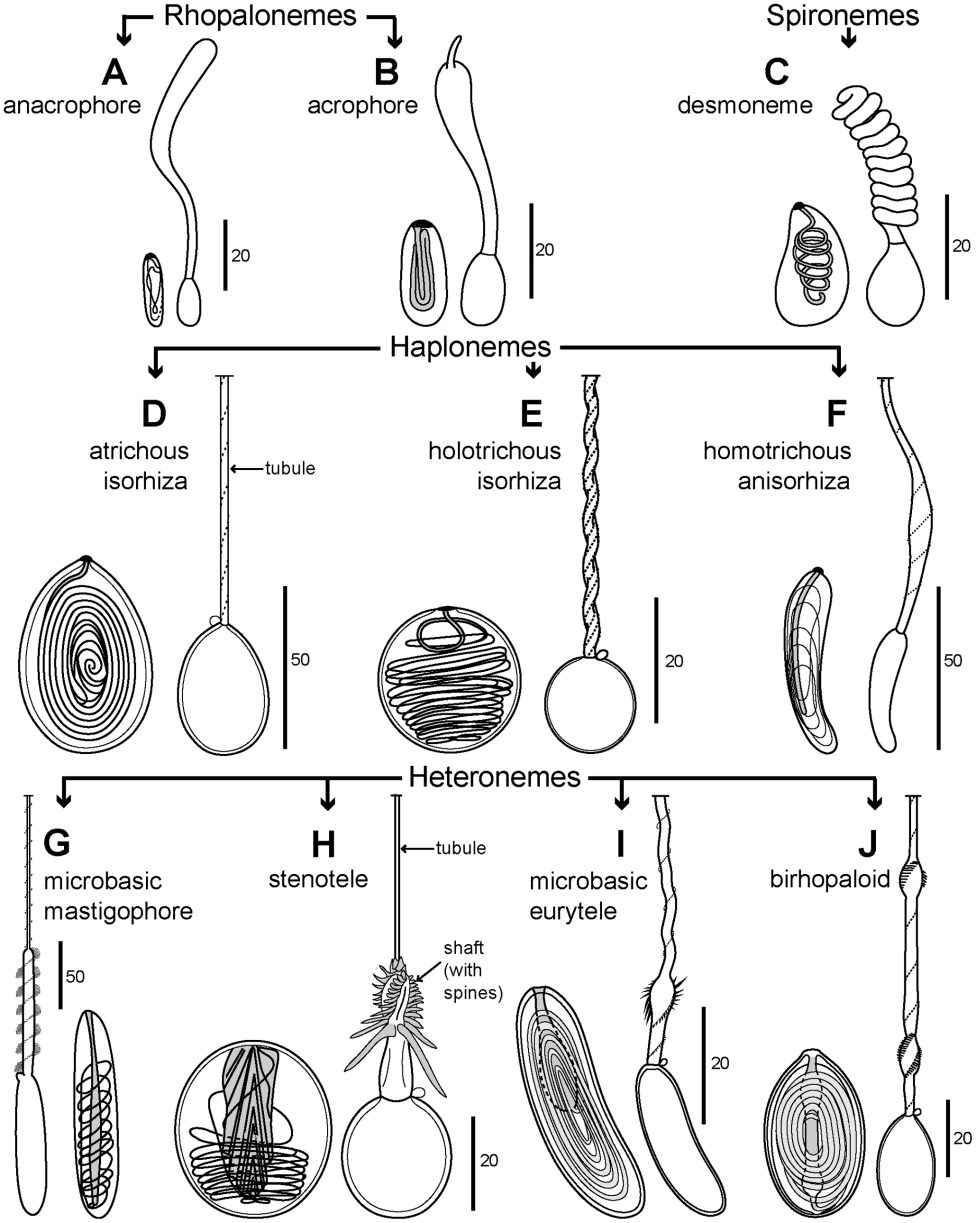
Schematic representation of ten nematocyst types found in Siphonophora. Undischarged and discharged nematocysts included. A: anacrophore rhopaloneme (after fig. 22a–b [122]); B: acrophore rhopaloneme (after fig. 23a–b [122]); C: desmoneme spironeme (after fig. 26a–b [122]); D: atrichous isorhiza haploneme (after fig. 4a–b [123]); E: holotrichous isorhiza haploneme (after figs. 1a, 1b [124] and fig. 7b [123]); F: homotrichous anisorhiza haploneme (after fig. 41a–b [122]); G: microbasic mastigophore heteroneme (derived from fig. 29 [127] and fig. 2a [124]); H: stenotele heteroneme (derived from fig. 17 [127] and fig. 1d [124]); I: microbasic eurytele heteroneme (after pl. 1, figs. 6–7 [132]); J: birhopaloid heteroneme (after fig. 83 [122] and fig. 3d [124]).

Nematocysts are an apomorphy of the Cnidaria, and one of three types of cnidae which characterize the phylum; the others are ptychocysts and spirocysts (absent from Hydrozoa). More than 30 types of nematocyst are recognized and their classification is typically based on characters of the tubule (open or closed tip, diameter, presence or absence of a swollen shaft at the proximal end, pattern, distribution and size of spines on the tubule). Diversity among nematocysts, different methods of classifying them and the possible importance of cnidae in cnidarian evolution are reviewed by Fautin [121]. The total complement of cnidae in a species is termed the cnidome (p. 68 [6]). A summary of nematocyst characteristics of most siphonophore families and some genera and species is given in Figure 11 and Table 6. Five types are autapomorphic (exclusive) to Siphonophora, including two categories of rhopalonemes (acrophores and anacrophores), haploneme homotrichous anisorhizas and two categories of heteroneme rhopaloids (shaft of unequal diameter with either two swellings along its length (birhopaloids) or one (rhopaloids)) (Figure 11).

**Table 6:** Nematocysts of siphonophores.

Family	Des	Acro	Anacro	Atrich Iso	Holotrich Iso	Homo Aniso	Steno	Micro Mastig	Micro Eury	Birhop	References
Physaliidae	–	–	–	isorhizas		–	yes	–	–	–	[81], [125]
Rhizophysidae	–	–	–	isorhizas		–	–	–	–	–	[67], [126]
Apolemiidae	–	–	–	isorhizas (2 sizes)		–	yes(2 sizes)	yes (or unknown type)	one sp. probably	in one+sp.(2 sizes)	[11], [124], [127]
Pyrostephidae	yes	rhopalonemes		–	–	–	yes	–	–	–	[14]
Erennidae	–	–	–	isorhizas		yes	–	–	–	–	[15]
Rhodaliidae	yes	rhopalonemes		–	–	yes	–	yes	yes	–	[27], [45], [89]
*Marrus* spp.	yes	yes	–	–	–	yes	–	yes?	yes	–	[90]
Forskaliidae	yes	yes	–	?	–	yes	yes	–	–	–	[122]
Physophoridae	–	–	–	–	–	yes	yes	yes	–	–	[77], [128], [129]
Resomiidae	yes	yes	–	–	–	yes	–	yes	–	–	[17], [91]
*Agalma* spp.	yes	yes	–	–	–	yes	–	yes	yes	–	[67], [73]
*Athorybia rosacea*	yes	rhopalonemes		–	–	yes	yes	–	–	–	[67]
*Halistemma* spp.	yes	yes	–	–	–	yes	yes	–	yes	–	[92], [73]
*Nanomia* spp.	yes	rhopalonemes		–	–	yes	yes	–	–	–	[67]
*Cordagalma*	–	–	–	yes	–	yes	yes	–	–	–	[130]
*Frillagalma vityazi*	–	–	–	–	–	yes	yes	–	–	–	[97]
*Lychnagalma utricularia*	?	rhopalonemes		–	–	yes	yes	–	–	–	[131]
Calycophorae											
*Rosacea* spp.	yes	–	yes	–	yes	yes	–	–	yes	–	[46], [67]
*Desmophyes villafrancae*	yes	–	yes	–	–	yes	–	–	yes	–	[46], [132]
*Prayola tottoni*	yes	–	yes	–	–	–	yes	–	–	–	[133]
*Lilyopsis medusa*	yes	–	yes	–	–	yes	–	yes	–	–	[134]
*Nectadamas diomedeae*	yes	–	–	–	–	probably	probably	?	–	–	[13], [38]
Hippopodiidae	yes	rhopalonemes		–	–	yes	–	yes	–	–	[67]
Sphaeronectidae	yes	–	yes	–	–	yes	–	yes	–	–	[18]
Diphyidae	yes	–	yes	–	yes	yes	–	yes	–	–	[67]
Abylidae	yes	–	yes	–	yes*	yes*	–	yes	–	–	[67]*except *E.hyalinum*

**Key:** Des - desmoneme; Acro - acrophore; Anacro - anacrophore; Atrich Iso - Atrichous Isorhiza; Holotrich Iso – holotrichous isorhiza; Homo Aniso - homotrichous anisorhiza; Steno – stenotele; Micro Mastig – microbasic mastigophore; Micro Eury – microbasic eurytele; Birhop – birhopaloides (two swellings on tubule).

Identification of nematocysts can be difficult, requiring examination of discharged tubules, although some larger types can be recognized *in situ* undischarged [124]. Successful discharge is best achieved with live material, though the procedure requires practice; discharging nematocysts from preserved specimens is not usually possible [121]. Smaller nematocysts are also more difficult to identify than larger examples, with the result that it has not been possible to identify specific rhopalonemes and smaller isorhizas in some siphonophore species (Table 6).

Although homotrichous anisorhizas occur in most siphonophore groups, they and the other three types of autapomorphic nematocysts are absent from cystonects (Physaliidae and Rhizophysidae in Table 6), which have simple tentacles. Cystonect cnidomes are composed almost exclusively of isorhizas, the most primitive type of cnidarian nematocyst [135]; these nematocysts can be present in enormous quantities, particularly in the tentacles of the Portuguese Man O’War *Physalia physalis*. Ultrastructure of the smaller isorhiza of *Physalia* was studied for the first time by Hessinger & Ford [136], enlarging upon an earlier light microscope study by Will [137]. The nematocyst capsule is held in position by a complex fibrillar basket anchored to the underlying mesogloea with hemidesmosomes and apically by enveloping processes from neighbouring epithelial cells [136]. Such basal anchoring fibrils, often termed a cnidopod (p. 114 [122]), also occur in the nematocysts of other Hydrozoa (p. 29 [138]). In *Physalia*, nematocysts are formed in basigasters (ampullae of Totton [39]) separated from their gastrozooids during development; each nematocyst migrates down either a tentacle to the nematocyst battery region (isorhizas) for prey capture or to gonopalpons in a cormidium (stenoteles), probably for defence of the spherical gonodendron after release from the colony (Table 6 and [81]). Rhizophysid tentacles have side branches with either a strip of isorhizas along one side (e.g. *R. eysenhardti*) or isorhizas in pads on swellings at the distal ends of the branches (e.g. *R. filiformis* fig. 5F [67]). Cystonects consume only soft-bodied prey, mainly fish and fish larvae, and when present in large numbers can deplete fish stocks [67], [139], [140].

The cnidome of apolemiids also reflects a diet of soft-bodied prey [67] and was studied in detail in *Apolemia uvaria* from the Mediterranean [127], in another apolemiid from off California [124] and recently in *A. lanosa* and *A. rubriversa* from Monterey Bay [11]. These physonects, sister to all other codonophorans (Figure 10), also lack complex nematocyst batteries and have simple unbranched gastrozooid tentacles, and palpons with elongate palpacles indistinguishable from the tentacles. Nematocysts include birhopaloids (Figure 11) of two sizes (fig. 1 [127] and fig. 3a–d [124]), and in other species rhopaloids with a single swelling on the shaft [11]. These rhopaloid types are unique to the Apolemiidae (amongst Siphonophora) and in *A. uvaria* birhopaloids occur in pairs down the lengths of relaxed tentacles [9]. There are also two types of heteronemes in most apolemiids, including stenoteles (two size classes) and microbasic mastigophores (one size class) around the mouths of gastrozooids and palpons (Table 6), and haploneme isorhizas of two size classes on the surfaces of bracts and palpons, and probably also on the tentacles of some apolemiids from the NE Pacific [9], [11].

**Figure 12:**
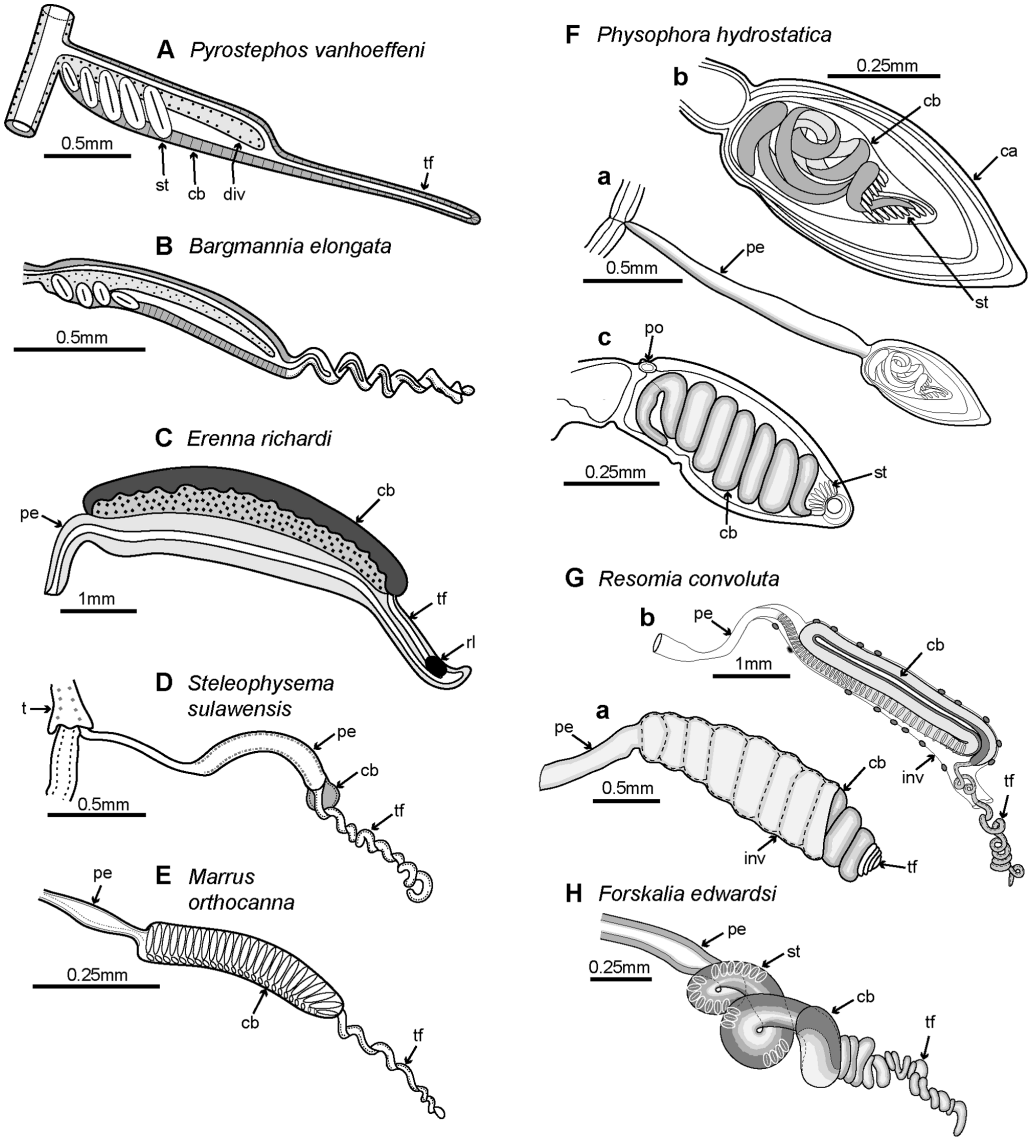
Schematic representations of tentilla of dioecious and monoecious physonect siphonophores. A: *Pyrostephos vanhoeffeni* (after fig. 44 [9]); B: *Bargmannia elongata* (after fig. 14F [6]); C: *Erenna richardi* (after fig. 7D [15]); D: *Steleophysema sulawensis* (derived from fig. 4 [89]); E: *Marrus orthocanna* (after fig. 14D [6] and partly derived from fig. 5c [143]); F: *Physophora hydrostatica* a: (after pl. 6, fig. 8 [144]); b: (after pl. 5, fig. 8 [144]); c: (after pl. 5, fig. 10 [128]); G: *Resomia convoluta* a: zigzag tentillum (derived from pl. 32, fig. 4 [33] and fig. 11L [17]); b: spiral tentillum (derived from fig. 11G [17]); H: *Forskalia edwardsi*, derived from pl. 14, fig. 4 [128]). Labels: ca – capsule; cb – cnidoband; div – diverticulum; inv – involucrum; pe – pedicel; po – pore; rl – red lure (photophore); st – stenotele; t – tentacle (with tentilla); tf – terminal filament.

The cnidomes of monoecious physonects include nematocysts on zooids other than the tentilla (see below). These likely include acrophores on the body and stenoteles around the mouth of the gonopalpons in most forskaliid species [16], white clusters of stenoteles or orange clusters of microbasic mastigophores on the tips of the enlarged palpons of *Physophora* species (Table 6, Figures 4C, 12F) and large microbasic mastigophores on the bracts of *Resomia ornicephala*, with similar nematocysts also on the palpacles and gonophores of this species, and two patches on the lateral surfaces of the nectophores [91].

#### Tentilla

In all siphonophores other than cystonects and apolemiids, the nematocysts used for feeding are contained within complex nematocyst batteries on side branches of the tentacles, here termed tentilla (for definition, see p. 74 [6]). A few other authors refer to them as nematocyst batteries or tentillar batteries [67], [141]. The appearance of these batteries during evolution coincides with the loss of large polyps from the nectosome (present in apolemiids) and a change in diet from soft-bodied prey to hard-bodied crustacean prey [67]. The batteries represent a transition during the phylogeny of Siphonophora which might perhaps have occurred after the origin of the nectosome by pro-bud subdivision and before a change in sexual state from dioecy to monoecy (see fig. 7 in [8]).

The nematocysts of such tentilla are contained within a cnidosac [6], or saccus [9], which can be simple or complex. Complex batteries are better known than simple examples, because they are characteristic of the better studied and more abundant species of physonects, for which they can be diagnostic. Each comprises a cnidoband, terminal filament(s) and elastic strands [70], [142], which together function to rapidly entangle the prey and simultaneously release the cnidoband, by a mechanism explained in more detail below. In addition, the cnidome of non-apolemiid physonect codonophorans includes nematocysts on other zooids, including bracts, nectophores, palpons or palpacles. Such nematocysts are probably for defence (as in apolemiids). It is also important to remember that during collection the tentacles and their side branches are easily torn off or shed, due to the delicate nature and sensitivity of siphonophores [43]. The cnidome is, therefore, rarely completely known for less common siphonophores, or, indeed, for many common species, although details of the cnidomes of species in the monoecious physonect family Resomiidae are given by Pugh and Haddock [91].

Dioecious physonect tentilla: The tentilla of pyrostephids and erennids differ from those of other dioecious physonects and all monoecious codonophorans in having a cnidoband of very small nematocysts and an axial gastrovascular canal which penetrates the length of the terminal filament to the tip (Figure 12A–C, Table 7). These tentilla are probably held out straight in life, and their terminal filaments have either many small nematocysts similar to those in the centre of the cnidoband (pyrostephids), or none (erennids); in the latter there is a pair of pigmented photophores (ocelli) which are held out stiffly during feeding and vibrate to act as a lure (see below).

**Table 7:** Physonect tentilla.

Family/genus	Length, shape and cnidoband details	Terminal filament(s)	References
**Dioecious:**			
Pyrostephidae	<50 mm with straight cnidoband of many small rhopalonemes,likely acrophores and desmonemes, flanked proximally by a fewlarge heteroneme stenoteles (Figure 12A–B); no involucrum	Flexible with central axial canal andcomprising many of the same smallnematocysts as in the cnidoband	[14]
Erennidae	<30 mm with straight cnidoband of many small haplonemes oftwo shapes flanked by slightly larger anisorhizas (Figure 12C);no involucrum	Stiff and with central axial canal but nonematocysts; pair of pigmentedphotophores near distal end	[15]
Rhodaliidae	<1.5 mm with loosely coiled or straight cnidoband of, whereknown, numerous anisorhizas flanked by larger heteronemes(Figure 12D); no involucrum; tentilla carried only on thetentacles of type II gastrozooids in rhodaliids	Flexible and without central axialcanal; many smallrhopaloneme nematocysts (Table 6)	[4], [27], [45], [89]
*Marrus*	<5 mm with straight or loosely coiled cnidoband of many small central haplonemes flanked by two rows of larger heteronemes (Figure 12E; Table 6); no involucrum	Flexible with a string of desmonemesand acrophores (Table 6)and no central canal	[90]
**Monoecious:**			
*Forskalia*	<2 mm with pedicel contracted; coiled orange-red cnidobandof anisorhizas and possibly some isorhizas, flanked by two rowsof large stenoteles (Figure 12H); no involucrum	Flexible with repeating pattern of onepair of desmonemes and two pairsof acrophores in *F. edwardsi* and*F. contorta*	[16], [142]
*Physophora*	<5 mm long with distal capsule enclosing inverted coiledcnidoband of many small anisorhizas flanked by a few largeyellow microbasic mastigophores at its attached distal end;cnidoband discharge via a pore at proximal end of capsule(Figure 12F, a–c).	Absent in mature tentilla	[9], [25], [128], [142], [144]
*Resomiidae*	<9 mm with cnidoband of many anisorhizas flanked by severalmicrobasic mastigophores; tentilla from proximal end oftentacle with coiled cnidoband, and from distal end withzigzagged cnidoband (Figure 12G, a–b); involucrum complete,with extra swelling from pedicel floating above cnidoband andforming a lure in *R. ornicephala*	Flexible string of desmonemesand acrophores in *R. ornicephala*	[17], [91]
*Agalma*	<4 mm with tightly coiled red cnidoband of many anisorhizasflanked proximally by microbasic mastigophores; completeinvolucrum (figure 13Aa). Larval tentilla on first tentacle only,small, with few nematocysts, long cnidocils for prey captureand no cnidoband or terminal filaments (Figure 13Ab)	Two flexible terminal filaments ofdesmonemes and acrophoresseparated by nematocyst-freeampulla in definitive tentillum	[68], [145], [147]
*Athorybia* and *Melophysa*	Similar to *Agalma*, except that in *Athorybia* there is a secondtentillum type with uncoiled cnidoband, nematocyst-freedendritic processes arising from the pedicel, with theheteronemes of *Athorybia rosacea* being stenoteles	As above except that in *Athorybia* *lucida* there is no ampulla and the twoterminal filaments are loosely fusedalong their lengths	[34], [148]
*Halistemma*	<6 mm with tightly coiled red cnidoband of many anisorhizasflanked proximally by stenoteles; very reduced involucrum(Figure 13B).	Flexible string of desmonemes andacrophores with specifically variabledistal swollen sinker (cupulate process)comprising ring of nematocysts with inertcap (*H. cupulifera*), smaller swelling (*H.* *foliacea*) or small spiral(*H. rubrum*)	[32], [92], [93], [128], [142]
*Nanomia*	<9 mm with tightly coiled cnidoband; comprising 4500anisorhizas flanked proximally by 15–35 large stenotelesin *N. bijuga,* 14000 anisorhizas flanked by 70–80 stenotelesin *N. cara*; partial involucrum (Figure 13C)	Flexible string of one or two typesof smaller desmonemes andrhopalonemes (probably acrophores)	[67], [149]
*Lychnagalma*	<7.5 mm with large complexly coiled red cnidoband of manylikely anisorhizas, flanked by two rows of larger heteronemes,probably stenoteles; complete involucrum (Figure 16C)	Eight terminal filaments surroundinga large nematocyst-free ampullawhich acts as a lure	[131]
*Cordagalma*	<0.14 mm long with retained larval tentillum in*C. ordinata* 4–7 heteronemes, 15 haplonemes (Figure 13D);definitive tentillum in *C. tottoni*	–	[67], [94], [130]
*Frillagalma*	<2 mm, unique tentillum with no cnidoband;instead a simple capsule with 3 proximalstenoteles and 30–35 distalanisorhizas (Figure 13E)	Absent; tentillum with 2 sequentialampullae only beyond the cnidosac	[97]

Note: tentillum lengths given here include cnidoband and any terminal structures and are derived from photographic images of tentilla, where available, most preserved (and therefore contracted).

In rhodaliids and species in the genus *Marrus* the mature cnidoband, where known, is often, although not always, loosely coiled, and typically comprises a suite of larger nematocysts which include many small central haplonemes (probably homotrichous anisorhizas) flanked by some large heteronemes (Table 7, Figure 12D–E). The latter may be microbasic mastigophores, or in *Thermopalia taraxacum*, stenoteles [27]. No figures have yet been published showing the arrangement of nematocysts in rhodaliid tentilla.

**Figure 13:**
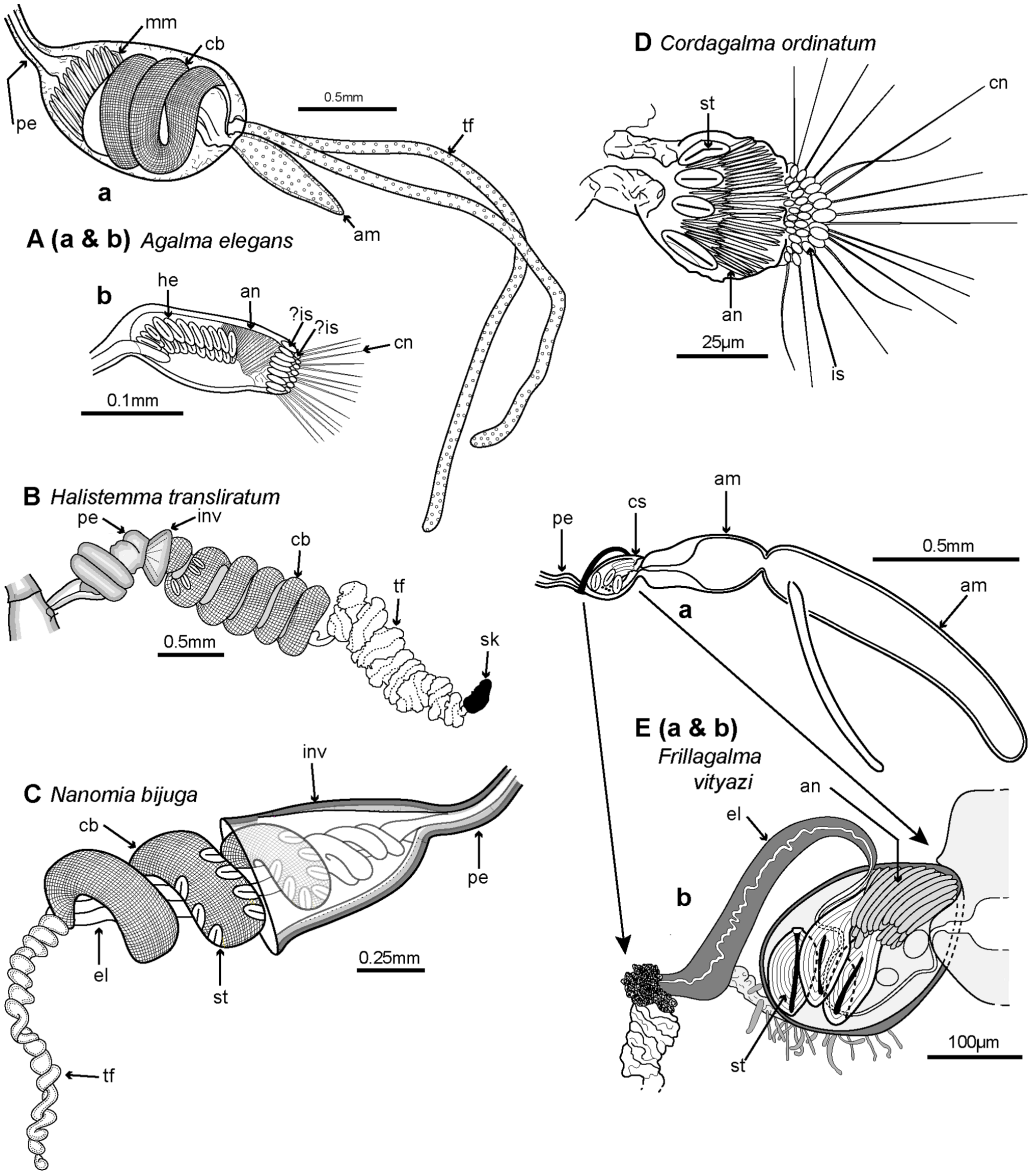
Schematic representations of tentilla from more monoecious physonect siphonophores. A: a: *Agalma elegans* (derived from pl. 7, fig. 17 [68]); b: *Agalma elegans* larval tentillum (derived from pl. 9, fig. 9 [147]); B: *Halistemma transliratum* (derived from fig. 7B [92]); C: *Nanomia bijuga* (derived from pl. 19, fig. 10 [34]); D: *Cordagalma ordinatum* (derived from pl. 3, fig. 7 [130] and pl. 15, fig. 12 [26]); E: a: *Frillagalma vityazi* (derived from fig. 6A [97]); b: cnidosac of *F. vityazi* tentillum (12a) enlarged (from fig. 7 [97]). Labels: am – ampulla; an – anisorhiza; cb – cnidoband; cn – cnidocil; cs – cnidosac; el – elastic strand; he – heteroneme; inv – involucrum; is – isorhiza (some questionable are labelled ?is); mm – microbasic mastigophore; pe – pedicel; sk – sinker; st – stenotele; tf – terminal filament.

Monoecious physonect tentilla: Monoecious species of the physonect families Forskaliidae, Physophoridae, Resomiidae and Agalmatidae *sensu stricto* typically have tightly coiled cnidobands and a single terminal filament, while tentilla of the unassigned genera *Cordagalma, Frillagalma and Lychnagalma* are more varied (Figure 12F–H, Figure 13A–E, Table 7). Cnidobands typically comprise many small homotrichous anisorhizas flanked proximally by large microbasic mastigophores or stenoteles, with the terminal filament composed of smaller desmonemes and rhopaloneme acrophores (Table 7). A thin and transparent protective involucrum partially or completely covers the cnidoband in many mature tentilla (Table 7).

Forskaliid tentilla have particularly long pedicels, a loosely coiled cnidoband without an involucrum (Figure 12H) and nematocysts as noted in Table 7. A larval-type tentillum has also been identified in one species [16]. Prey consumed is typically copepods and sometimes decapod larvae, shrimp and chaetognaths, but no ostracods or gelatinous zooplankton [140]. In physophorids, the tentilla are unusual and carried on tentacles which, when relaxed, are extremely elongate (pl. 1, fig. 1 [128]). Tentilla are similar in both *Physophora* species and of unique construction (Figure 12Fa–c) with the cnidoband becoming enclosed and inverted inside a layered capsule during maturation (Table 7). Resomiid tentacles bear two types of tentilla on each tentacle, and the cnidoband changes configuration from coiled to zigzag as it matures (Figure 12Ga–b). The transformation process is particularly well illustrated in the series of published images quoted in Table 7, and in all species but one the involucrum of the tentillum forms a transparent tube enclosing the cnidoband throughout the transformation process. Simplified larval tentilla with a short straight cnidoband have been identified on one tentacle of *Resomia ornicephala* [91].

Tentilla of Agalmatidae *sensu stricto* (*Agalma, Athorybia, Melophysa, Halistemma* and *Nanomia)* are tightly coiled in life; details of their cnidobands and terminal filaments are given in Table 7 and shown in Figure 13Aa, B–C. Larval tentilla occur only on the first tentacle [145], as in other monoecious species (see above); these tentilla are small and simple with some large heteronemes proximally, followed by small and large anisorhizas, and distally some isorhizas bearing elongate cnidocils for prey capture (Figure 13Ab). There are also microbasic euryteles (Table 6) on the larval bract of *A. elegans* [73], at the distal ends of each tentacle, and in two spots on each side of the ostium of the nectophores [146]. In *Agalma* species the tentilla are tricornuate because they have three distal structures: an ampulla and two terminal filaments (Fig. 13Aa). The terminal filament of *Halistemma* tentilla has a ‘sinker’ [142] (Figure 13B) or ‘cupulate process’ at the distal end which is specifically variable (Table 7) and similar to that found in many calycophorans (see Table 8 below). The larval bracts of *H. rubrum*, like those of *A. elegans*, contain euryteles [73].

**Table 8:** Calycophoran tentilla.

Family/species	Length, shape and cnidoband	Terminal filament	References
**Prayomorphs:**			
*Amphicaryon peltifera*	0.1 mm with short curved cnidoband of presumedanisorhizas flanked by two pairs of larger presumedmicrobasic mastigophores	Unknown	[26] as *Mitrophyes* *peltifera*
*Rosacea cymbiformis*	1.25 mm with J-shaped cnidoband of 400 anisorhizasand 25–30 microbasic mastigophores proximallyand some large desmonemes distally (Figure 14A)	Two nematocyst types:rhopalonemes alternatingwith small desmonemes	[46], [67], and as *Praya* [150]
*Stephanophyes superba*	0.7–0.9 mm with long folded over cnidoband of2000 likely anisorhizas flanked by 32–50 largerheteronemes with group of large desmonemedistally (Figure 15D)	Two nematocyst types:rhopalonemes alternatingwith small desmonemes	[67], [151]
*Nectadamas diomedeae*	2.5 mm long, straight and with unique bulb-shapeddistal end of cnidoband comprising proximalring of possible heteronemes and narrower distalrings of possible anisorhizas, with distal capof 50–70 nematocysts, maybestenoteles, with long cnidocils	Absent	[14], [38], [152]
*Hippopodius hippopus*	0.3 mm long with U-shaped cnidoband of200 anisorhizas and 7–10 microbasicmastigophores	One nematocyst type:either anacrophores orsmall desmonemes	[26] as *Polyphyes* *ungulata*, [34], [67]
*Vogtia spinosa*	1.1 mm long with twice folded red cnidoband,probably of anisorhizas and microbasicmastigophores but needs confirmation	Probably as in *Hippopodius,*but needs confirmation	[34]
**Diphyomorphs:**			
*Kephyes ovata*	0.47 mm with L-shaped cnidoband of 5+ largeheteronemes proximally and group of smallerhaplonemes (probably largedesmonemes) distally	Probably of anacrophoresand/or small desmonemes,with larger desmonemesin sinker at distal end,but needs confirmation	[25]
*Sulculeolaria*	0.6 mm mm long with slightly curved cnidoband*(S. turgida)* of 200 anisorhizas and 8 heteronemes,probably mms (*S. quadrivalvis*)	Very adhesive, with twonematocyst types and sinkerat distal end	[25], [67], [128]
*Diphyes dispar*	0.5 mm long with long and slightly curved cnidoband of 250 anisorhizas, 12 microbasic mastigophores and group of large desmonemes distally (Figure 14D)	One nematocyst typeknown; sinker at distal end	[26], [67], and as*Doramasia picta* [154]
*Dimophyes arctica*	0.8 mm long with slightly curvedcnidoband of many likely anisorhizasand c. 18 large heteronemes (more than mostother diphyine diphyids) plus distal groupof large desmonemes; all needconfirmation	Unknown	[31]
*Abylopsis tetragona*	2.2 mm long (longest known diphyomorphtentillum) with 800 haplonemes, 20–21 heteronemesand probably a distal group of largedesmonemes, though theseneed confirmation	Two nematocyst types:anacrophores and smalldesmonemes; sinker notyet identified, but may bepresent	[67], [152]
*Sphaeronectes*	0.1 mm, short, with short slightly curvedcnidoband of 50 haplonemes (anisorhizas)and 1–4 large proximal heteronemes (microbasicmastigophores in *S. haddocki*) and a group ofprominent large desmonemes distally (withlong cnidocils)	Two nematocyst types:probably anacrophoresand small desmonemes, withsinker distally in at least onespecies (*S. koellikeri*)	[18], [67]

Note: tentillum lengths are derived from published images, excluding the pedicel and including the terminal filament contracted.

Of the three monoecious genera with a ventral nectosome (named in Figure 10) only *Lychnagalma* has a tentillum similar to that of the Agalmatidae *sensu stricto* (Table 7), but it includes more terminal filaments and probably acts as a lure (see below). The other two genera have a much smaller cnidosac (except perhaps *Cordagalma tottoni*), with that of *Cordagalma ordinata* (Figure 13D) resembling the larval tentillum of *Agalma elegans* (Figure 13Ab), and that of *Frillagalma vityazi* bears two enormous sequential distal ampullae (Figure 13Ea–b). Details of these three tentilla are given in Table 7, but their affinities with other monoecious physonects are unclear.

**Figure 14:**
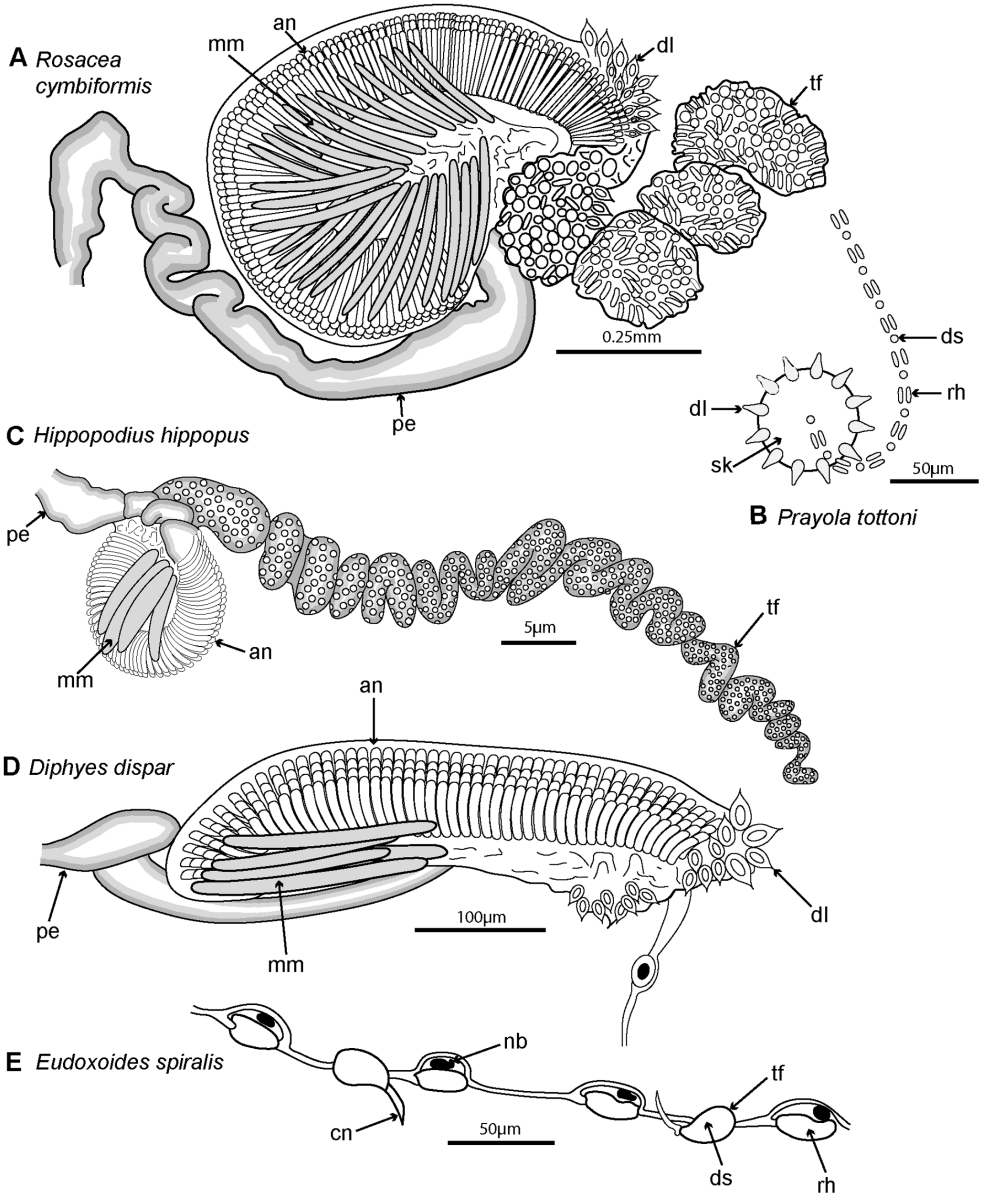
Schematic representations of tentilla from calycophoran siphonophores. A: typical prayid tentillum, *Rosacea cymbiformis* (re-drawn compilation from fig. 3E [67] and fig. 189 [150]); B: Sinker of *Prayola tottoni* (re-drawn from pl. 1, fig. 3 [133]); C: typical hippopodiid tentillum, *Hippopodius hippopus* (re-drawn from fig. 3C [67]); D: typical diphyomorph tentillum, *Diphyes dispar (*re-drawn from fig. 3I [67]); E: Detail of extended terminal filament of *Eudoxoides spiralis* (re-drawn from fig. 112 [122]). Labels: an – anisorhiza, cn – cnidocil; dl – large desmoneme; ds – small desmoneme; mm – microbasic mastigophore; nb – nematoblast; pe – pedicel; rh – rhopaloneme; sk – sinker; tf – terminal filament.

**Figure 15:**
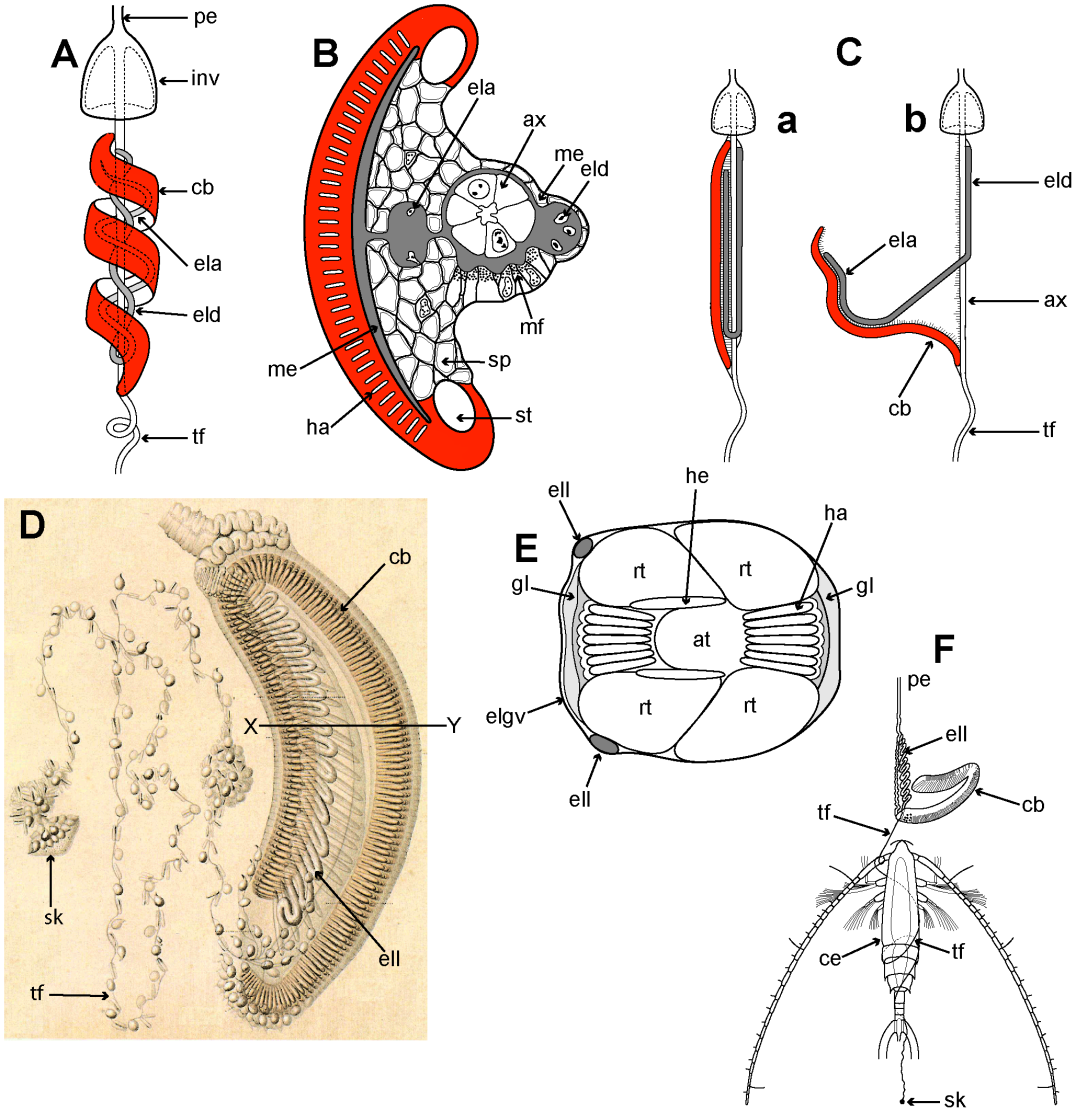
Tentillum discharge in siphonophores. A-C: *Nanomia bijuga* (redrawn from [155], A: fig. 2, schematic of undischarged tentillum; B: fig. 3, schematic section through tentillum; Ca & b: fig. 4a & b, schematic of tentillum discharge; D–E: *Stephanophyes superba* (after [151]); D: pl. 4, fig. 4, undischarged tentillum; E: txt fig. 3, schematic section through tentillum at X-Y; F: txt fig. 4, discharged tentillum with captured copepod. Labels: at – artefact (not a natural cavity); ax – axis/axial canal (endodermal); cb – cnidoband; ce – entangled copepod; ela – ascending elastic strand; eld – descending elastic strand; elgv – ectodermal lamella with red gastrovascular cells; ell – looped elastic strand; gl – glandular cells; ha – haploneme; he – heteroneme; inv – involucrum; me – mesogloea; mf – muscle fibres (in ectoderm); pe – pedicel; rt – reticulate (supporting) cell; sk – sinker; sp – spongy ectoderm; st – stenotele; tf – terminal filament.

Calycophoran tentilla: Calycophorans are monoecious (see Figure 9) with tentilla mostly of uniform design, arising from more numerous and closely spaced tentacles than those of physonects. Calycophoran tentilla are laterally compressed with U-shaped, folded or relatively straight cnidobands, and a long terminal filament (Figure 14A–C, Figure 15D). Often, there is a swelling, or sinker [142], at the distal end of the terminal filament, which bears a ring of large desmonemes, and acts as a weight to hold down the fine terminal filament during feeding (Figure 14B, 15F). Cnidobands typically comprise many small anisorhizas flanked proximally by some large microbasic mastigophores (exceptionally stenoteles or euryteles), and with one or more tufts of desmonemes at the distal end (Table 6 and Figure 14A, B, D). The terminal filament contains alternating small desmonemes and rhopaloneme anacrophores, and the desmonemes of most calycophoran tentilla bear conspicuous cnidocils for prey capture (Figure 14E).

Amongst the prayids, only a single amphicaryonine tentillum has so far been figured in the literature (Table 8), although there are numerous published illustrations available of prayine tentilla from a range of species (figs. 5B, 8C, 12D [19]; fig. 3E [67]; pl. 3, fig. 5 [46]; pl. 2, figs. 3–4 [133]; pl. 1, fig. 5 [132]; pl. 3, fig. 1 [134]), all similar to that shown for *Rosacea cymbiformis* in Figure 14A. These tentilla probably all have a sinker at the distal end of the terminal filament, as described in *R. cymbiformis* (p. 157 [38]), *Stephanophyes superba* (Figure 15D; [151]) and other prayines [132], [133]), although not always evident in published figures due to contraction. Nectopyramidine prayids have either a relatively conventional tentillum (*Nectopyramis*) or a unique club-shaped type (*Nectadamas*), as noted in Table 8. Tentilla of the two genera of prayomorphs in the family Hippopodiidae may reflect their separate clades as shown in Figure 9, since the cnidoband of *Hippopodius* is short and U-shaped (Figure 14C) while that of *Vogtia* is much longer and folded (see Table 8 and fig. 86 [152]; pl. 4, fig. 7 [153]). Clausophyid, sulculeolariine, diphyine and abylid tentilla are all of similar design with details and references to published figures included for a range of species in Table 8. The cnidoband of sphaeronectid diphyomorphs, however, is relatively short, although overall tentillum structure is the same, and a sinker is figured on the terminal filament of one species (Table 8; [18]).

#### Tentillum discharge

Eruption of siphonophore tentilla is an explosive process studied only once in recent times, by Mackie and Marx [155] in the small physonect *Nanomia bijuga* (then thought to be *N. cara*). A looped elastic strand of mesogloeal origin extends distally inside the tentillum from the pedicel to the origin of the terminal filament (Figure 15A–C), and plays an important role in tentillum discharge; it allows the cnidoband to slap onto the prey whilst still remaining attached to the pedicel. A descending portion of the elastic strand spirals around the axial endodermal gastrovascular canal, while an ascending portion passes back up on the inside surface of the cnidoband (Figure 15Ca). A transverse section through the tentillum (Figure 15B) shows how the prominent ectodermal cnidoband composed of haploneme and heteroneme nematocysts is backed by a sheet of mesogloea, and connected to the axial canal by spongy supporting ectodermal tissue. This tissue extends around both portions of the elastic strand and the axial gastrovascular canal, and the mesogloea from the cnidoband penetrates into it, first thickening to form the ascending strand, continuing on around the axial gastrovascular canal, and thickening again to form the descending strand (Figure 15B). Mesogloea around the axial canal is asymmetrically thickened to support strands of longitudinal ectodermal muscles (Figure 15B), and adjacent to these is a bundle of nerve cells (fig. 6E [154]).

During feeding, the terminal filament relaxes and extends well beyond the tentillum (fig. 1A [155]) to ensnare unsuspecting prey. Pulling down on the filament causes eruption of the cnidoband, which tears the spongy ectodermal tissue as it uncoils. After eruption, Mackie and Marx [155] find that the elastic strand remains firmly attached to the axial canal at its proximal end and to the cnidoband at its distal end (Figure 15F, ‘cb’); attachment is enhanced in physonects by phosphatic spicules [155]. The struggling prey is brought to the cnidoband by contraction of the terminal filament, and rapidly stunned by multiple discharge of the haploneme and heteroneme nematocysts of the cnidoband. The tentacle then contracts, bringing the prey to the gastrozooid for ingestion and digestion. Longitudinal muscle fibres also contribute to tentillum discharge (Figure 15B, mf), and are thought by Mackie and Marx [155] to be under nervous control.

Tentillum discharge in calycophorans has not been studied since the work of Chun in the 1890s [31], [151], [154], who describes it in great detail in 1891 for the prayine prayid *Stephanophyes superba*. The mature cnidoband of this species is folded over into a deep inverted J-shape (Figure 15D), supported by four giant reticulate cells, and best seen in a section through the tentillum (Figure 15E) taken along the line x-y of Figure 15D. This section passes twice through the cnidoband, with the axial gastrovascular canal and left and right portions of the elastic strand passing to the terminal filament along one side of the cnidoband only (left side in Figure 15D); both portions are firmly attached to the pedicel, unlike *N. bijuga*, and there are no phosphatic spicules, as noted above. The anisorhizas of *S. superba* are covered with a fenestrated mesogloeal membrane (Figure 15E) which is ripped off as they discharge (see txt fig. 5 [151]). Also, in *S. superba*, as in *Enneagonum hyalinum* (as *Halopyramis adamantina* Chun 1892) and probably other calycophorans, once the tentilla are mature, the axial gastrovascular canal degenerates into a series of red cells supported by an ectodermal lamella which forms a thin layer on the external surface of the shorter limb of the cnidoband (Figure 15E, elgv).

When a copepod becomes entangled in the terminal filament of *Stephanophyes superba* and pulls down on it, the cnidoband escapes from the sling of the looped elastic strand and flicks out rapidly onto the prey, unfolding as it does so (Figure 15F). Contraction of the terminal filament brings the prey up to the cnidoband and the cnidoband nematocysts discharge simultaneously, tearing the fenestrated membrane as shown by Chun (txt fig. 5 [151]). The convoluted elastic strand gives slack to the system [70], and firmly connects the struggling prey to the pedicel. Once stunned, the captured prey is brought to the gastrozooid as described above. Tentilla of the diphyomorph *Dimophyes arctica* discharge in a similar way [31], and the elastic strand of the abylid *Enneagonum hyalinum* extends into a much longer and stronger rope-like structure of two intertwined branches, that is itself sinuously folded in a sheet of thickened mesogloea (pl. 12, fig. 16 [154]). This may allow more efficient prey capture than in *S. superba*, but further study is needed to gain a better understanding of the structure and functioning of these fascinating feeding organs.

**Figure 16:**
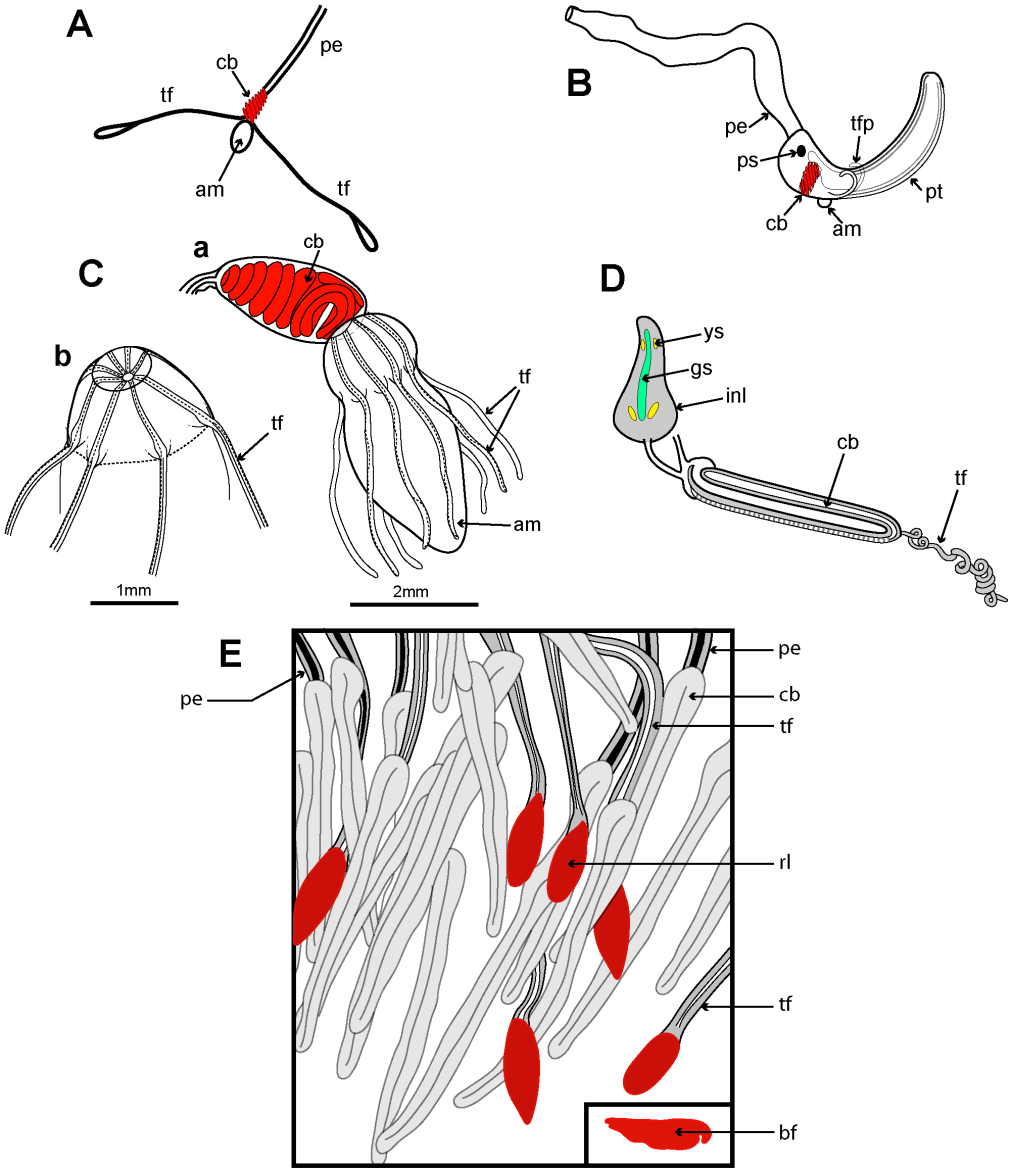
Lures in some physonect siphonophores. A: *Agalma okeni* copepod mimic lure (after fig. 2A [157]); B: *Athorybia rosacea* dendritic tentillum fish larva mimic lure (adapted from fig. 2B [157], fig. 46B [2] and pl. 1, fig. 8 [158]); C: a: *Lychnagalma utricularia* hydromedusa mimic lure (redrawn from fig. 5B [131], in part); b: proximal end of detached ampulla mimicking hydromedusa radial canals (redrawn from fig. 5A [131]); D: *Resomia ornicephala* fluorescent involucral lure (after figure in table 6 [91]); E: Red fluorescent lures on tentilla of *Erenna* sp. (redrawn from internet image by Steven Haddock 2004 © MBARI). Labels: am – ampulla; bf – red barbelet fish (the likely prey of this *Erenna* species); cb – cnidoband; gs – green stripe; inl – involucral lure; pe – pedicel; ps – pigment spot (mimics fish eye); pt – pendant growth (mimics fish body); rl – red lure (photophore); tf – terminal filament; tfp – terminal filament (mimics fish pectoral fin); ys – yellow spot(s).

Electrical signals are propagated through the tentillum of *Nanomia bijuga* from the pedicel to the terminal filament by the axial bundle of neurones described above. Although Mackie and Marx [155] were unable to implicate any neuromuscular mechanisms in the eruption process, it seems likely that the axial muscles are under nervous control, as in the tentacle, and may bring about an increase in hydrostatic pressure in the axial canal which causes eruption. A recent study of the nematocyst batteries of *Hydra magnipapillata*, another hydrozoan, has shown its neurones to be both light sensitive and connected to the nematocysts of the battery by short processes [156]. Genes have been identified in this species of *Hydra* which are expressed in the battery neurone as an opsin transduction cascade. Bright light is found to inhibit this cascade and also the discharge of nematocysts, whereas in dim light nematocysts are reactive. Plachetzki et al. [156] suggest that light sensitivity might be widespread in the neurones of hydrozoans, possibly triggering diurnal migration in pelagic species and limiting all-or-nothing nematocyst discharge to conditions of high prey abundance, which would conserve energy. Confirmation of these genes in siphonophore species which are known to undergo diurnal vertical migration [2] would make an interesting project, and a useful contribution to siphonophore ecology.

#### Lures

Smaller fast moving siphonophores, such as diphyids, spread their tentacular webs out rapidly, and move quickly to new areas when prey is scarce. Larger siphonophores are less mobile and conserve energy by extending their feeding webs and lying in wait for prey [3]. Such species are ambush predators, and some have tentilla which may be modified into lures to attract prey [157]. Tentilla of the physonect *Agalma okeni* resemble small copepods with the body being represented by the red cnidoband and the antennae by the two terminal filaments (Figure 16A). Predators of small copepods are attracted to the tentilla, including crab megalopa larvae, large copepods and euphausids, which have all been identified in the gastrozooids of *A. okeni* [157]. Other *Agalma* species have similar tentilla that may also act as lures. Species of the genera *Athorybia* and *Melophysa* have small tentilla with two terminal filaments which, during feeding, are constantly jigged through the water resembling the jerky swimming movements of copepods. The white tentilla of *Physophora hydrostatica* are moved in a similar fashion, suggesting that they too might act as lures [129].

*Athorybia* species have a second type of tentillum on some tentacles which are more elongate with various types of tree-like outgrowths [9]. In *A. rosacea* these dendritic tentilla resemble fish larvae [157], with a pendant growth from the involucrum for the body, two curled terminal filaments for the pectoral fins and two pigment spots proximal of the cnidoband for the eyes (Figure 16B). These tentilla also move in a manner similar to a swimming fish larva, by alternating bouts of two to three contractions with a pause [157]. Fish larvae are attracted, together with predatory chaetognaths that consume the larvae, since both were found in the gastrozooids of *A. rosacea* [157]. In *A. lucida* the dendritic growths of these tentilla are differently shaped (see fig. 4 [148]), and may resemble larvacean housings [157].

One unusual physonect *Lychnagalma utricularia* has tentilla with eight terminal filaments surrounding a swollen central ampulla, and may mimic a small swimming hydromedusa [131] (Figure 16C). The tentilla contract periodically and their terminal filaments are very extensible. Unfortunately, no prey items were found by Pugh and Harbison [131] in the gastrozooids, and none reported since for this species.

Recently, two different physonect species have been reported to use lures producing light to attract their prey, an unusual phenomenon amongst gelatinous cnidarians [159]. The first is *Resomia ornicephala* which, by day, inhabits a narrow depth range of circa 200 m off California, feeding mainly on euphausiid shrimp (krill), and competing with another physonect *Nanomia bijuga* for available prey; its success may be due to a luminous lure. Each tentillum develops an outgrowth from the involucrum with a pattern of green and yellow pigments which resembles a bird’s head (Figure 16D). Illumination by the ambient blue light at this depth excites the pigments to fluoresce, and also, more weakly, the cnidoband (fig. 7A [91]). Either the silhouette produced by these lures, or the pattern of fluorescent pigments each involucrum contains provides an appealing silhouette to predatory euphausiids such as *Thysanoessa*; these shrimp swim into the tentilla and are captured [91].

The most exciting discovery in recent years is the identification of a red bioluminescent lure in a new species of *Erenna* from the deep sea [12]. Bioluminescence is widespread amongst marine taxa, and is exhibited in different forms by 91% of siphonophores (fig. 2b [160]), although for most species the main function of bioluminescence seems to be defence. In hydrozoans this process is catalysed by a photoprotein which, on addition of calcium ions, causes the coelenterazine substrate (a type of luciferin, see [160] for further details) to become incorporated within it and to emit a photon of light. Thus, the reaction is not dependent upon free oxygen as in some bioluminescent taxa, and it has the advantage of conferring considerable control over the emission of light by the organism. The first photoprotein to be extracted was aequorin, from the hydromedusan *Aequorea victoria*, where it is localized around the margin of the bell, and associated with another macromolecule, green fluorescent protein [161]. Fluorescent proteins, however, emit light only when excited, as in the *Resomia* species described above, whereas in the bioluminescent lures of *Erenna*, light is generated internally.

The species of *Erenna* with red lures was observed in deep water between 1600 and 2300 m in the eastern Pacific, off the west coast of California, and lives in total darkness, where prey is scarce. Haddock and colleagues [12] discovered that *Erenna* has evolved the remarkable ability to produce red light from photophores (or ocelli), a property almost unknown among other marine invertebrates. The photophores are located near the distal ends of the terminal process of each tentillum (Figure 12E, rl). Cells in the core of each photophore are bioluminescent and emit blue-white light. Young tentilla near the proximal end of the tentacle emit white light, and then, as each tentillum matures, a layer of tissue containing a red fluorescent protein grows around this core which modulates the light emitted into longer wavelength red light (Figure 16E). The tentacles of *Erenna* are never extended, but instead held close to the body and oscillated rhythmically during feeding to attract prey. Haddock et al. [12], [162] suggest this movement mimics the swimming of small copepods which might be the prey of the red barbelet, a small deep-sea fish of the genus *Cyclothone*. The latter could be the most common fish in the ocean, although hardly ever found or studied due to the difficulty of sampling at such great depths. If red light is indeed detected by these fish and they swim into a swarm of so-called ‘copepods’, then they themselves are likely to fall victim to the tentilla of this particular *Erenna* species. Pigmented lures are also present on the rigid terminal processes of *E. richardi* and *E. laciniata* [15], which are thought to attract prey in a similar way, although not always in such deep water as *Erenna* species.

## References

[1] WoRMS Siphonophora List. World Register of Marine Species website. Available: http://www.marinespecies.org/aphia.php?p=taxdetails&id=1371. Accessed 2014 Jan 6.

[2] Mackie GO, Pugh PR, Purcell JE (1987) Siphonophore Biology. Adv Mar Biol 24: 97–262.

[3] Robison BH (2004) Deep pelagic biology. J Exp Mar Biol Ecol 300: 253–272.

[4] Mapstone GM, Ljubenkov JC (2013) New observations on *Dromalia alexandri* Bigelow, 1911, a rhodaliid physonect siphonophore from southern Californian waters. Marine Ecology 34 (1): 96–112.

[5] Alvariño A (1971) Siphonophores of the Pacific with a review of the world distribution. Bull Scripps Inst Oceanogr Univ Calif Technical Series No. 16.

[6] Mapstone GM (2009) Siphonophora (Cnidaria: Hydrozoa) of Canadian Pacific waters. Ottawa, Ontario, Canada: NRC Research Press. 302 p.

[7] Haddock SHD, Dunn CD, Pugh PR (2005) A re-examination of siphonophore terminology and morphology, applied to the description of two new prayine species with remarkable bio-optical properties. J Mar Biol Assoc UK 85 (3): 695–707.

[8] Dunn CW, Wagner GP (2006) The evolution of colony-level development in the Siphonophora (Cnidaria: Hydrozoa). Dev Genes Evol 216 (12): 743–75. 10.1007/s00427-006-0101-8 16983540

[9] Totton AK (1965) A Synopsis of the Siphonophora. London: Trustees of the British Museum (Natural History). 230 p.

[10] Dunn CW, Pugh PR, Haddock SHD (2005) Molecular phylogenetics of the Siphonophora (Cnidaria), with implications for the evolution of functional specialisation. Syst Biol 54 (6): 916–935. 10.1080/10635150500354837 16338764

[11] Siebert S, Pugh PR, Haddock SHD, Dunn CW (2013) Re-evaluation of characters in Apolemiidae (Siphonophorae) with description of two new species from Monterey Bay, California. Zootaxa 3702 (3): 201–232. 10.11646/zootaxa.3702.3.1 26146720

[12] Haddock SHD, Dunn CW, Pugh PR, Schnitzler CE (2005) Bioluminescent and red-fluorescent lures in a deep-sea siphonophore. Science 309: 263. 16002609 10.1126/science.1110441

[13] Pugh PR (1992) A revision of the sub-family Nectopyramidinae (Siphonophora, Prayidae). Philos Trans R Soc Lon B Biol Sci 335: 281–322.

[14] Pugh PR (1999) A review of the genus *Bargmannia* Totton, 1954 (Siphonophorae, Physonecta, Pyrostephidae). Bull Nat Hist Mus Zool Ser 65 (1): 51–72.

[15] Pugh PR (2001) A review of the genus *Erenna* Bedot, 1904 (Siphonophora, Physonectae). Bull Nat Hist Mus Zool Ser 67 (2): 169–182.

[16] Pugh PR (2003) A revision of the family Forskaliidae (Siphonophora, Physonectae). Nat Hist 37: 1281–1327.

[17] Pugh PR (2006) The taxonomic status of the genus *Moseria* (Siphonophora, Physonectae). Zootaxa 1343: 1–42.

[18] Pugh PR (2009) A review of the family Sphaeronectidae (Class Hydrozoa, Order Siphonophora), with the description of three new species. Zootaxa 2147: 1–48.

[19] Pugh PR, Harbison GR (1987) Three new species of prayine siphonophore (Calycophorae, Prayidae) collected by a submersible, with notes on related species. Bull Mar Sci 41 (1): 68–91.

[20] Eschscholtz F (1829) System der Acalephen. Eine ausführliche Beschreibung aller Medusenartigen Strahlthiere. Berlin: Ferdinand Dümmler. 190 p.

[21] Quoy JRC, Gaimard JP (1827) Observations zoologiques faites à bord de l’Astrolabe, en mai 1826, dans le Détroit de Gibraltar. Annls Sci nat (Series 1) 10: 1–21, 172–193; Atlas 10: pls 1–2, 4–9.

[22] Quoy JRC, Gaimard JP (1833) Zoologie. IV. In : Tastu, J. editor. Voyage de découvertes de l’Astrolabe exécuté par ordre du Roi, pendant les années 1826–1827–1828–1829, sous le commandement de M.J. Dumont D’Urville. Paris: J. Tastu. 1–390 [Text], 1–26 [Atlas].

[23] Huxley TH (1859) The Oceanic Hydrozoa; a description of the Calycophoridae and Physophoridae observed during the voyage of HMS “Rattlesnake” in the years 1846–1850. London: Ray Society, 143 p.

[24] Keferstein W, Ehlers E (1860) Auszug aus den Beobachtungen über die Siphonophoren von Neapel und Messina angestellt im Winter 1859–60. Nachr Ges Wiss Göttingen 23: 254–262.

[25] Keferstein W, Ehlers E (1861) Beobachtungen über die Siphonophoren von Neapel und Messina. Zoologische Beiträge gesammelt im Winter 1859/60 in Neapel und Messina. Leipzig: Wilhelm Engelmann, 435 p.

[26] Haeckel E (1888) Report on the Siphonophorae collected by HMS Challenger during the years 1873–1876. Report on the scientific results of the voyage of HMS Challenger during the years 1873–76. Zoology Report Volume 28. London: Her Majesty’s Government, 380 p.

[27] Pugh PR (1983) Benthic siphonophores: a review of the family Rhodaliidae (Siphonophora, Physonectae). Philos Trans R Soc Lon B Biol Sci 301: 165–300.

[28] Winsor MP (1971/2) A historical consideration of the siphonophores. Proc R Soc Edinb Biol: 73 (31): 315–323.

[29] Chun C (1886) Uber Bau und Entwickelung der Siphonophoren. Sitzungsber preuss Akad Wiss for 1886: 681–688.

[30] Chun C (1888) Berichte über eine nach den Canarischen Inseln im Winter 1887/88 ausgeführte Reise. Sitzungsber preuss Akad Wiss for 1888: 1141–1173 (English translation in Ann Mag Nat Hist Ser 6, 3 (23): 214–246, 1889)..

[31] Chun C (1897) Die Siphonophoren der Plankton-Expedition. Band II. Ergebnisse der Plankton-Expedition der Humboldt-Stiftung. Kiel & Leipzig: Lipsius and Tischer. 126 p.

[32] Lens AD, van Riemsdijk T (1908) The Siphonophora of the “Siboga” Expedition. Siboga-Expeditie 9: 1–130.

[33] Moser F (1925) Die Siphonophoren der Deutschen Südpolar-Expedition 1901–1903. Deutsche Sudpolar-Expedition 17. Zoologie 9: 1–541 [Text], 1–35 [Plates].

[34] Bigelow HB (1911) Reports on the scientific results of the expedition to the eastern tropical Pacific, in charge of Alexander Agassiz, by the U. S. Fish Commission Steamer “Albatross”, from October, 1904, to March, 1905, Lieut. -Commander L. M. Garrett, U. S. N., commanding. XXIII. The Siphonophorae. Mem Mus Comp Zool 38 (2): 173–401, 32 pls.

[35] Bigelow HB (1911) Biscayan plankton collected during a cruise of H. M. S. “Research”. 1900. Part XII. The Siphonophora. Trans Linnean Soc Lond Ser 2 Zoology 10 (10): 337–358.

[36] Robson EA (1973) Arthur Knyvett Totton, Obituary. Nature 244 (5412): 187–188.

[37] Totton AK (1932) Siphonophora. Scient Rep Gt Barrier Reef Exped 4 (10): 317–374.

[38] Totton AK (1954) Siphonophora of the Indian Ocean together with systematic and biological notes on related specimens from other oceans. Discovery Reports 27: 1–162.

[39] Totton AK (1960) Studies on *Physalia physalis* (L.). Part I. Natural history and morphology. Discovery Reports 30: 301–367.

[40] Carré C, Carré D (1991) A complete life cycle of the calycophoran siphonophore *Muggiaea kochi* (Will) in the laboratory, under different temperature conditions: ecological implications. Philos Trans R Soc Lon B Biol Sci 334: 27–32.

[41] Carré C (1979) Sur le genre *Sulculeolaria* Blainville, 1834 (Siphonophora, Calycophorae, Diphyidae). Annales de l’Institut Oceanographique 55 (1): 27–48.

[42] Pugh PR, Pagès F (1993) A new species of *Clausophyes* (Siphonophorae, Clausophyidae), with a redescription of *C. galeata* and *C. moserae* . J Mar Biol Assoc UK 73 (3): 595–608.

[43] Pugh PR (1989) Gelatinous zooplankton - the forgotten fauna. Prog Underwat Sci 14: 67–78.

[44] Pugh PR, Youngbluth MJ (1988) Two new species of prayine siphonophore (Calycophorae, Prayidae) collected by the submersibles *Johnson-Sea-Link I* and *II* . J Plankton Res 10 (4): 637–657.

[45] Hissmann K, Schauer J, Pugh PR (1995) *Archangelopsis jagoa*, a new species of benthic siphonophore (Physonectae, Rhodaliidae) collected by submersible in the Red Sea. Oceanologica Acta 18 (6): 671–680.

[46] Biggs DC, Pugh PR, Carré C (1978) *Rosacea flaccida* n. sp., a new species of siphonophore (Calycophorae Prayinae) from the North Atlantic Ocean. Beaufortia 27: 207–218.

[47] Pagès F, Pugh PR (2002) Fuseudoxid: the elusive sexual stage of the calycophoran siphonophore *Crystallophyes amygdalina* (Clausophyidae: Crystallophyinae). Acta Zool 83: 329–336.

[48] Linnaeus C (1758) Systema Naturae. Edition 10. Stockholm: Holmiae (Salvius). 823 p.

[49] Leloup E (1929) Recherches sur l’anatomie et le développement de *Velella spirans* Forsk. Arch Biol (Liege) 39: 397–478.

[50] Garstang W (1946) The morphology and relations of the Siphonophora. Q J Microsc Sci 87 (2): 103–193.

[51] Mackie GO (1959) The evolution of the Chondrophora (Siphonophora-Disconanthae): New evidence from behavioural studies. Trans R Soc Can 53 (Series 3) (5): 7–20.

[52] Kirkpatrick PA, Pugh PR (1984) Siphonophores and velellids. In: Kermack DM, Barnes RSK, editors. Synopses of the British Fauna (New Series), Number 29. Leiden: E. J. Brill, 154 p.

[53] Pagès F, Gili JM, Bouillon J (1992) Medusae (Hydrozoa, Scyphozoa, Cubozoa) of the Benguela Current (southeastern Atlantic). Sci Marina 56 (Suppl. 1)1–64.

[54] Collins AG (2002) Phylogeny of Medusozoa and the evolution of cnidarian life cycles. J Evol Biol 15: 418–432.

[55] Cartwright P, Evans NM, Dunn CW, Marques AC, Miglietta MP, et al (2008) Phylogenetics of Hydroidolina (Hydrozoa: Cnidaria). J Mar Biol Assoc UK 88: 1663–1672.

[56] Nawrocki AM, Schuchert P, Cartwright P (2010) Phylogenetics and evolution of Capitata (Cnidaria: Hydrozoa), and the systematics of Corynidae. Zool Scr 39 (3): 290–304.

[57] Petersen KJ, Cotton JA, Gehling JG, Pisani D (2008) The Ediacaran emergence of bilaterians: congruence between the genetic and the geological fossil records. Philos Trans R Soc Lon B Biol Sci 363: 1435–1443. 18192191 10.1098/rstb.2007.2233 PMC2614224

[58] Kayal E, Roure B, Philippe H, Collins AG, Lavrov DV (2013) Cnidarian phylogenetic relationships as revealed by mitogenomics. BMC Evol Biol 13: 5. 23302374 10.1186/1471-2148-13-5 PMC3598815

[59] Daly M, Brugler MR, Cartwright P, Collins AG, Dawson MN, et al (2007) The phylum Cnidaria: A review of phylogenetic patterns and diversity 300 years after Linnaeus. Zootaxa 1668: 127–182.

[60] Steele RE, David CN, Technau U (2011) A genomic view of 500 million years of cnidarian evolution. Trends Genet 27 (1): 7–13. 10.1016/j.tig.2010.10.002 PMC3058326 21047698

[61] Collins AG, Schuchert P, Marques AC, Jankowski T, Medina M, et al (2006) Medusozoan phylogeny and character evolution clarified by new large and small subunit rDNA data and an assessment of the utility of phylogenetic mixture models. Syst Biol 55 (1): 97–115. 10.1080/10635150500433615 16507527

[62] Collins AG (2009) Recent insights into cnidarian phylogeny. Smithsonian contributions to the marine sciences 38: 139–149.

[63] Gibbons MJ, Janson LA, Ismail A, Samaii T (2010) Life cycle strategy, species richness and distribution in marine Hydrozoa (Cnidaria: Medusozoa). J Biogeogr 37: 441–448.

[64] Gibbons MJ, Buecher E, Thibault-Botha D, Helm RR (2010) Patterns in marine hydrozoan richness and biogeography around southern Africa: implications of life cycle strategy. J Biogeogr 37: 606–616.

[65] Angel MV (1993) Biodiversity in the pelagic ocean. Conserv Biol Ser 7(4): 760–772.

[66] Kawamura T (1910) “Bozunira” and “Katsuwo no Eboshi” *Rhizophysa* and *Physalia*. Zool Mag (Tokyo) (Dobuts Zhasshi) 22: 445–454, 1910. (In Japanese, with English translation available).

[67] Purcell JE (1984) The functions of nematocysts in prey capture by epipelagic siphonophores (Coelenterata, Hydrozoa). Biol Bull 166 (2): 310–327.

[68] Kawamura T (1911) “Shidarezakura Kurage” and “Nagayoraku Kurage” (*Cupulita picta*, Metschnikoff, and *Agalmopsis elegans*, Sars). Zool Mag (Tokyo) (Dobuts Zhasshi) 23 (7): 359–363, plate 7 (In Japanese, with English translation available).

[69] Kölliker A (1853) Die schwimmpolypen oder Siphonophoren von Messina. Leipzig: Wilhelm Engelmann, 96 p.

[70] Mackie GO (1999) Coelenterate organs. Mar Freshw Behav Physiol 32: 113–127.

[71] Carré D (1967) Étude du développement larvaire de deux siphonophores: *Lensia conoidea* (calycophore) et *Forskalia edwardsi* (physonecte). Cah Biol Marine 8: 233–251.

[72] Carré D (1969) Étude histologique du développement de *Nanomia bijuga* (Chiaje, 1841), siphonophore physonecte, Agalmidae. Cah Biol Marine 10: 325–341.

[73] Carré D (1971) Étude du développement d’*Halistemma rubrum* (Vogt, 1852) siphonophore physonecte Agalmidae. Cah Biol Marine 12: 77–93.

[74] Pickwell GV, Barham EG, Wilton JW (1964) Carbon monoxide production by a bathypelagic siphonophore. Science 144: 860–862. 17733619 10.1126/science.144.3620.860

[75] Bidigare RR, Biggs DC (1980) The role of sulfate exclusion in buoyancy maintenance by siphonophores and other oceanic gelatinous zooplankton. Comp Biochem Physiol 66A: 467–471.

[76] Mackie GO (1964) Analysis of locomotion in a siphonophore colony. Proceedings of the Royal Society B: Biological Sciences 159: 366–391.

[77] Pugh PR (2005) A new species of *Physophora* (Siphonophora: Physonectae: Physophoridae) from the North Atlantic, with comments on related species. Syst Biodivers 2 (3): 251–270.

[78] Richter W (1907) Die Entwickelung der Gonophoren einiger Siphonophoren. Z Wiss Zool 86: 557–618, pls. 27–29.

[79] Ortman BD, Bucklin A, Pagès F, Youngbluth M (2010) DNA Barcoding the Medusozoa using mtCOI. Deep Sea Res Part 2 Top Stud Oceanogr 57: 2148–2156.

[80] Dunn CW (2005) Complex colony-level organization of the deep-sea siphonophore *Bargmannia elongata* (Cnidaria, Hydrozoa) is directionally asymmetric and arises by the subdivision of pro-buds. Dev Dyn 234: 835–845. 15986453 10.1002/dvdy.20483

[81] Bardi J, Marques AC (2007) Taxonomic redescription of the Portuguese man-of-war, *Physalia physalis* (Cnidaria, Hydrozoa, Siphonophorae, Cystonectae) from Brazil. Iheringia (Série zoologia) 97 (4): 425–433.

[82] Pagès F, Gili JM (1992) Siphonophores (Cnidaria, Hydrozoa) of the Benguela Current (southeastern Atlantic). Sci Marina 56 (Suppl. 1)65–112.

[83] Båmstedt U, Fosså JH, Martinussen MB, Fosshagen A (1998) Mass occurrence of the physonect siphonophore *Apolemia uvaria* (Lesueur) in Norwegian waters. Sarsia 83 (1): 79–85.

[84] Lindsay DJ, Miyake H (2009) A checklist of midwater cnidarians and ctenophores from Japanese waters: species sampled during submersible surveys from 1993–2008 with notes on their taxonomy. Kayo Monthly 41: 417–438 (In Japanese with English abstract)..

[85] Mapstone GM (2003) Redescriptions of two physonect siphonophores, *Apolemia uvaria* (Lesueur, 1815) and *Tottonia contorta* Margulis, 1976, with comments on a third species *Ramosia vitiazi* Stepanjants, 1967 (Cnidaria: Hydrozoa: Apolemiidae). Syst Biodivers 1 (2): 181–212.

[86] Mapstone GM (1998) *Bargmannia lata*, an undescribed species of physonect siphonophore (Cnidaria, Hydrozoa) from Canadian Pacific waters. *In* Commemorative volume for the 80th birthday of Willem Vervoort in 1997. Zool Verh (Leiden) 323: 141–147.

[87] Pagès F, Gili JM (1989) Siphonophores (Cnidaria, Hydrozoa) collected during the “Magga Dan” Expedition (1966–67) from Africa to Antarctica. Sci Marina 53 (1): 53–57.

[88] Grossmann MM, Lindsay DJ, Fuentes V (2013) A redescription of the post-larval physonect siphonophore stage known as *Mica micula* Margulis, 1982, from Antarctica, with notes on its distribution and identity. Marine Ecology 34 (1): 63–70.

[89] Hissmann K (2005) *In situ* observations on benthic siphonophores (Physonectae: Rhodaliidae) and descriptions of three new species from Indonesia and South Africa. Syst Biodivers 2 (3): 223–249.

[90] Dunn CW, Pugh PR, Haddock SHD (2005) *Marrus claudanielis*, a new species of deep-sea physonect siphonophore (Siphonophora, Physonectae). Bull Mar Sci 76 (3): 699–714.

[91] Pugh PR, Haddock SHD (2010) Three new species of remosiid siphonophore (Siphonophora: Physonectae). J Mar Biol Assoc UK 90 (6): 1119–1143.

[92] Pugh PR, Youngbluth MJ (1988) A new species of *Halistemma* (Siphonophora: Physonectae: Agalmidae) collected by submersible. J Mar Biol Assoc UK 68 (1): 1–14.

[93] Mapstone GM (2004) First full description of the large physonect siphonophore *Halistemma amphytridis* (Lesueur & Petit, 1807). Hydrobiologia 530/531: 231–240.

[94] Margulis RY (1993) *Cordagalma tottoni* sp.n. - a new siphonophore of the Suborder Physonectae (Cnidaria, Hydrozoa, Siphonophora). Zool Zhurnal 72 (9): 14–19.

[95] Hiscock K, Mapstone GM, Conway DVP, Halliday N (2010) Occurrence of the physonect siphonophore *Apolemia uvaria* off Plymouth and in south-west England. Marine Biodiversity Records 3: 1–4.

[96] Siebert S, Robinson MD, Tintori SC, Goetz F, Helm RR, et al (2011) Differential gene expression in the siphonophore *Nanomia bijuga* (Cnidaria) assessed with multiple next-generation sequencing workflows. Plos One 6 (7) 1–12: e22953 10.1371/journal.pone.0022953 PMC3146525 21829563

[97] Pugh PR (1998) A re-description of *Frillagalma vitiazi* Daniel 1966 (Siphonophorae, Agalmatidae). Sci Marina 62 (3): 233–245.

[98] Mills CE, Pugh PR, Harbison GR, Haddock SHD (1996) Medusae, siphonophores and ctenophores of the Alborán Sea, south western Mediterranean. In: Sci Marina 60 Piraino S, Boero F, Bouillon J, Cornelius PFS, Gili JM, editors. Advances in Hydrozoan Biology. (1): 145–163.

[99] Mapstone GM (2005) Re-description of *Rosacea cymbiformis*, a prayine siphonophore (from the Mediterranean Sea), with comments on nectophore designation and bract orientation. J Mar Biol Assoc UK 85 (3): 709–721.

[100] Mapstone GM, Pugh PR (2004) Case 3309. *Rosacea* Quoy & Gaimard, 1827: proposed conservation of usage (Cnidaria, Siphonophora); *Desmophyes annectens* Haeckel, 1888 and *Rosacea plicata* Bigelow, 1911: proposed conservation. Bull Zool Nomen 61 (3): 149–153.

[101] ICZN (2006) Opinion 2157. Bull Zool Nomencl 63 (3): 207–208.

[102] Pugh PR (1992) *Desmophyes haematogaster*, a new species of prayine siphonophore (Calycophorae, Prayidae). Bull Mar Sci 50 (1): 89–96.

[103] Pugh PR (1992) The status of the genus *Prayoides* (Siphonophora: Prayidae). J Mar Biol Assoc UK 72 (4): 895–909.

[104] Pugh PR (1991) Co-occurrence of hippopodiid siphonophores and their potential prey. Hydrobiologia 216/217: 327–334.

[105] Pugh PR (1995) *Clausophyes tropica* (Siphonophorae, Calycophora), a new siphonophore species from the tropical Atlantic. Bull Mar Sci 57 (2): 453–459.

[106] Pugh PR (2006) Reclassification of the clausophyid siphonophore *Clausophyes ovata* into the genus *Kephyes* gen. nov. J Mar Biol Assoc UK 86 (5): 997–1004.

[107] Lindsay DJ, Grossmann M, Minemizu R (2011) *Sphaeronectes pagesi* sp. nov., a new species of sphaeronectid calycophoran siphonophore from Japan, with the first record of *S. fragilis* Carré, 1968 from the North Pacific Ocean and observations on related species. Plankton & Benthos Research 6 (2): 101–107.

[108] Grossmann MM, Lindsay DJ, Fuentes V (2012) *Sphaeronectes pughi* sp. nov., a new species of sphaeronectid calycophoran siphonophore from the Subantarctic zone. Polar Sci 6: 196–199.

[109] Pagès F, Flood P, Youngbluth M (2006) Gelatinous zooplankton net-collected in the Gulf of Maine and adjacent submarine canyons: new species, new family (Jeanbouilloniidae), taxonomic remarks and some parasites. Sci Marina 70 (3): 363–379.

[110] Pugh PR, Pagès F (1997) A re-description of *Lensia asymmetrica* Stepanjants, 1970 (Siphonophorae, Diphyidae). Sci Marina 61 (2): 153–161.

[111] Pugh PR, Pagès F (1995) Is *Lensia reticulata* a diphyine species (Siphonophorae, Calycophora, Diphyidae)? A re-description. Sci Marina 59 (2): 181–192.

[112] Grossmann MM, Lindsay DJ, Collins AG (2013) The end of a garbage bin taxon: *Eudoxia macra* Totton, 1954, is the eudoxid stage of *Lensia cossack* Totton, 1941 (Siphonophora, Cnidaria). Syst Biodivers 11 (3): 381–387.

[113] Pugh PR (1999) Siphonophorae. In: Boltovsky D, editor. South Atlantic Zooplankton I. Leiden, The Netherlands: Backhuys Publishers. 467–511.

[114] Thibault-Botha D, Gibbons MJ (2005) Epipelagic siphonophores off the east coast of South Africa. Afr J Mar Sci 27 (1): 129–139.

[115] Sears M (1953) Notes on siphonophores 2. A revision of the Abylinae. Bull Mus Comp Zool 109 (1): 1–119.

[116] Grossmann MM, Lindsay DJ (2013) Diversity and distribution of the Siphonophora (Cnidaria) in Sagami Bay, Japan, and their association with tropical and subarctic water masses. Journal of Oceanography Japan 64 (4): 395–411 10.1007/s10872-013-0181-9

[117] Pagès F, White MG, Rodhouse PG (1996) Abundance of gelatinous carnivores in the nekton community of the Antarctic Polar Front Zone in summer 1994. Mar Ecol Prog Ser 141 (1–3): 139–147.

[118] Pagès F, Kurbjeweit F (1994) Vertical distribution and abundance of mesoplanktonic medusae and siphonophores from the Weddell Sea, Antarctica. Polar Biol 143: 243–251.

[119] Pagès F, Pugh PR, Gili JM (1994) Macro- and megaplanktonic cnidarians collected in the eastern part of the Weddell Gyre during summer 1979. J Mar Biol Assoc UK 74 (4): 873–894.

[120] Pagès F, Schnack-Schiel SB (1996) Distribution patterns of the mesozooplankton, principally siphonophores and medusae, in the vicinity of the Antarctic Slope Front (eastern Weddell Sea). J Mar Syst 9: 231–248.

[121] Fautin DG (2009) Structural diversity, systematics and evolution of cnidae. Toxicon 54: 1054–1064. 19268491 10.1016/j.toxicon.2009.02.024

[122] Weill R (1934) Contribution à l’étude des cnidaires et de leurs nématocystes. I. Trav Stn Zool Wimereux 10: 1–347.

[123] Werner B (1965) Die Nesselkapseln der Cnidaria, mit besonderer Beruchsichtigung der Hydroida. 1. Klassifikation und Bedeutung fur die Systematik und Evolution. Helgolander Wissenschaftliche Meeresuntersuchungen 12: 1–39.

[124] Östman C (2000) A guideline to nematocyst nomenclature and classification, and some notes on the systematic value of nematocysts. Sci Marina 64 (Suppl. 1)31–46.

[125] Mackie GO (1960) Studies on *Physalia physalis*. Part II. Behaviour and histology. Discovery Reports 30: 371–408.

[126] Carré D (1974) Formation, migration et maturation des nématoblastes et des nématocytes chez les siphonophores. I. Mise en évidence et formation des clones de nématocystes. Annales d’Embryologie et de Morphogenese 7 (2): 205–218.

[127] Carré C, Carré D (1973) Étude du cnidome et de la cnidogenese chez *Apolemia uvaria* (Lesueur, 1811) (Siphonophore physonecte). Exp Cell Res 81: 237–249. 4148393 10.1016/0014-4827(73)90130-4

[128] Vogt C (1854) Recherches sur les animaux inférieurs de la Méditerranée. 1. Sur les Siphonophores de la mer de Nice. Mém Inst natn genèv 1: 1–164.

[129] Wrobel D *Physophora hydrostatica*, Jellies Zone website. Available: http://jellieszone.com/physophora.htm. Accessed 2014 Jan 6.

[130] Carré C (1968) Description d’un siphonophore Agalmidae, *Cordagalma cordiformis* Totton, 1932. Beaufortia 16 (212): 79–86.

[131] Pugh PR, Harbison GR (1986) New observations on a rare physonect siphonophore, *Lychnagalma utricularia* (Claus, 1879). J Mar Biol Assoc UK 66 (3): 695–710.

[132] Carré C (1969) *Rosacea villafrancae* sp. n., un nouveau siphonophore calycophore Prayinae de la mer Méditerranée. Beaufortia 214 (16): 109–117.

[133] Carré C (1969) *Prayola tottoni* gen. sp. n., nouveau genre et nouvelle espèce de siphonophore calycophore Prayinae de la mer Méditerranée. Vie Milieu Paris 20: 30–42.

[134] Carré C (1969) Sur le genre *Lilyopsis* Chun 1885, avec une redescription de l’èspece *Lilyopsis rosea* Chun 1885 (Siphonophore, Prayinae) et une description du sa phase calyconula. Cah Biol Marine 10: 71–81.

[135] Marques AC, Collins AG (2004) Cladistic analysis of Medusozoa and cnidarian evolution. Invertebr Biol 123 (1): 23–42.

[136] Hessinger DA, Ford MT (1988) Ultrastructure of the small cnidocyte of the Portuguese man-of-war (*Physalia physalis*) tentacle. In: Hessinger DA, Lenhoff HM, editors. The Biology of Nematocysts. San Diego: Academic Press Inc. 75–94.

[137] Will L (1909) Uber das Vorkommen kontraktiler Elemente in den Nesselzellen der Coelenteraten. Sitzungsberichte und Abhandlungen der Naturforschenden Gesellschaft zu Rostock 1: 33–52.

[138] Bouillon J, Medel MD, Pagès F, Gili JM, Boero F, et al (2004) Fauna of the Mediterranean Hydrozoa. Sci Marina 68 (Suppl. 2)5–438.

[139] Purcell JE (1981) Feeding ecology of *Rhizophysa eysenhardti*, a siphonophore predator of fish larvae. Limnol Oceanogr 26 (3): 424–432.

[140] Purcell JE (1981) Dietary composition and diel feeding patterns of epipelagic siphonophores. Mar Biol 65 (1): 83–90.

[141] Biggs DC (1977) Field studies of fishing, feeding and digestion in siphonophores. Mar Behav Physiol 4 (4): 261–274.

[142] Skaer RJ (1988) The formation of cnidocyte patterns in siphonophores. In: Hessinger, DA, Lenhoff HM, editors. The Biology of Nematocysts. San Diego, Academic Press. 165–178.

[143] Kramp PL (1942) Siphonophora. The Godthaab Expedition 1928. Medd Grønland 80 (8): 3–24.

[144] Kawamura T (1911) “Baren kurage” (*Physophora hydrostatica* Forsk.) Zool Mag (Tokyo) (Dobuts Zhasshi) 23 (6): 309–323, plate 6 (In Japanese only).

[145] Totton AK (1955) Development and metamorphosis of the larva of *Agalma elegans* (Sars) (Siphonophora Physonectae). In “Papers in Marine Biology and Oceanography”. Graham M, editor. Deep Sea Res Part 2 Top Stud Oceanogr 3 (Suppl): 239–241.

[146] Russell FS (1939) On the nematocysts of hydromedusae II. J Mar Biol Assoc UK 23 (2): 347–359.

[147] Fewkes JW (1881) Studies on the Jelly-fishes of Narragansett Bay. Bull Mus Comp Zool 8 (8): 141–182.

[148] Biggs DC (1978) *Athorybia lucida*, a new species of siphonophore (Physonectae, Athorybiidae) from the North Atlantic Ocean. Bull Mar Sci 28 (3): 537–542.

[149] Fewkes JW (1888) Studies from the Newport Marine Zoölogical Laboratory. XIX. On certain medusae from New England. Bull Mus Comp Zool 13 (7): 209–240.

[150] Bigelow HB (1931) Siphonophorae from the Arcturus Oceanographic Expedition. Zoologica (New York) 8 (11): 525–592.

[151] Chun C (1891) Die Canarischen Siphonophoren in monographischen Darstellungen. I. *Stephanophyes superba* und die Familie der Stephanophyiden. Abh Senckenb Naturforch Ges 16: 553–627.

[152] Stepanjants SD (1967) Siphonophores of the seas of the USSR and the northern part of the Pacific Ocean. Opredeliteli po Faune SSSR 96. Leningrad SSSR: Akademiya Nauk. 1–96.

[153] Bigelow HB (1918) Some Medusae and Siphonophorae from the Western Atlantic. Bull Mus Comp Zool 62 (8): 365–442.

[154] Chun C (1892) Die Canarischen Siphonophoren in monographischen Darstellungen. II. Die Monophyiden. Abh Senckenb Naturforch Ges 18: 57–144.

[155] Mackie GO, Marx RM (1988) Phosphatic spicules in the nematocyst batteries of *Nanomia cara* (Hydrozoa, Siphonophora). Zoomorphology 108 (2): 85–91.

[156] Plachetzki D, Fong CR, Oakley TH (2012) Cnidocyte discharge is regulated by light and opsin-mediated phototransduction. BMC Biol 2012 10: 17 10.1186/1741-7007-10-17 PMC3329406 22390726

[157] Purcell JE (1980) Influence of siphonophore behaviour upon their natural diets: evidence for aggressive mimicry. Science 209 (4460): 1045–1047. 10.1126/science.209.4460.1045 17747233

[158] Bedot M (1904) Siphonophores provenant des campagnes du yacht Princess-Alice (1892–1902). Resultats des Campagnes Scientifiques accomplies par le Prince Albert I Monaco 27: 1–27.

[159] Haddock SHD (2006) Luminous marine organisms. In: Daunert S, Deo SK, editors, Photoproteins in Bioanalysis. Wenheim, Germany: Wiley-VCH. 25–47.

[160] Haddock SHD, Moline MA, Case JF (2010) Bioluminescence in the sea. Ann Rev Mar Sci 2: 443–493. 10.1146/annurev-marine-120308-081028 21141672

[161] Shimomura O (2006) Bioluminescence. Singapore: World Scientific. 470 p.

[162] Haddock SHD, McDougall CM, Case JF The Angler jellyfish, The Bioluminescent Web Page website. Available: http://biolum.eemb.ucsb.edu/organism/erenna.html. Accessed 2014 Jan 9.

