# Correction: Global Diversity and Review of Siphonophorae (Cnidaria: Hydrozoa)

**DOI:** 10.1371/journal.pone.0118381

**Published:** 2015-02-06

**Authors:** Gillian Mapstone

This article included several personal observations by Drs Dhugal Lindsay and Philip Pugh that the author published without their permission. The author offers unreserved apologies to Drs Lindsay and Pugh for the un-authorized use of the information and would like to make the following corrections to the text:

Fig. 1 was based on an unpublished histogram produced by Dr Pugh, and has been reproduced without his permission. The figure legend is corrected as follows: “Principle researchers and others from mid-18^th^ century to the present, derived from an unpublished histogram developed by Dr Pugh. Authors identified only by initials are Q & G: Quoy and Gaimard, K & E: Keferstein and Ehlers, and L & van R: Lens and van Riemsdijk”.

The author corrects the following statement in the section on Apolemiidae: “Currently, the family is monotypic for *Apolemia*, and includes *A. uvaria* (Lesueur, 1815), *A. vitiazi* (Stepanjants, 1967) and *A. contorta* (Margulis, 1976) [1], together with two newly described species *A. lanosa* and *A. rubriversa* [11] and a third species not yet described (*A. trinegra* [84])”. Reference 84 does not mention *A. trinegra* as the name of an undescribed species; that name having been made known to her by Dr Lindsay on a personal basis and published without his permission. The author offers her apologies and corrects the statement to read: “Currently, the family is monotypic for *Apolemia*, and includes *A. uvaria* (Lesueur, 1815), *A. vitiazi* (Stepanjants, 1967) and *A. contorta* (Margulis, 1976) [1], together with two newly described species *A. lanosa* and *A. rubriversa* [11] and a third species as yet undescribed [84]”.

The following statement in the section “Unascribed monoecious physonects” is incorrect: “Two new species await description in the genus *Cordagalma* [17], and a re-description of *F. vityazi* from new submersible material shows that frilling of the ridges in the nectophores and bracts of the original net-caught specimens is a preservation artefact [97]”. The author acknowledges that reference 17 made no mention of two new *Cordagalma* species awaiting description, the author had access to the information from Dr Pugh and this was published without his permission. This statement should read as follows: “A re-description of *F. vityazi* from new submersible material shows that frilling of the ridges in the nectophores and bracts of the original net-caught specimens is a preservation artefact [97]”.

In the same section, the following sentence was based on information provided in a personal communication with Philip Pugh and was published without his permission: “Fresh specimens of a fourth unassigned monoecious physonect, *Rudjakovia plicata*, taken recently off California indicate that their much pleated nectosacs are also preservation artefacts”. Once again the author offers her apologies and acknowledges that this information should not have been included in the article.

Also, the following sentence should be attributed to Reference 17: “The nectophores of this species attach to the dorsal side of the nectosome, indicating that it may be referable to the Agalmatidae sensu stricto, but further material is needed to confirm this hypothesis”. The corrected sentence should read: “The nectophores of this species attach to the dorsal side of the nectosome, indicating that it may be referable to the Agalmatidae *sensu stricto*, but further material is needed to confirm this hypothesis [17]”.

The following statement in the section on Clausophyidae is inaccurate as references 10 and 106 mention one and not two new species in the family Clausophyidae awaiting description: “New deep-water records from various locations worldwide contribute further to our understanding of the ecology of this deep-water family [6], [42], [47], [87], [105], [106], [109], [116], [117], [118], [119], [120], and two further new clausophyid species await description [10], [106]”. As clarification on the item being corrected, the author notes that the following new publication does mention a second as yet unnamed clausophyid in the genus *Kephyes*:

Lindsay DJ, Umetsu M, Grossmann M, Miyake H, Yamamotu H (2015) The Gelatinous Macroplankton Community at the Hatoma Knoll Hydrothermal Vent. In: J. Ishibashi et al., editors. Subseafloor Biosphere Linked to Hydrothermal Systems: TAIGA Concept, DOI 10.1007/978-4-431-54865-2_51.

All novel information and derived conclusions in the section on Rhodaliidae are based on information communicated by Dr Dhugal Lindsay in confidence and have been presented without his permission. The author is deeply sorry for this transgression. At the time of publication this information was only known to the author through personal communication with Dr Dhugal Lindsay and she would like to apologize for the inclusion of this information without his permission.

Additional corrections

In addition to the corrections above, the author acknowledges that the following statement in the Introduction is misleading: “Siphonophores are holoplanktonic, except for rhodaliids which can transiently attach their tentacles to the substrate, and thus lack the true benthic stage that is characteristic of the life cycle of many hydromedusae and other colonial cnidarians”. The author corrects it to “Siphonophores are holoplanktonic (except for rhodaliids which mostly attach their tentacles to the substrate) and thus lack the true benthic stage that is characteristic of the life cycle of many hydromedusae and other colonial cnidarians.

Finally, Fig. 2A presents Anthozoa species numbers as Octocorallia c.3 000 and Hexacorallia c.4 300, based on reference [59]. The author acknowledges the estimated number of Hexacorallia species is incorrect and should read Hexacorallia c.3 300 [see Hexacorallians of the World website http://hercules.kgs.ku.edu/Hexacoral/Anemone2/nmita.cfm]. Dr Stephen Cairns (Department of Invertebrate Zoology, Smithsonian Institution) confirmed the accuracy of the octocorallian estimate.
